# Detection and application of genome-wide variations in peach for association and genetic relationship analysis

**DOI:** 10.1186/s12863-019-0799-8

**Published:** 2019-12-30

**Authors:** Liping Guan, Ke Cao, Yong Li, Jian Guo, Qiang Xu, Lirong Wang

**Affiliations:** 1 Zhengzhou Fruit Research Institute, Chinese Academy of Agricultural Sciences, Zhengzhou, 450009 People’s Republic of China; 2 Key Laboratory of Horticultural Plant Biology (Ministry of Education), Huazhong Agricultural University, Wuhan, 430070 People’s Republic of China

**Keywords:** Genome-wide variations, Detection, Application, Peach

## Abstract

**Background:**

Peach (*Prunus persica* L.) is a diploid species and model plant of the *Rosaceae* family. In the past decade, significant progress has been made in peach genetic research via DNA markers, but the number of these markers remains limited.

**Results:**

In this study, we performed a genome-wide DNA markers detection based on sequencing data of six distantly related peach accessions. A total of 650,693~1,053,547 single nucleotide polymorphisms (SNPs), 114,227~178,968 small insertion/deletions (InDels), 8386~12,298 structure variants (SVs), 2111~2581 copy number variants (CNVs) and 229,357~346,940 simple sequence repeats (SSRs) were detected and annotated. To demonstrate the application of DNA markers, 944 SNPs were filtered for association study of fruit ripening time and 15 highly polymorphic SSRs were selected to analyze the genetic relationship among 221 accessions.

**Conclusions:**

The results showed that the use of high-throughput sequencing to develop DNA markers is fast and effective. Comprehensive identification of DNA markers, including SVs and SSRs, would be of benefit to genetic diversity evaluation, genetic mapping, and molecular breeding of peach.

## Background

Peach (*Prunus persica*, 2n = 16) is a member of the family *Rosaceae*, subfamily *Prunoideae*, and currently is widely grown in China, America, Italy, Spain, Japan, and other countries. In the past decade, the development of molecular markers for use in plant genetic research has become essential for geneticists and breeders. Thus, various approaches have been explored and applied in peach, such as restriction fragment length polymorphism markers [1], random amplified polymorphic DNA markers [1], and amplified fragment length polymorphism markers [2].

Recently, via next-generation sequencing (NGS) technology, entire genomes have been resequenced efficiently and economically to identify many polymorphic DNA markers, including single nucleotide polymorphisms (SNPs), small insertion/deletions (InDels), structure variants (SVs), copy number variants (CNVs), and simple sequence repeats (SSRs), which have been widely used for genetic diversity analyses and the construction of linkage maps of rice [3], eggplant [4], watermelon [5], and Chinese cabbage [6]. Among them SNPs were recognized as important due to their abundance, codominance, efficiency, and ease of automation. Furthermore, SNPs have been widely used in the construction of high-density genetic maps and in genome-wide association studies (GWAS), which require abundant markers. For example, a total of 4,063,377 SNPs were used to perform GWAS for 12 agronomic traits of peach to identify candidate genes and design molecular markers [7]. SSRs are one of the most commonly used markers in many genetic applications since the early 1990s. Because of their reproducibility, codominance, relative abundance, high genome coverage and versatile platforms for genotyping [8], SSRs have been recognized as a valuable molecular marker for fingerprinting [9] and genetic diversity analyses [10].

With the rapid development of NGS technologies, whole-genome sequencing provides large amounts of DNA markers information in many plant species. For instance, a total of 4,980,259 SNPs, 1,026,375 INDELs and 159,330 SVs were identified through whole genome re-sequencing of 480 peach accessions [11]. Sun et al. (2013) obtained a total of 200,627 SNPs, 4900 InDels, and 7063 SSRs in two cultivars of mei [12]. In the present study, we detected a large number of putative polymorphic markers, including SNPs, InDels, SVs, CNVs and SSRs by performing high-depth whole-genome re-sequencing of six peach accessions. We further did functional annotation for these DNA markers. In addition, 944 SNPs were filtered for association study of fruit ripening time and 15 highly polymorphic SSRs were selected to analyze the genetic relationship among 221 accessions. The present study provides a large set of polymorphic markers among landraces and improved varieties of peach, and our results may facilitate peach molecular breeding in the future.

## Results

### Sequence mapping

Through sequencing of six distantly related peach accessions, we generated a total of 107.35 Gb base pairs of sequences. Then, the data were filtered by the following 2 steps: first, the adapter contaminants in the reads were deleted, and then, the reads that contained more than 50% low-quality bases (quality value <= 12) were removed. A total of 105.85 G high quality clean reads were obtained for the following analysis. The reads were then aligned to the peach reference genome (224.61 Mb) using BWA software. The mapping rate in different accessions varied from 93.16 to 96.69%, and the final effective sequencing depth varied from 63.81 to 89.38 × (Table 1).

**Table 1:** Summary of the sequencing results of the six peach accessions

Sample	Species	Origin	Population	Raw bases (G)	Clean bases (G)	Coverage rate (%)	Sequencing depth (×)
Ka Shi Huang Rou Li Guang	*P. persica*	Sinkiang, PRC	Edible landrace	15.04	14.98	97.83	63.81
Ji Mi Xia Ye Tao	*P. persica*	Henan, PRC	Improved variety	19.87	19.77	97.29	87.77
Xin Jiang Pan Tao 2	*P. ferganensis*	Henan, PRC	Edible landrace	18.90	18.26	96.80	64.65
Xia Miao 1	*P. persica*	Shaanxi, PRC	Edible landrace	20.22	20.06	98.08	89.38
Sa Hua Hong Pan Tao	*P. persica*	Shanghai, PRC	Edible landrace	17.69	17.67	96.85	76.06
Wu Yue Xian Bian Gan	*P. persica*	Beijing, PRC	Edible landrace	15.63	15.11	97.17	69.15

### Variation detection and annotation

Pair-end reads were mapped against the peach reference genome [13] (release version 1.0), and a final set of 1,166,551 SNPs, 44,245 SVs, 12,302 CNVs, and 141,895 SSRs were identified, resulting in an average of 5351.1 SNPs per Mb, 202.71 SVs per Mb, 56.4 CNVs per Mb, and 634.43 SSRs per Mb, respectively. Based on the consensus sequence, the polymorphic loci between the identified genotype and the reference were filtered. The polymorphic DNA markers were classified into five groups: SNPs, InDels, SVs, CNVs and SSRs.

**Fig. 1:**
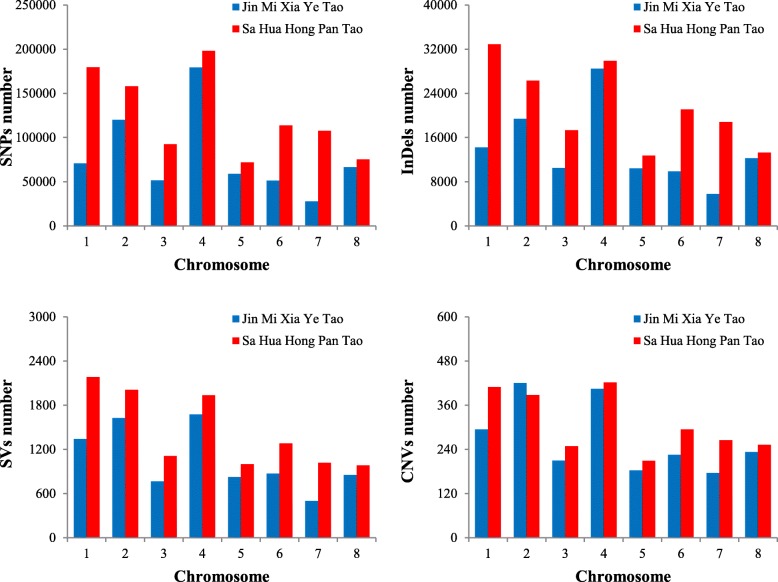
The number of genomic variations in eight chromosomes of two peach accessions

**Fig. 2:**
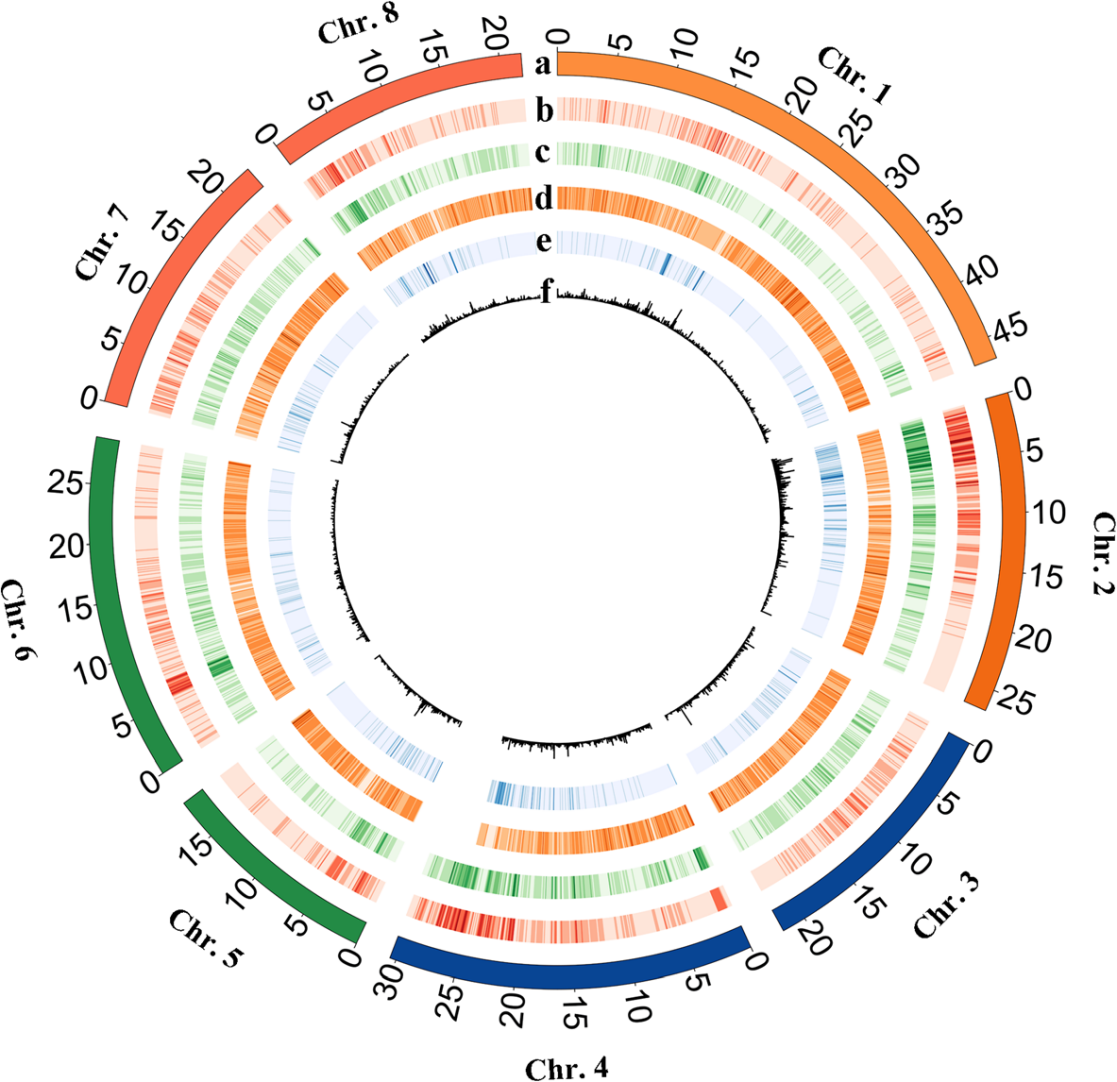
Distribution plot of variations in eight chromosomes of six peach accessions. All tracks are plotted in 100 kb windows. (**a**) The physical length of each chromosome. (**b**) SNPs in the six peach accessions along the chromosomes. (**c**) InDels in the six peach accessions along the chromosomes. (**d**) SSRs in the six peach accessions along the chromosomes. (**e**) CNVs in the six peach accessions along the chromosomes. (**f**) SVs in the six peach accessions along the chromosomes

Next, we analysed the number of genetic variation across each chromosome. We found that the number of polymorphic DNA markers varied across each chromosome (Fig. 1). Most of them were observed in Chr. 1, 2, and 4. For example, the number of SNPs in ‘Jin Mi Xia Ye Tao’ (179,310) and ‘Sa Hua Hong Pan Tao’ (198,140) of Chr. 4 were 6.43- and 2.76- fold respectively higher than the number of SNPs in ‘Jin Mi Xia Ye Tao’ of Chr. 7 (27,867) and in ‘Sa Hua Hong Pan Tao’ of Chr. 5 (71,790). The uneven marker distribution of each chromosome can be attributed to the variations in chromosome size in the peach genome. Chr. 4 was found to be 30.19 Mb in size, which was 1.33-fold the size of Chr. 7 (22.72 Mb) and was 1.64-fold that of Chr. 5 (18.45 Mb). Finally, all the polymorphic DNA markers were compared at population level to detect SNPs, InDels, SVs, and CNVs. We showed the distribution of these variatons at population level in reference genome by figures (Fig. 2).

### The detailed genome-wide characterization of SNPs

The SNP annotation showed that approximately 4.49 to 7.28% of the total SNPs were located in the coding DNA sequence (CDS) of these six genomes (Table 2). These variations were minimal but had a substantial impact on the variation in genomes and biological traits. Therefore, it is helpful to examine the detailed SNP annotations. In this study, 27,623 synonymous and 40,245 nonsynonymous SNPs were annotated in ‘Sa Hua Hong Pan Tao’, and only 17,902 synonymous and 26,076 nonsynonymous SNPs were annotated in ‘Wu Yue Xian Bian Gan’ (Additional file 1: Figure S1a). These nonsynonymous SNPs have been suggested as good candidate mutations to explain the different phenotypes among different samples. The ratio of nonsynonymous to synonymous substitutions was 1.46~1.52, which is higher than that of *Arabidopsis thaliana* (0.83) [13] and rice (1.29) [14] and similar to our previous report in peach (1.63) [15]. In addition, we detected 114,227~178,968 InDels, 8386~12,298 SVs, and 2111~2581 CNVs among the six peach genomes (Table 3). Similar to the annotation of SNPs, only minimal distributions of InDels, SVs, and CNVs were located in the CDS.

**Table 2:** Genomic distribution of SNPs identified in the six peach accessions

Distribution	Ka Shi Huang Rou Li Guang	Jin Mi Xia Ye Tao	Xin Jiang Pan Tao 2	Xia Miao 1	Sa Hua Hong Pan Tao	Wu Yue Xian Bian Gan
CDS	47,354	45,967	53,720	57,999	67,868	43,978
5′-UTR	1811	1653	1976	2119	2534	1693
3′-UTR	3335	3010	3320	3870	4643	2894
mRNA	124,200	121,499	141,988	152,480	181,150	118,274
Total	711,669	650,693	836,939	860,961	1,053,547	686,241

**Table 3:** Indels, SVs, and CNVs annotation in the six peach accessions

Annotation		Ka Shi Huang Rou Li Guang	Jin Mi Xia Ye Tao	Xin Jiang Pan Tao 2	Xia Miao 1	Sa Hua Hong Pan Tao	Wu Yue Xian Bian Gan
InDels	Insertion	59,642	55,539	74,791	72,646	87,492	79,369
	Deletion	62,357	58,688	77,136	75,862	91,476	82,699
	Total	121,999	114,227	151,927	148,508	178,968	162,068
SVs	Insertion	508	671	200	255	482	686
	Deletion	2765	2844	3199	3473	4469	3868
	Others	5113	5585	8042	5982	7347	5409
	Total	8386	9100	11,441	9710	12,298	9963
CNVs	Duplication	555	536	431	538	638	560
	Deletion	1771	1689	1707	1573	1943	1657
	Total	2326	2225	2138	2111	2581	2217

Duplication: the events in which the copy number increased

We further analyzed the annotation of the so-called large-effect SNPs (Additional file 1: Figure S1b), which are predicted to have a potentially disabling effect on gene function. We identified a total of 1943 SNPs that were expected to induce premature stop codons (designated as stop codon gain), 789 to disrupt splicing donor or acceptor sites, 170 to alter initiation methionine residues (start codon loss), and 217 SNPs to remove the annotated stop codons (stop codon loss), resulting in longer open reading frames. Based on the GO term annotation, 89.66% (203 genes) of these genes containing large-effect SNPs were assigned to one or more functional annotations. There were 144 GO terms associated with biological process, 68 with cellular component, and 157 with molecular function. Compared with the total annotated genes in the peach genome, the genes that grouped into localization and metabolic processes were enriched (Additional file 1: Figure S2).

We further investigated the tissue-specific expression of the genes contained large-effect SNPs and in root, fruit, phloem, leaf and seed in a representative cultivar, ‘Chinese Cling’ (Additional file 1: Figure S3). We found that gene expression pattern were different among various tissues and could be classified then into four groups. Among 158 differential expression genes, a total of 9, 13, 22, 17, and 8 genes that showed higher expression in leaves, fruits, seeds, root, and phloem than the other tissues, respectively.

### The detailed genome-wide characterization of SSRs

**Fig. 3:**
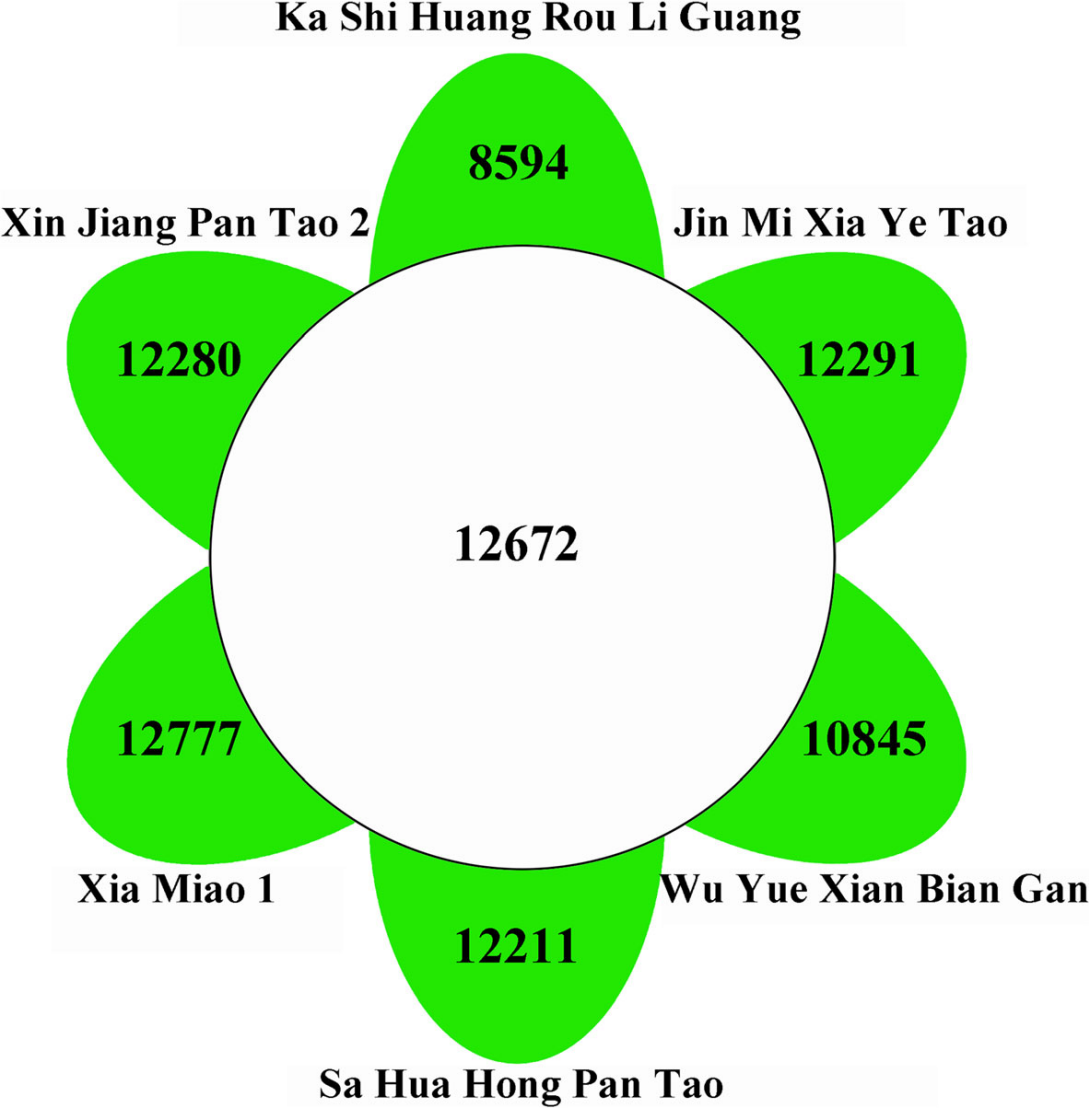
Venn diagram of SSRs located in the six accessions

Totally, 141,895 SSR loci were firstly identified in the six peach accessions, and 8.93% (12,672) of them were found to be detected in all six accessions (Fig. 3).

Among all SSRs, mono-nucleotide and di-nucleotide repeats were abundant, accounting for 47.36 and 39.87%, respectively (Table 4). Tri-, tetra-, penta-, and hexa-nucleotide repeats only accounted for 7.86, 1.48, 2.17, and 1.26%, respectively, for all SSRs. And the similar distribution of different repeats type was repoted in peach [16, 17] and mei [12].

**Table 4:** The summary of different nucleotide repeats of SSRs identified among six peach accessions

Repeat unit	Repeat type	Number	Frequency (%)
Mononucleotide	Total	67,196	47.36%
Dinucleotide	AT/TA	18,145	12.79%
	CT/TC	16,557	11.67%
	AG/GA	16,107	11.35%
	GT/TG	2926	2.06%
	AC/CA	2779	1.96%
	CG/GC	61	0.04%
	Total	56,575	39.87%
Trinucleotide	AAT/ATA/TAA	1642	1.16%
	TTA/TAT/ATT	1487	1.05%
	TTC/TCT/CTT	1332	0.94%
	AAG/AGA/GAA	1319	0.93%
	GGT/GTG/TGG	676	0.48%
	GGA/GAG/AGG	531	0.37%
	CCA/CAC/ACC	485	0.34%
	AAC/ACA/CAA	484	0.34%
	CCT/CTC/TCC	473	0.33%
	TTG/TGT/GTT	425	0.30%
	GGC/GCG/CGG	138	0.10%
	CCG/CGC/GCC	85	0.06%
	Others	2090	1.47%
	Total	11,158	7.86%
Tetranucleotide	Total	2102	1.48%
Pentanucleotide	Total	3074	2.17%
Hexanucleotide	Total	1790	1.26%

Of the di-nucleotide repeats, AT/TA repeats were the most abundant, accounting for 12.79%. And AAT/ATA/TAA/TTA/TAT/ATT was also abundant of all tri-nucleotide repeats, accounting for 2.21% of all SSRs. Among di-nucleotide repeats, the second largest group was CT/TC, slight higher than AG/GA repeats. Meanwhile, we found that TTC/TCT/CTT/CCT/CTC/TCC repeats accounted for 1.27% of all SSRs, also slight lower than AAG/AGA/GAA/GGA/GAG/AGG (1.30%) of all tri-nucleotide repeats. CG/GC and CGC/GCG/CGG/CCG/CGC/GCC were the least repeats of di-nucleotide and tri-nucleotide repeats, respectively. The result indicated A/T nucleotide exhibited a strong bias among SSRs, similar with the study reported before [8, 18].

Of all SSRs, 25,037 (17.64%) were located in the 7773 genes. Among them, the number of SSRs in CDS was 8412 (33.60%), and there were 800 in 5’untranslated region (UTR), 630 in 3’UTR (Additional file 1: Table S2). We also analysed the SSR motifs in different regions. Of all SSRs located in UTR regions, di-nucleotide repeats were the most abundant, accounting for 46.36% of all motifs (Additional file 1: Table S2). AG/GA (290) and CT/TC (266) were the most and the next was AT/TA (54), AC/CA (30), and GT/TG (23). No CG/GC repeats were found in these regions. However, among SSRs located in CDS regions (Additional file 1: Table S2), the di-nucleotide repeats account for 28.27%, was lower than that of mono-nucleotide repeats (51.70%). AG/GA and CT/TC dimers prevailed in CDS sequences, reached 776 and 720, respectively.

**Fig. 4:**
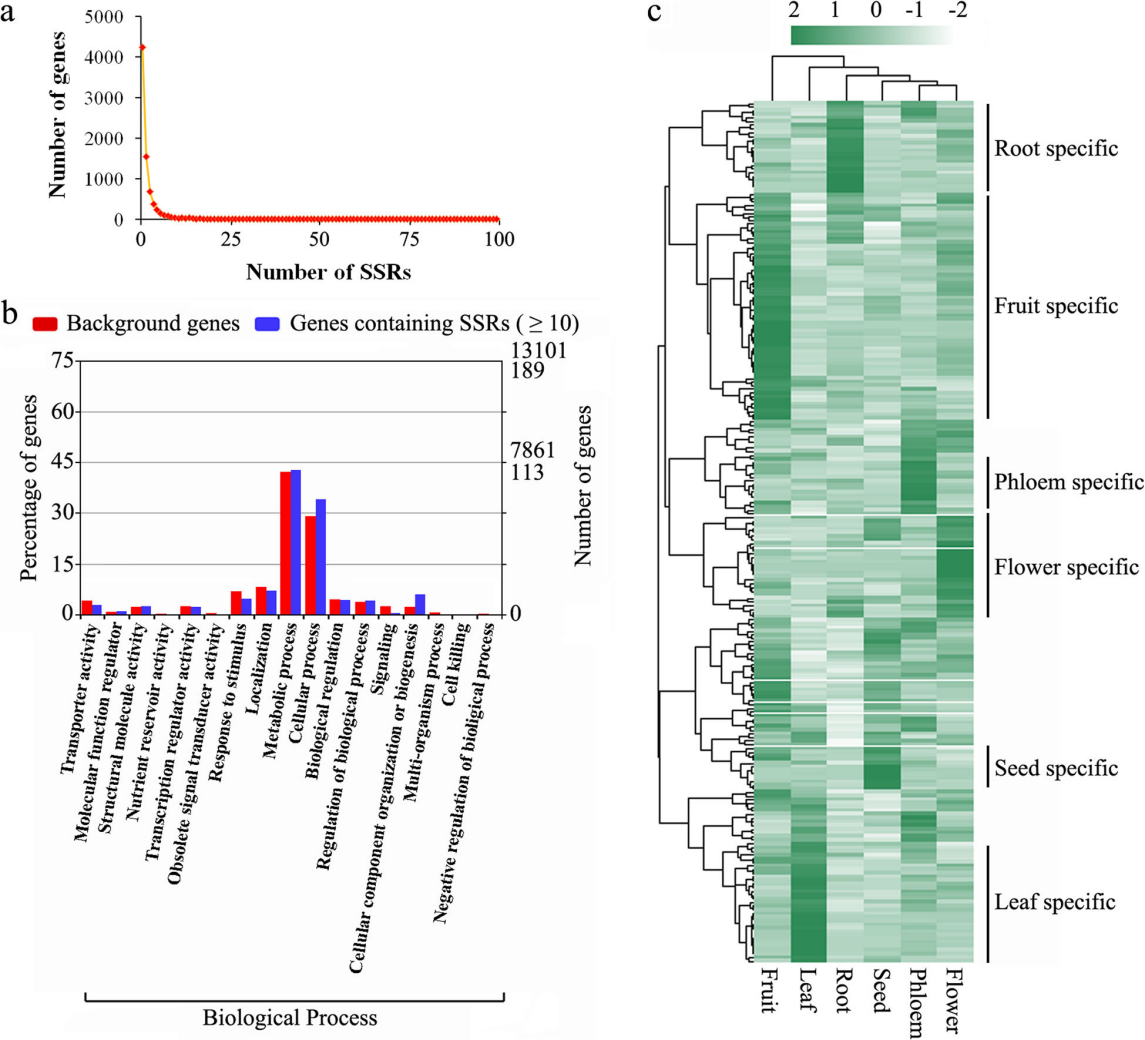
Overview of SSR-containing genes in the peach genome. (**a**) Distribution of SSR-containing gene numbers versus SSR number. (**b**) GO annotation of genes containing more than 10 SSRs in the peach genome. (**c**) Expression patterns of genes with more than 10 SSRs (235). The heat maps show the log_2_ FPKM values of genes in different peach tissues, including root, phloem, leaf, flower, fruit, and seed tissues

The SSR-containing genes should be further investigated to study the variation of agronomic character in peaches. Therefore, we first counted the SSR number in each gene and found that 4247 of 7773 genes contained only one SSR and 331 genes contained more than ten loci, and this may be related to the size of each genic region. A sharply decreasing trend of gene number was observed as the contained SSR number increased (Fig. 4a). The detailed GO annotation of above 331genes identified in this study is shown in Fig. 4b. The expression of all genes which containing more than 10 SSRs was further quantified using FPKM values, and 235 (71.0%) genes had an FPKM value > 1 in at least one tissue. The heat map results showed that most genes presented tissue-specific expression patterns (Fig. 4c). In general, the fruit has the greater number of SSRs than the leaf, flower, and root tissues. And in phloem and seed, the tissue-specific genes were fewest. The result indicated that the phenotype variations in fruit are mostly result from SSR polymorphism than the other tissues.

### Association study of fruit maturity date using SNPs

Based on previous studies in peach [19], three quantitative trait loci for maturity date were detected and all located on Chr. 4 between two SNPs, SNP_IGA_405773 (Chr. 4: 9,658,797 bp) and SNP_IGA_437516 (Chr. 4:17,094,116 bp). Filtering with missing rate, MAF and sequencing depth parameters, we totally obtained 25,299 SNPs from 9.6 Mb to 17.1 Mb on Chr. 4. Furthermore, considering that linkage disequilibrium decay was about 20–50 kb for the different subgroups of cultivated peach [15], a total of 375 loci were thought enough for obtaining reliable association result. To increase the accurary of result, 944 SNPs (about 7-kb intervel) were filtered for association studies of fruit maturity date in the following analysis.

**Fig. 5:**
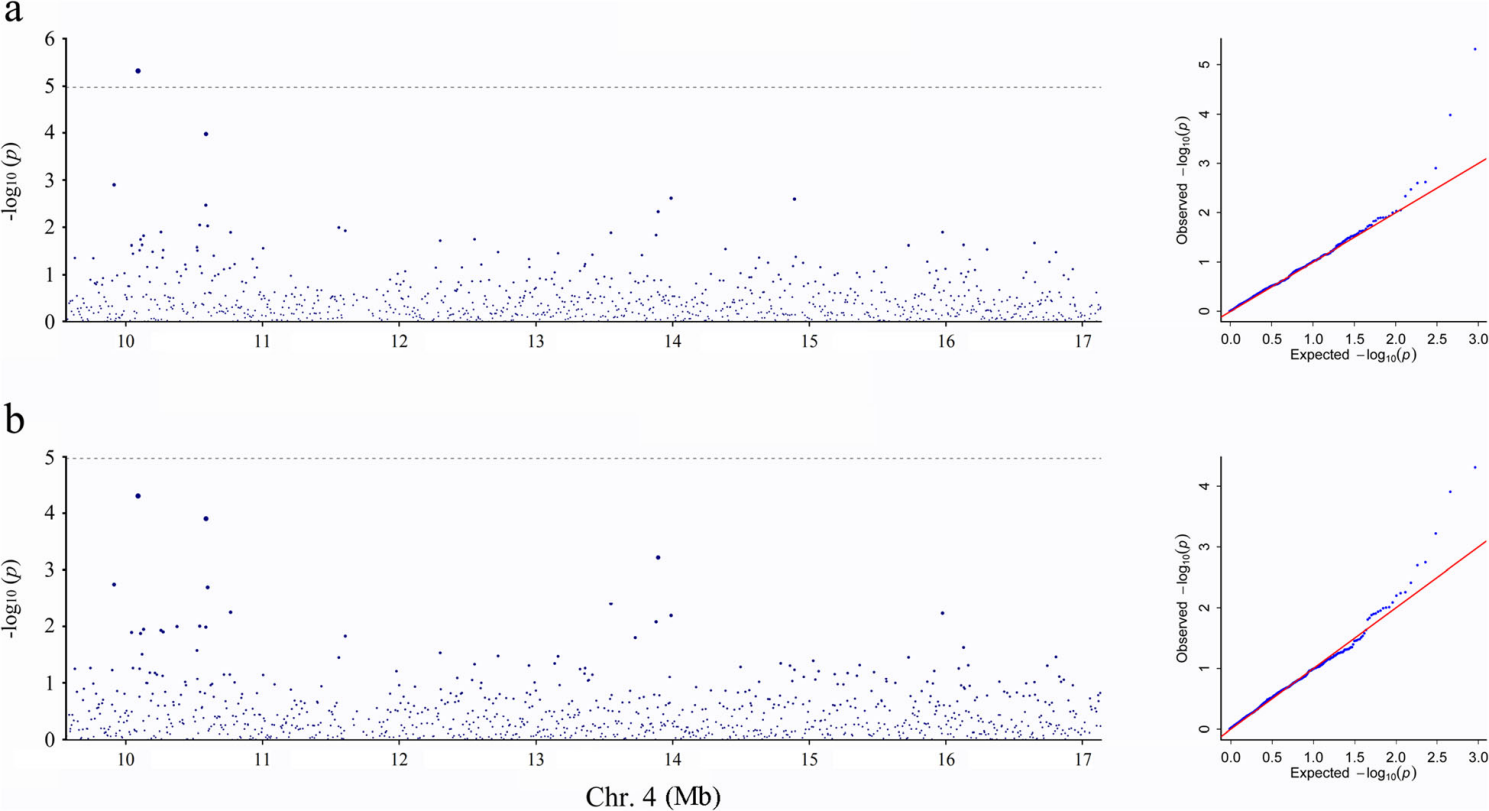
Association results for fruit maturity date evaluated in 2011 (**a**) and 2012 (**b**) in peach. Genomic position in Chr. 4 (x-axis) is plotted against its significance expressed as -log_10_ (*P*) value (y-axis). The black dotted horizontal line indicates the significance threshold (−log_10_*P* = 4.97)

We identified a clear year-stable signal in 2011 and 2012 associated with fruit maturity date on Chr. 4, although the association signal in 2012 was lower than the adjusted *p* value (−log_10_*P* > 4.97, Fig. 5). The leading SNP (Chr.4: 10,184,313 bp, −log_10_*P* = 5.32) of this association was found in the exon of *Prupe.4G171900* which encoded a glutamate receptor protein. And the location has a close distance (< 1 Mb) with the candidate gene *Prupe.4G186800* of fruit maturity by another study [20], proving the association result is reliable.

### Genetic diversity assessment using SSRs

To detect SSRs with high polymorphism, 2034 SSRs of all 141,895 loci showing polymorphism among the 6 resequenced peach accessions were retained. Next, 1334 (65.6%) mononucleotide repeats were excluded because the loci may have resulted in weak PCR products. Finally, the loci that were detected in more than five accessions were identified for subsequent analysis.

The remaining 194 SSRs were used to design primers to test their polymorphism in 21 peach accessions. However, the primer design of 7 SSRs failed due to the high sequence similarity of the flanking sequence of these SSRs with multiple regions in the genome. In addition, a total of 23 primer pairs generated unfavorable amplification products. Thus, 164 SSR primer pairs (Additional file 1: Table S3) with clear amplification banding patterns were retained, and their genotype is shown in Additional file 1: Table S4.

To assess the application of these SSRs, 15 polymorphic markers with more than 7 alleles when amplified from 21 peaches were randomly selected to analyze the genetic relationship among 221 accessions (Additional file 1: Table S5). These SSR markers detected a total of 210 alleles falling within a range of 8–26 alleles per locus (Additional file 1: Table S6). Among all alleles, 36 were specific to 28 peach accessions. Shannon’s information index (I) ranged from 0.0161 to 0.6896, with 101 alleles producing I values > 0.1. The I values of seven SSR markers (SSR073, 093, 120, 169, 179, 183, and 184) were higher than 0.2, indicating high efficiency.

**Fig. 6:**
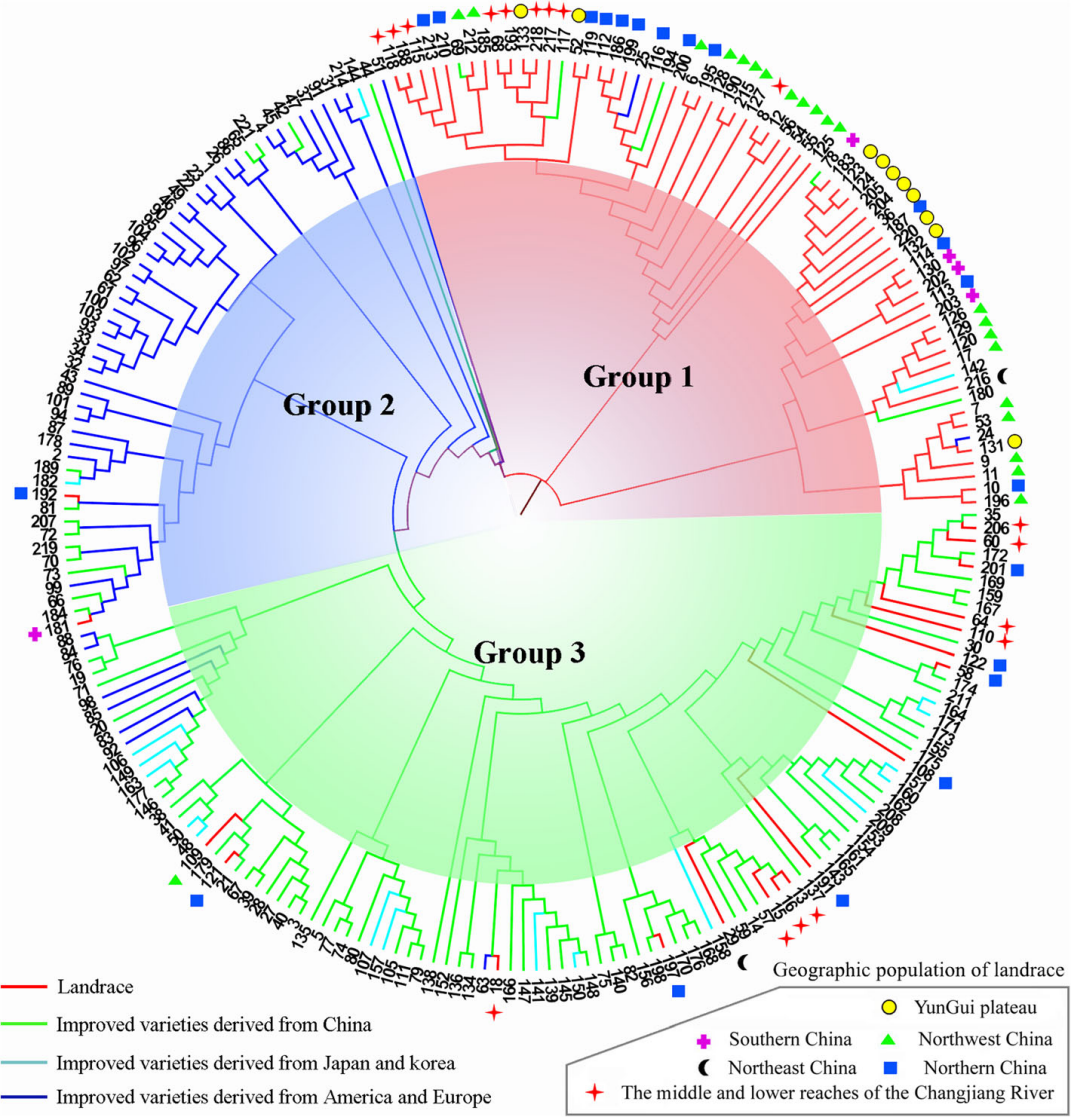
The phylogenetic tree of 221 peach accessions calculated by MEGA software using the genotypes of 15 SSR markers. The red line indicates landraces; the green line indicates improved varieties derived from China; the cyan line indicates improved varieties derived from Japan and Korea; the blue line indicates improved varieties derived from America and Europe

We constructed a neighbor-joining phylogenetic tree based on the genetic distances calculated from the genotypes at all 15 SSR positions of the 221 peach accession, and three clusters were delineated (Fig. 6). Group 1 contained 65 accessions, among which 75% (57 of 56) were landraces, mostly from northwest China (21 of 22), northern China (13 of 21), YunGui Plateau(10 of 10) and southern China (4 0f 5). Group 2 contained 54 accessions, including mostly improved varieties derived from America and Europe (37 of 44), 13 improved varieties derived from China, 2 improved varieties derived from Japan and Korea and 2 landraces. The improved varieties from China (59 of 78) and Japan and Korea (19 of 23) were mainly clustered into group 3, probably because they belong to Asian peach genotypes. We found that some landraces originating from the middle and low reaches of the Changjiang River (8) and northern China (7) were closely related with the above improved varieties.

To compare the levels of polymorphism between the SSR markers developed in this study and those previously reported, we selected 15 previously reported SSRs with high polymorphism from 36 accessions. Comparing the two studies using the same accessions (Additional file 1: Table S7), these two groups of SSR markers were polymorphic and produced a total of 171 and 153 alleles. The average number of alleles per locus for the 15 SSR markers developed in this study was 11.4, ranging from 6 (SSR73) to 22 (SSR152), which was slightly higher than that of the previously developed SSR markers (10.2). Importantly, there was no obvious difference between the two groups in molecular diversity, such as the I and G_D_ values (Additional file 1: Figure S4).

## Discussion

### Genomic distribution of variation

In the study, we identified 44,245 SVs, 12,302 CNVs, and 138,476 SSRs across 8 chromosomes in peach with high efficiency via whole-genome sequencing (Fig. 2). These variations with long segments can be easily converted into DNA markers and will be useful in genetic and functional genomic studies [16]. We found that the density of SVs (202.71 per Mb) was lower than that in our previous report (836 SVs/Mb) [15] using 84 *Prunus* accessions. The reason for this finding may be related to the narrow genetic background of the accessions used in this study (all belonging to *P. persica*) compared with those of the previous study, which included different species, such as *P. persica*, *P. ferganensis*, *P. kansuensis*, *P. davidiana*, and *P. mira*.

Interestingly, SVs were the main element affecting the function of genes associated with key agronomic characteristics in peach, such as flesh color [21] and fruit hairiness [22]. Therefore, the discovery and distribution analysis of SVs will accelerate our rapid identification of candidate genes within the previous location using linkage analysis and GWAS. Furthermore, the density of SSRs (634.43 per Mb) identified in this study was higher than that in cucumber (552 SSR/Mb) [23], watermelon (111 SSR/Mb) [8], and most species of *Triticeae* (96~668 SSRs/Mb) [24]. The abundance of SSRs in peach can facilitate the genetic research of this species.

### The nucleotide repeats of SSRs

Frequency analysis of various nucleotide repeats of SSRs in the peach genome revealed that mononucleotides were the most abundant SSR (47.36% of the total), followed by di-, tri-, etc. (Table 4). However, tetranucleotide repeats were the primary motif in cucumber [23], Chinese jujube [18] and pear [25] and the most abundant in soybean [26] and rice [27]. Among the dinucleotide repeats in peach, AT/TA was the most common, followed by CT/TC (Table 4). This result is different from that in mei [12] and watermelon [8], in which the AG/CT motif was most abundant among dinucleotide repeats. This result is also different from that in the sequences in peach, in which CT/TC was the most abundant dinucleotide repeat [18]. GC/CG repeats, while rare in the peach genome, seem to be common in the rice [27], mei [12], and Chinese jujube [18] genomes.

In this study, we found that there were some regions abundant in SSRs, such as 17 to 17.1 Mb of Chr. 2 and 18.2 to 18.3 Mb of Chr. 5. However, in the other regions, such as 7.1 to 7.6 Mb of Chr. 5, the SSRs were limited. The uneven distribution of SSRs in the peach genome has also been identified in other species, such as cotton [28] and watermelon [8]. This result is helpful for analyzing the differences in chromosomes during genome evolution.

### Polymorphism of SSRs developed in this study and its application

Before this study, no more than 300 SSRs were publicly available for peach on the GDR website. Some studies have been performed to screen the SSRs in peach at the genome-wide level. For example, Chen et al. [29] identified 288 SSRs with optimized distribution and reliability for genotype evaluation in peach using expressed sequence tags. Wang et al. [16] identified a total of 17,979 SSRs in the peach transcriptome sequenced from leaf, flower, and fruit tissues. In this study, 141,895 SSR loci were identified using resequencing data from six peach accessions. Among these SSRs, the di- and trinucleotide repeats should be the focus of further studies because they are abundant and their PCR products are easily distinguished using electrophoresis. Through the screening of these SSR markers, 187 were selected by amplification in 21 peach accessions and validation in 221 accessions. These markers showed a high polymorphism similar to that of the SSRs identified previously. After comparing the location of SSRs developed in the study and previous report in peach genome, we found no same location of those loci at all. Therefore, the former SSR set will be a useful complement to the current SSR panel.

Previously, many studies have analyzed the evolution of peach among different species [30, 31]; however, the divergence among different geographic populations of landraces has not been well studied to date. Using 15 polymorphic markers and 221 peach accessions, we performed a phylogenetic analysis. According to the results, we first determined that the landraces originating from the middle and low reaches of the Changjiang River have a distant genetic relationship with other landraces in China. This finding is similar to a previous study from our laboratory, which demonstrated that the middle and low reaches of the Changjiang River had the highest genetic diversity among six populations and should hence be presumed as the landrace origin [7, 32] and an archeological study also proved it [33]. However, it is different from other studies that suggested Northwest China [34, 35], the YGC [34], and NC [36] were the origin centers of landraces. Second, the close relationship between the improved varieties derived from China and Japan may be related to their developing from the same ancestor, ‘Chinese Cling’ (Accession No. 110). This conclusion has been reported several times [37, 38], which suggests that most modern peach cultivars were selected from seedlings of ‘Chinese Cling’. Third, occidental peaches were introduced from China and may be the offspring of landraces originating from Northwest China. This result confirmed that there are numerous germplasm resources of peaches and nectarines in the world due to their introduction into Persia and other countries as early as approximately 200 BCE along the Silk Road from China [39]. The similar clustering result with the previous report indicates that these SSRs are available in genetic studies.

NGS technologies are a high-efficiency tool for identifying a large number of polymorphic DNA markers for genetic research. The abundance of DNA markers developed in this study may potentially be applied in the germplasm conservation, gene identification, and molecular breeding of peach and can be transferred to other members of the Rosaceae family.

## Conclusions

The results showed that the use of high-throughput sequencing to develop DNA markers is fast and effective. Comprehensive identification of DNA markers, including SVs and SSRs, would be of benefit to genetic diversity evaluation, genetic mapping, and molecular breeding of peach.

## Methods

### Plant materials

All materials were sampled from the National Fruit Tree Germplasm Repository, Zhengzhou Fruit Research Institute, Chinese Academy of Agricultural Sciences, Henan Province, PRC. Six distantly related peach accessions, ‘Ka Shi Huang Rou Li Guang’, ‘Jin Mi Xia Ye Tao’, ‘Xin Jiang Pan Tao 2’, ‘Xia Miao 1’, ‘Sa Hua Hong Pan Tao’, and ‘Wu Yue Xian Bian Gan’ [15], were collected for sequencing to identify DNA markers. Subsequently, 221 accessions (Additional file 1: Table S1) were used to perform association study of fruit maturity date time and analyze their genetic relationship. Finally, 36 samples were chosen to compare the polymorphism differences between the SSRs developed in this study and those previously reported. To enhance the diversity and representativeness, we applied the following rules to our selection of the samples: (1) each sample had an independent local name; (2) accessions from different ecotypes of landraces (Northwest China, Northeast China, the Yun Gui Plateau (YGC), the middle and lower reaches of the Yangtze River, a wide range of northern China, and southern China) were selected; and (3) improved varieties were also included to broaden the diversity of edible peach.

### DNA extraction

Young leaves were collected and transported to the laboratory on ice and stored at − 80 °C. All DNA samples were extracted using a plant genomic DNA extraction kit (Tiangen, Beijing, China). DNA purification was performed using RNase I (Takara, Dalian, China) before sequencing. DNA quality and concentration were measured with a NanoDrop 2000 UV spectrophotometer (Thermo Fisher Scientific, Waltham, USA). The DNA concentrations for sequencing and PCR amplification were diluted to 200 ng/μL and 20 ng/μL, respectively.

### Sequence and variation detection

The genome was sequenced using the Illumina 2500 platform (Illumina, San Diego, CA, USA) with a 150 bp pair-end. The adaptor and low-quality sequences were filtered, and all clean reads were aligned to the peach reference genome v1.0 [30] using BWA v 0.7.12 [40], with a cutoff maximum of three mismatches in 150 bp. SNPs and small InDels were detected by GATK 4.0 [41], SVs and CNVs were identified with the BreakDancer software package [42] and SSRs were screened with Msatfinder software [43]. Variations annotation were performed based on its corresponding location in peach reference genome version a 2.1 and its homolog function annotation result.

### Fruit maturity date investigation

Fruit maturity date of 221 peach accessions (Additional file 1: Table S1) was investigated based on previously published plant genetic resources evaluation criteria [44] in two successive years, from 2011 and 2012. The fruit maturity date was recorded from May 24th until September 9th.

### Gene ontology (GO) enrichment analysis

GO enrichment analysis of differentially expressed genes (DEGs) was carried out using WEGO [45].

### Tissue-specific expression pattern of the genes containing SSRs

Total RNA was extracted from different tissues, including leaves, flowers, fruit, roots, phloem, and seeds, of *P. persica* ‘08–9-107’ using an RNA Extraction kit (Aidlab, Beijing, China), following the manufacturer’s protocol. The method for mRNA enrichment, double-stranded cDNA synthesis, digestion, PCR amplification, cDNA library construction, RNA sequencing, data filtering, and the number of fragments per kilobase of exon per million fragments (FPKM) calculation were previously described by Cao et al. [46].

### Association study

To demonstrate the application of different variations, we obtained SNPs from 9.6 Mb to 17.1 Mb on Chromosome (Chr.) 4 according to the previsous QTLs of fruit ripening time [20], then filtered SNPs with missing rate < 0.2 and minor allele frequency (MAF) < 0.05, selected about 1000 SNP with the highest sequencing depth for association study. The genotypes of the selected SNPs were recorded using the Sequenom MassARRAY platform in 221 accessions. A mixed linear model (MLM) program, Efficient Mixed-Model Association eXpedited (EMMAX) [47] (version beta), was used to carry out the association analyses. To minimize false positives, kinship estimated with the EMMAX emmax-kin program was taken into account to perform association analysis [47]. We defined the associated statistical significance cutoff as the Bonferroni test threshold, which was set as 0.01/total SNPs (−log_10_*P* = 4.97 for fruit maturity).

### SSR primer design and synthesis

The primer pairs were designed using Primer 5.0 (Primer-E Ltd., Plymouth, UK) with the following settings: primer length between 18 and 27 bp, GC content of 40–60%, optimum annealing temperature of at least 50 °C, and PCR product size ranging from 100 to 500 bp. M13 sequences were added to the 5′ end of all the primers (Genewiz, Suzhou, China). The M13 primers were used to amplify 187 SSR loci in 21 peach accessions. Fifteen fluorescent primer pairs were synthesized using Oligo 192 to analyze genetic relationships among 221 peach accessions.

### PCR amplification

PCR amplification with M13 primers was performed with a GeneAmp PCR System 9700 (Applied Biosystems, Carlsbad, CA). The reaction mixture had a total volume of 10 μL and contained 20 ng of template genomic DNA, 0.8 μL of 2.5 μM dNTP, 0.6 μL of each primer at 100 μM, 1 μL of 10× buffer, 0.1 μL of 5 U/μL Taq polymerase, 0.6 μL of 5 μM HEX and 6 μL of sterile water. The PCR conditions were 95 °C for 5 min; 30 cycles of 30 s at 95 °C, 30 s at the annealing temperature of each primer pair at 56 °C, and 30 s at 72 °C; 10 cycles of 30 s at 95 °C, 30 s at 53 °C, and 30 s at 72 °C; and a final step at 60 °C for 30 min. The PCR amplification conditions for fluorescent primers were the same as those described above, but without 0.6 μL of 5 μM HEX in the 10 μL reaction mixture.

### Detection of SSR polymorphism and estimation of allele sizes

The polymorphism of the SSR markers was detected using a 3730xl DNA Analyzer (Thermo Fisher Scientific, Waltham, USA). Denaturation conditions were 95 °C for 5 min. The samples were prepared by mixing 1.0 μL of PCR products with 8.5 μL deionized formamide and 0.5 μL ROX-500. The SSR fragment sizes were analyzed with GeneMapper 5.0 [48].

### Screening of the previously developed SSRs

We consulted the literature published in the past few decades and selected 15 highly polymorphic SSR loci based on allele number among different peach varieties. Among them, nine (BPPCT008, CPPCT022, BPPCT017, UDP008, BPPCT020, UDP40, BPPCT034, CPPCT005, and UDP409) were selected from the published report by Chen et al. [49], five (CPPCT031, UDP001, BPPCT009, CPPCT003, and CPPCT013) were selected from the published study by Yoon et al. [39], and one (BPPCT015) was selected from the published study by Aranzana et al. [31]. These markers were selected to compare their polymorphism with the markers developed in this study.

### Genetic diversity and phylogenetic analyses

The molecular diversity parameters, such as the number of alleles per locus (N_A_), number of private alleles (N_PA_), Shannon’s information index (I), and genetic distance (G_D_), were all estimated with POPGENE v1.31 [50].

The software MEGA 5.0 was used to calculate the clustering tree based on the 15 SSRs of 221 accessions. The algorithm we chose used the neighbor-joining (NJ) method. We performed statistical test using bootstrap test, and the No. of bootstrap replication was set to 1000.

## Supplementary information

**Additional file 1: Figure S1.** The statistics of synonymous and nonsynonymous SNPs (a) and large-effect SNPs (b), which could affect the gene function in different peach accessions. **Figure S2**. GO annotation of genes containing large-effect SNPs. The GO results are summarized in three main categories: biological process, cellular compartment, and molecular function. **Figure S3.** The NA, NPA, I, and GD index of genotyping in 36 peach accessions using SSR markers developed in the study and reported previously. NA indicates the number of alleles per locus; NPA indicates the number of private alleles; I indicates Shannon’s information index; and GD indicates gene diversity. **Figure S4.** The N_A_, N_PA_, I, and G_D_ index of genotyping in 36 peach accessions using SSR markers developed in the study and reported previously. **Table S1.** A total of 221 peach accessions were collected to evaluate genetic diversity and perform association analysis. **Table S2.** The number and percent of different nucleotide repeats located in CDS and UTRs. **Table S3.** The location of 164 SSRs and their primer sequences designed in this study. **Table S4.** Polymorphism and allele number estimation of PCR product amplified with 164 SSRs from 21 peach accessions. **Table S5.** Polymorphism of 15 SSR markers amplified from 221 peach accessions. **Table S6.** Number of alleles per locus and diversity index detected by 15 polymorphic SSRs among 221 peach accessions. **Table S7.** Polymorphism comparisons among 36 accessions amplified using SSR markers developed in this study and reported previously.

## Data Availability

The datasets used and/or analysed during the current study are available from the corresponding author on reasonable request. The high-depth sequencing data of six peach accessions have been deposited in the Sequence Read Archive (SRA) of National Center for Biotechnology Information with accession numbers SRR10254991, SRR10254990, SRR10254993, SRR10254992, SRX4994112 and SRX4994108.

## Abbreviations

CNV: Copy number variant
DEG: Differentially expressed gene
EMMAX: Efficient Mixed-Model Association eXpedited
FPKM: Fragments per kilobase of exon per million fragments
GD: Genetic distance
GWAS: Genome-wide association study
I: Shannon’s information index
InDel: Insertion/deletion
MAF: Minor allele frequency
MLM: Mixed linear model
NA: Number of alleles per locus
NGS: Next-generation sequencin
NJ: Neighbor-joining
NPA: Number of private alleles
SNP: Single nucleotide polymorphism
SSR: Simple sequence repea
SV: Structure variant
YGC: Yun Gui Plateau
