# Supplementary material for: Detection and application of genome-wide variations in peach for association and genetic relationship analysis
Source: BMC Genet. 2019 Dec 30;20:101. doi: 10.1186/s12863-019-0799-8 (PMC6937647; doi:10.1186/s12863-019-0799-8)
Supplement: Supplementary file 1 — Additional file 1: Figure S1. The statistics of synonymous and nonsynonymous SNPs (a) and large-effect SNPs (b), which could affect the gene function in different peach accessions. Figure S2. GO annotation of genes containing large-effect SNPs. The GO results are summarized in three main categories: biological process, cellular compartment, and molecular function. Figure S3. The NA, NPA, I, and GD index of genotyping in 36 peach accessions using SSR markers developed in the study and reported previously. NA indicates the number of alleles per locus; NPA indicates the number of private alleles; I indicates Shannon’s information index; and GD indicates gene diversity. Figure S4. The NA, NPA, I, and GD index of genotyping in 36 peach accessions using SSR markers developed in the study and reported previously. Table S1. A total of 221 peach accessions were collected to evaluate genetic diversity and perform association analysis. Table S2. The number and percent of different nucleotide repeats located in CDS and UTRs. Table S3. The location of 164 SSRs and their primer sequences designed in this study. Table S4. Polymorphism and allele number estimation of PCR product amplified with 164 SSRs from 21 peach accessions. Table S5. Polymorphism of 15 SSR markers amplified from 221 peach accessions. Table S6. Number of alleles per locus and diversity index detected by 15 polymorphic SSRs among 221 peach accessions. Table S7. Polymorphism comparisons among 36 accessions amplified using SSR markers developed in this study and reported previously. [file 12863_2019_799_MOESM1_ESM.docx]

**Additional files**

**S1 Fig. The statistics of synonymous and nonsynonymous SNPs (a) and large-effect SNPs (b) which could affect the gene function in different peach accessions.**

|  |  |
| --- | --- |

**S2 Fig. Gene ontology (GO) annotation of genes contained large-effect SNPs.** The GO results are summarized in three main categories: biological process, cellular compartment, and molecular function.

**
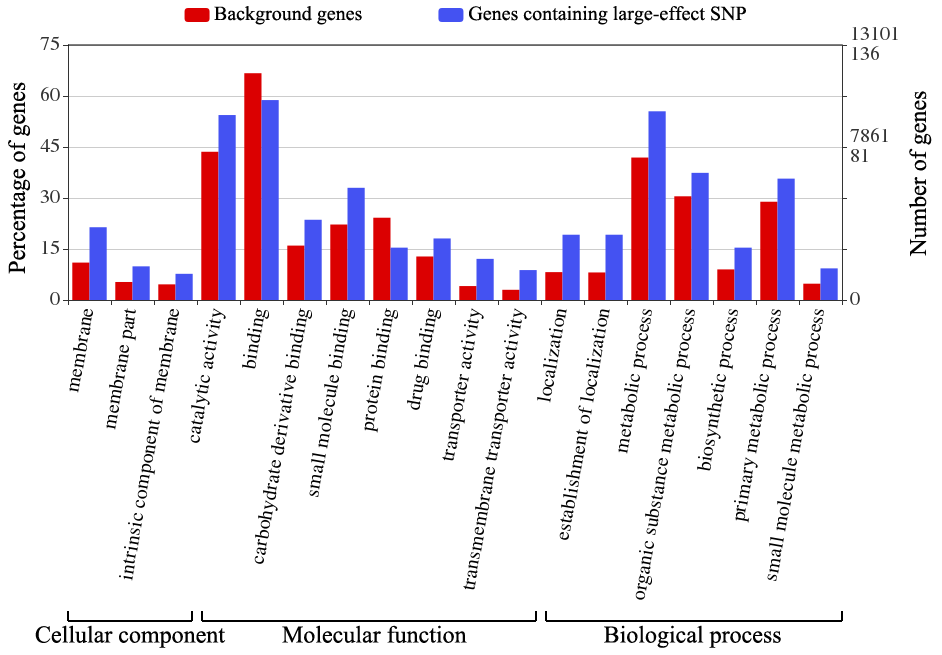
**

**S3 Fig. Tissue-specific expression of genes contained the large-effect SNPs of ‘Chinese Cling’ peach in root, fruit, phloem, leaf and seed.**

**
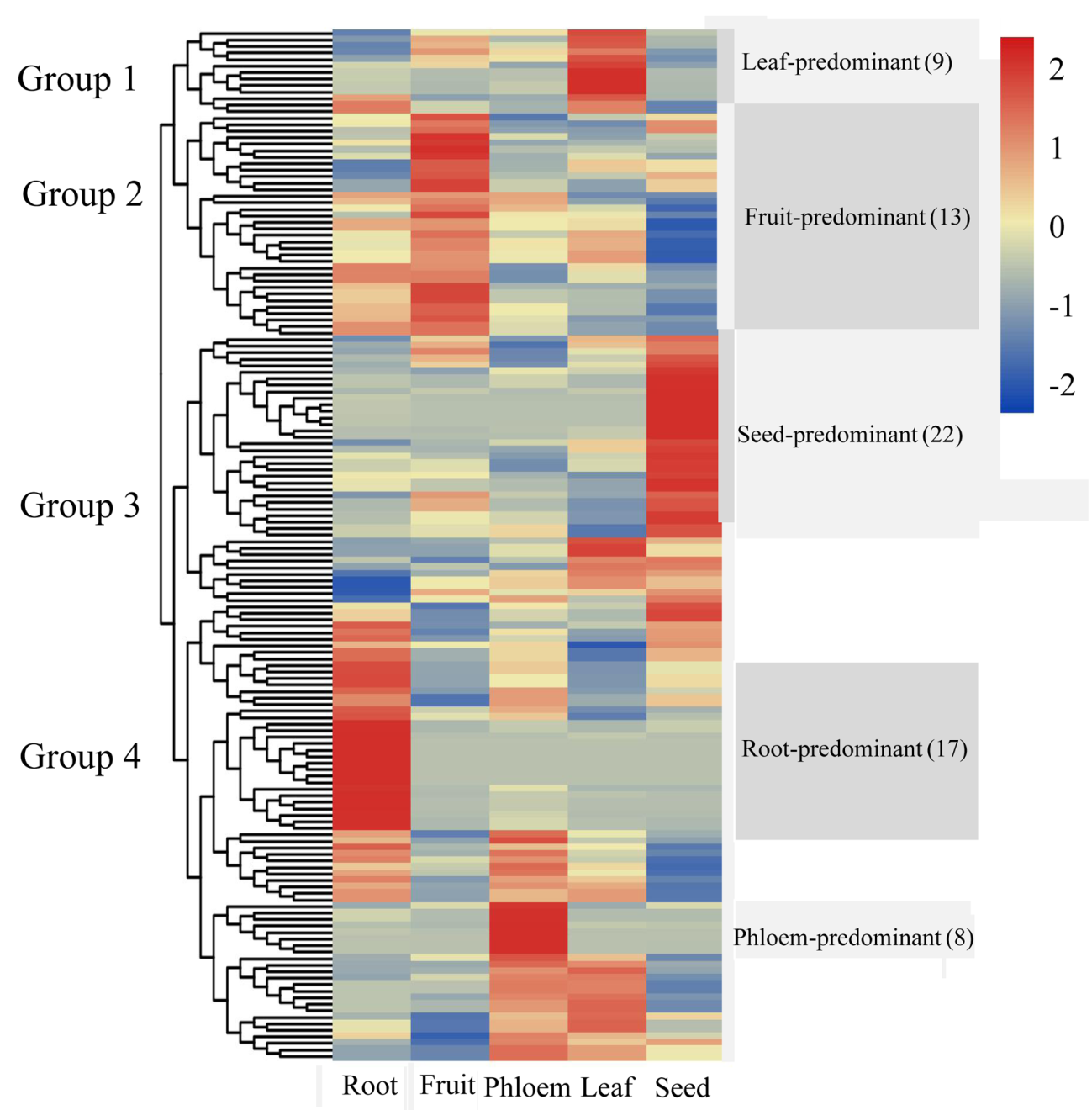
**

**S4 Fig. The N_A_, N_PA_, I, and G_D_ index of genotyping in 36 peach accessions using SSR markers developed in the study and reported previously.** N_A_ indicates the number of alleles per locus; N_PA_, the number of private alleles; I, Shannon’s information index; G_D_, gene diversity.

**
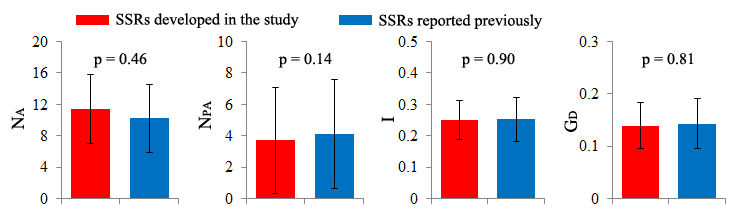
**

**S1 Tab. A total of 221 peach accessions collected to evaluate genetic diversity and perform association analysis.**

| **Accession no.** | **Name** | **Origin** | **Category** | **Accession no.** | **Name** | **Origin** | **Category** | **Accession no.** | **Name** | **Origin** | **Category** |
| --- | --- | --- | --- | --- | --- | --- | --- | --- | --- | --- | --- |
| 1 | Harmony | America | Improved variety | 76 | Zhong You 8 | Henan,China | Improved variety | 151 | Hatsukami* | Zhengzhou,China | Improved variety |
| 2 | Flordaking | America | Improved variety | 77 | Rui Guang 5 | Beijing,China | Improved variety | 152 | Hatsukami | Nanjing,China | Improved variety |
| 3 | Feng Huang* | Liaoning,China | Improved variety | 78 | Shuang Xi Hong* | Henan,China | Improved variety | 153 | Zhao Hui | Jiangsu,China | Improved variety |
| 4 | Halford | America | Improved variety | 79 | Qin Guang 2 | Shaanxi,China | Improved variety | 154 | Hui Yu Lu | Jiangsu,China | Improved variety |
| 5 | Rui Guang 3* | Beijing,China | Improved variety | 80 | Rui Guang 7 | Beijing,China | Improved variety | 155 | Jie Tu Bai | Hebei,China | Improved variety |
| 6 | Zao Shu Huang Gan | Gansu,China | Landrace | 81 | Zi Rou Tao | Henan,China | Improved variety | 156 | Xi Nong Shui Mi | Shaanxi,China | Improved variety |
| 7 | Hetian Huang Rou | Xinjiang,China | Landrace | 82 | Hong Shan Hu | Beijing,China | Improved variety | 157 | Jing Yu* | Beijing,China | Improved variety |
| 8 | Jiangcun 1 | Anhui,China | Landrace | 83 | Hu You 004 | Shanghai,China | Improved variety | 158 | Azumo | Japan | Improved variety |
| 9 | Da Li He Huang Rou | Xinjiang,China | Landrace | 84 | NJN76* | America | Improved variety | 159 | Yu Lu | Zhejiang,China | Improved variety |
| 10 | Yexiang Huang Rou Tao | Henan,China | Landrace | 85 | May Fire* | America | Improved variety | 160 | Hakuho* | Japan | Improved variety |
| 11 | Mo Yu 8 | Xinjiang,China | Landrace | 86 | NJN78 | America | Improved variety | 161 | Kawanakajima Hakuto | Japan | Improved variety |
| 12 | Long 1-2-4 | Gansu,China | Landrace | 87 | NJN72 | America | Improved variety | 162 | Asama Hakuto | Japan | Improved variety |
| 13 | Bai Mang Pan Tao | Shanghai,China | Landrace | 88 | NJN80 | America | Improved variety | 163 | Benishimizi | Japan | Improved variety |
| 14 | Zao Lu Pan Tao | Beijing,China | Improved variety | 89 | Nectagrand 2 | Italy | Improved variety | 164 | Okubo* | Japan | Improved variety |
| 15 | Fenghua Pan Tao* | Zhejiang,China | Landrace | 90 | May Grand | America | Improved variety | 165 | Kouyou Hakuto | Japan | Improved variety |
| 16 | Yu Lu Pan Tao | Zhejiang,China | Landrace | 91 | NJN89 | America | Improved variety | 166 | Zao Shanghai Shui Mi | Shanghai,China | Improved variety |
| 17 | Rou Pan Tao | Gansu,China | Landrace | 92 | Sunred | America | Improved variety | 167 | Qiu Xiang Mi | Jiangsu,China | Improved variety |
| 18 | Li He Pan Tao | Zhejiang,China | Landrace | 93 | Legrand | America | Improved variety | 168 | Xin Bai Hua | Jiangsu,China | Improved variety |
| 19 | Yan Guang | Henan,China | Improved variety | 94 | Nectared 7 | Italy | Improved variety | 169 | Bai Hua* | Jiangsu,China | Improved variety |
| 20 | Zhong You 5 | Henan,China | Improved variety | 95 | Flavortop | America | Improved variety | 170 | Wan Bai | Sichuan,China | Improved variety |
| 21 | Chun Guan | Gansu,China | Improved variety | 96 | Fantasia | America | Improved variety | 171 | Yumyeong | Korea | Improved variety |
| 22 | Favolate 2 | Italy | Improved variety | 97 | Sungold | America | Improved variety | 172 | Ying Qing | Jiangsu,China | Improved variety |
| 23 | Fertilia Morettimi | Italy | Improved variety | 98 | Armking | America | Improved variety | 173 | Jing Yan | Beijing,China | Improved variety |
| 24 | Dikired | America | Improved variety | 99 | Early red 2* | America | Improved variety | 174 | Lai Shan Mi | Shandong,China | Improved variety |
| 25 | Goldcrest | America | Improved variety | 100 | Summer Beauty | America | Improved variety | 175 | Qin Wang | Shaanxi,China | Improved variety |
| 26 | Redhaven | America | Improved variety | 101 | Super Crimson | America | Improved variety | 176 | Qiu Xiang | Beijing,China | Improved variety |
| 27 | Cheng Xiang | Liaoning,China | Improved variety | 102 | Red Diamond | America | Improved variety | 177 | Feng Bai | Liaoning,China | Improved variety |
| 28 | Lu Xiang | Liaoning,China | Improved variety | 103 | Redgold | America | Improved variety | 178 | Maravilha | America | Improved variety |
| 29 | Zao Huang Jin | Liaoning,China | Landrace | 104 | Hardrided | Canada | Improved variety | 179 | 21 shiji | Hebei,China | Improved variety |
| 30 | Wan Huang Jin | Japan | Improved variety | 105 | Okitsu* | Japan | Improved variety | 180 | Zhong Hua Shou Tao | Shandong,China | Improved variety |
| 31 | Reliance | America | Improved variety | 106 | Shuho | Japan | Improved variety | 181 | Ying Ge Tao | Taiwan, China | Landrace |
| 32 | Fay Elberta | America | Improved variety | 107 | Imoiouniku | Japan | Improved variety | 182 | Tokyo Red | Japan | Improved variety |
| 33 | Havis | America | Improved variety | 108 | Da Hong Tao | Shanxi,China | Landrace | 183 | Nanshan Tian Tao | Guangdpmg,China | Landrace |
| 34 | Little Folwer Elberta* | America | Improved variety | 109 | Tai Bai | Shaanxi,China | Landrace | 184 | Tai Nong 2 | Taiwan,China | Improved variety |
| 35 | Jin Xiu | Shanghai,China | Improved variety | 110 | Chinese Cling* | Shanghai,China | Landrace | 185 | Bai Sha | Shaanxi,China | Landrace |
| 36 | Huang Nian Hu | Yunnan,China | Landrace | 111 | Jing Mi | Beijing,China | Improved variety | 186 | Liu Yue Bai | Hebei,China | Landrace |
| 37 | Spring baby* | America | Improved variety | 112 | Wu yue Xian | Beijing,China | Landrace | 187 | Yi Xian Hong | Hebei,China | Landrace |
| 38 | Zheng Huang 2 | Henan,China | Improved variety | 113 | Da Guo Hei Tao | Shandong,China | Landrace | 188 | Wuhan 2 | Hubei,China | Landrace |
| 39 | Zheng Huang 3 | Henan,China | Improved variety | 114 | Tianjin Shui Mi* | Tianjin,China | Landrace | 189 | Datuan Mi Lu | Shanghai,China | Improved variety |
| 40 | Zao Huang Guan | Shaanxi,China | Improved variety | 115 | Wu Hei Ji Rou Tao | Anhui,China | Landrace | 190 | Qi Tao | Gansu,China | Landrace |
| 41 | Dalian 12-28 | Dalian,China | Improved variety | 116 | Ge Gu | Hebei,China | Landrace | 191 | Licheng Tao | Shandong,China | Landrace |
| 42 | Dalian 22-8 | Dalian,China | Improved variety | 117 | Diao Zhi Bai | Anhui,China | Improved variety | 192 | Shenzhou Li He Shui Mi | Hebei,China | Landrace |
| 43 | NJC105 | America | Improved variety | 118 | Da Hong Pao* | Hubei,China | Landrace | 193 | Wenzhou Shui Mi | Zhejiang,China | Landrace |
| 44 | Dalian 22-6 | Dalian,China | Improved variety | 119 | Qingzhou Hong Pi Mi Tao | Shandong,China | Landrace | 194 | Yu Bai* | Henan,China | Improved variety |
| 45 | Babygold 6 | America | Improved variety | 120 | Gaotai 1 | Gansu,China | Landrace | 195 | Yangquan Rou Tao | Shanxi,China | Landrace |
| 46 | NJC3 | America | Improved variety | 121 | Da Xue Tao | Hebei,China | Landrace | 196 | Mi Yang Shan | Xinjiang,China | Landrace |
| 47 | NJC47 | America | Improved variety | 122 | Han Lu Mi | Shandong,China | Landrace | 197 | Ying Xue | Beijing,China | Landrace |
| 48 | Nishiki | Japan | Improved variety | 123 | Bai Nian Hu | Yunnan,China | Landrace | 198 | Xi Mei 1 | Shaanxi,China | Improved variety |
| 49 | Kanto 14 | Japan | Improved variety | 124 | Bai Li Hu | Yunnan,China | Landrace | 199 | Feicheng Hong Li 6 | Shandong,China | Landrace |
| 50 | Myojo | Japan | Improved variety | 125 | Xijiao 3 | Shaanxi,China | Landrace | 200 | Suan Tao | Shandong,China | Landrace |
| 51 | sims | America | Improved variety | 126 | Zhang Bai 2 | Gansu,China | Landrace | 201 | Yuan Chun Bai | Hebei,China | Landrace |
| 52 | Xiao Jin Dan | Yunnan,China | Landrace | 127 | Zhang Bai 5 | Gansu,China | Landrace | 202 | Shi Tao | Guangxi,China | Landrace |
| 53 | Yili Xian Huang Rou | Xinjiang,China | Landrace | 128 | Zhang Bai Gan | Gansu,China | Landrace | 203 | Gua Tao | Guangxi,China | Landrace |
| 54 | Long 2-4-6 | Gansu,China | Landrace | 129 | Lin Bai 10 | Gansu,China | Landrace | 204 | Qiu Bai Tao | Yunnan,China | Landrace |
| 55 | Long 1-2-6 | Gansu,China | Landrace | 130 | Xian Tao | Guangxi,China | Landrace | 205 | Er Zao Tao | Yunnan,China | Landrace |
| 56 | Xizhuang 1 | Gansu,China | Landrace | 131 | Anlong Bai Tao | Guizhou,China | Landrace | 206 | Qing Mao Zi Bai Hua | Sichuan,China | Landrace |
| 57 | Zao Huang Pan Tao | Henan,China | Improved variety | 132 | Jiuyang Qing Tao | Guizhou,China | Landrace | 207 | Jin Feng | Henan,China | Improved variety |
| 58 | Wu Yue Xian Bian Gan | Beijing,China | Landrace | 133 | Wangmo Xiao Mi Tao | Guizhou,China | Landrace | 208 | Xinong Xin Mi | Shaanxi,China | Improved variety |
| 59 | Xin Hong Zao Pan Tao | Shaanxi,China | Improved variety | 134 | Chun Lei | Shanghai,China | Improved variety | 209 | Reddomum | Japan | Improved variety |
| 60 | Jiaqing Pan Tao | Zhejiang,China | Landrace | 135 | Zao Mei | Shanghai,China | Improved variety | 210 | Er Jie Bai | Henan,China | Landrace |
| 61 | Stark Saturn | America | Improved variety | 136 | Chun Hua | Shanghai,China | Improved variety | 211 | Yu Tian | Henan,China | Improved variety |
| 62 | NJF7 | America | Improved variety | 137 | Xia Hui 1 | Jiangsu,China | Improved variety | 212 | Kashi Huang Rou Li Guang | Xinjiang,China | Landrace |
| 63 | Su Lian Pan Tao | Former Soviet Union | Improved variety | 138 | Jing Chun | Beijing,China | Improved variety | 213 | Jian Zui Hong Rou | Henan,China | Landrace |
| 64 | Wan Pan Tao | Zhejiang,China | Landrace | 139 | Yu Hua Lu | Jiangsu,China | Improved variety | 214 | Harrow blood | Canada | Improved variety |
| 65 | Zhong You Pan 2 | Henan,China | Improved variety | 140 | Mang Xia Lu | Jiangsu,China | Improved variety | 215 | Tian Li Guang | Xinjiang,China | Landrace |
| 66 | Long You Pan Tao | Henan,China | Improved variety | 141 | Sunago Wase | Japan | Improved variety | 216 | Hui Chun Tao | Jilin,China | Landrace |
| 67 | Zhong You Pan 3 | Henan,China | Improved variety | 142 | Tsukuba 85 | Japan | Improved variety | 217 | Su Hong | Jiangsu,China | Landrace |
| 68 | Wanzhou Suan Tao | Chongqing,China | Landrace | 143 | Zao Bai Feng | Shanghai,China | Improved variety | 218 | Huo Zhu | Jiangsu,China | Landrace |
| 69 | Bi Xia Pan Tao | Beijing,China | Improved variety | 144 | Toobo | Japan | Improved variety | 219 | Shu Guang Variation | Henan,China | Improved variety |
| 70 | Shu Guang* | Henan,China | Improved variety | 145 | Weiyang 2 | Shaanxi,China | Improved variety | 220 | Feijing Tao | Yunnan, China | Landrace |
| 71 | Hua Guang | Henan,China | Improved variety | 146 | Jin Hui | Henan,China | Improved variety | 221 | Zhen 1-3 Pan Tao* | Henan,China | Improved variety |
| 72 | Qian Nian Hong | Henan,China | Improved variety | 147 | Zao Yan | Beijing,China | Improved variety |  |  |  |  |
| 73 | Dan Mo | Beijing,China | Improved variety | 148 | Sha Hong Tao | Shaanxi,China | Improved variety |  |  |  |  |
| 74 | Rui Guang 3 | Beijing,China | Improved variety | 149 | Matsumori | Japan | Improved variety |  |  |  |  |
| 75 | Mei Gui Hong | Henan,China | Improved variety | 150 | Kurakato Wase | Japan | Improved variety |  |  |  |  |

Note: asterisk indicate the 21 peach accessions for screening high polymorphism SSR markers.

**S2 Tab. The number and percent of different nucleodite repeats which located in CDS and UTR.**

| Motif | CDS | | UTR | |
| --- | --- | --- | --- | --- |
|  | Number | Percent (%) | Number | Percent (%) |
| Mono-nucleotide | 4349 | 51.70 | 621 | 43.43 |
| Di-nucleotide | 2378 | 28.27 | 663 | 46.36 |
| Tri-nucleotide | 1297 | 15.42 | 85 | 5.94 |
| Tetra-nucleotide | 67 | 0.80 | 12 | 0.84 |
| Penta-nucleotide | 106 | 1.26 | 31 | 2.17 |
| Hexa-nucleotide | 215 | 2.56 | 18 | 1.26 |

**S3 Tab. The location of 164 SSRs and their primer sequence designed in the study.**

| SSR accession | SSR location | SSR motif | Primer sequence (5'-3') | PCR product (bp) |
| --- | --- | --- | --- | --- |
| SSR001 | Chr. 5:15867183-15867200 | (AT)9 | F: AATTTATTGCATTATTTTCCGCA | 145 |
|  |  |  | R: CAAGGCTTACAACTCCTCAAGTG |  |
| SSR002 | Chr. 5:8982795-8982816 | (GT)11 | F: CCAGACGTAAGTGATGAGATTCC | 107 |
|  |  |  | R: GAATCTGAACTGGAGGTCTAGGA |  |
| SSR004 | Chr. 5:18112515-18112550 | (ATGCTA)6 | F: TATCACAATCCTCCTTGCTTGTT | 121 |
|  |  |  | R: TTGAGTAATTGCAAGAACCTTCC |  |
| SSR005 | Chr. 5:1148236-1148259 | (TG)12 | F: ATCAATATGCATGTGGCTTTTTC | 160 |
|  |  |  | R: ACAATTCAAACACATACATTCTGGA |  |
| SSR006 | Chr. 5:10456698-10456711 | (TC)7 | F: CATATTATAGCTCGCCAGAAGGA | 120 |
|  |  |  | R: ATCCCAAGAGACAAACAACTGAA |  |
| SSR007 | Chr. 5:17891627-17891651 | (GAGTT)5 | F: GCATAAATAGGAGTGAGAGAGTCACA | 119 |
|  |  |  | R: TGAATTCTCAACATCAAGAGTGC |  |
| SSR008 | Chr. 1:42700588-42700614 | (AAC)9 | F: CCTTCAAAGATTTCCAAAACCAC | 137 |
|  |  |  | R: CGAGTCCCAATGTACTCTATTGC |  |
| SSR009 | Chr. 1:8494096-8494123 | (TCCA)7 | F: AACTCTTATCCCAAGGTGTACCG | 158 |
|  |  |  | R: CGTGGTCAATTAAAGATGGTGTT |  |
| SSR010 | Chr. 1:27435517-27435528 | (TA)6 | F: TATCAAGTTGCTTCAATTGGTTG | 147 |
|  |  |  | R: GACTTTCACCATCACCGTAAAAA |  |
| SSR011 | Chr. 1:29845374-29845394 | (TCC)7 | F: TTGGTAGGGGTAATAATAATCCG | 158 |
|  |  |  | R: AGACAAAATGATAAAAGTCCGCA |  |
| SSR012 | Chr. 1:43511395-43511424 | (CAAAAA)5 | F: TAGGAGTGAAAAAGAGAGCAAGC | 147 |
|  |  |  | R: GTTGGCTGGATAAAGGGATAAAT |  |
| SSR013 | Chr. 1:8649075-8649090 | (AT)8 | F: AAATTATAACTTCCAACTGCCCC | 130 |
|  |  |  | R: AAAGTTCACACCTTAGCAGCAAC |  |
| SSR014 | Chr. 1:40488274-40488287 | (TG)7 | F: GGTTTCAGAGCTATGCAAGACAG | 101 |
|  |  |  | R: ACGTACGCGCAGTCTACTGTAAG |  |
| SSR015 | Chr. 1:23853202-23853226 | (GATTG)5 | F: TTCACTGCAATTCCATGAGTAAA | 158 |
|  |  |  | R: CCGTGACACAAACCAATAACACT |  |
| SSR016 | Chr. 1:6872789-6872804 | (GA)8 | F: GTAGGACCACATCATGACATCAG | 132 |
|  |  |  | R: GCCGCATATAGTTTGTATGGTGT |  |
| SSR017 | Chr. 1:672942-672959 | (CGC)6 | F: TTGGTGTAGCAAACTTGGAAACT | 131 |
|  |  |  | R: TGGATTTCCATAATCAACAGAGC |  |
| SSR018 | Chr. 1:8856527-8856540 | (AG)7 | F: GATTTCAAATTAGCATGCACAGG | 150 |
|  |  |  | R: AATATGGGCAAAGAGTAGCTTCA |  |
| SSR019 | Chr. 1:32215530-32215547 | (CTC)6 | F: AGGATGCGTAGCTTCTAAGTTGA | 134 |
|  |  |  | R: TCAAAGCGTAACAGCATTCTACA |  |
| SSR020 | Chr. 1:29415857-29415870 | (TA)7 | F: AGCAAAATGTACCTCCTCAACTG | 116 |
|  |  |  | R: TCCAACCAACAACACAGTTACAC |  |
| SSR021 | Chr. 1:32578367-32578396 | (CCCAA)6 | F: CTCCTCGTGAACTTGGCTATAAA | 154 |
|  |  |  | R: AACCTTCAACATCATCTTCTTGG |  |
| SSR022 | Chr. 1:4497382-4497397 | (TC)8 | F: CATATGAAGAGACGCAGATCCTC | 89 |
|  |  |  | R: ATGCATAGGCACTTCTCAGTCTC |  |
| SSR023 | Chr. 1:27349716-27349739 | (TCCGAG)4 | F: GATTCCAGATTCCGACTCTTCTC | 156 |
|  |  |  | R: CCTAGGATTACAGCAGCAACAAG |  |
| SSR024 | Chr. 1:43222429-43222442 | (TA)7 | F: GTTGCAGCATCTGCTAATTCTTT | 129 |
|  |  |  | R: TTTGACTGAACATGGTGAACATT |  |
| SSR025 | Chr. 1:35212494-35212529 | (CCTCTA)6 | F: CGCTTGCTCTTGAGGAAGTAATA | 122 |
|  |  |  | R: TAACTCGTCAAGTTTCAAGAGGG |  |
| SSR026 | Chr. 1:24820585-24820596 | (CA)6 | F: TTATATAAACAAAATGGGCCAGC | 150 |
|  |  |  | R: CTGCTGTTTCTCCTACCCCTATT |  |
| SSR027 | Chr. 1:6192071-6192120 | (CT)25 | F: TCTCTACCTCACTACTTGGCACC | 155 |
|  |  |  | R: CGAAGGTGTAAAATTTTGGTGAG |  |
| SSR028 | Chr. 1:24429426-24429445 | (AG)10 | F: CAAAAATTCTTGAGGGAGAGACA | 102 |
|  |  |  | R: CCAGGTCCATGATTTCTGATTTA |  |
| SSR029 | Chr. 1:34221165-34221178 | (CT)7 | F: ATCTCTTGCTCTCTCCATATCCC | 125 |
|  |  |  | R: CATGGAGCCTAAACCATACAGAG |  |
| SSR030 | Chr. 1:8626510-8626527 | (ACG)6 | F: TTTAGGGTATTCAAACTTCGCAA | 157 |
|  |  |  | R: AGGGTGGTGAGTTTGATAGTTCA |  |
| SSR032 | Chr. 1:680184-680195 | (AC)6 | F: AATCAGATACAAACAGAGGCCAA | 115 |
|  |  |  | R: GGTCAATGGTGTGCAAGATATTC |  |
| SSR033 | Chr. 1:43912759-43912778 | (TC)10 | F: CCACATAATGACAGTGAACTTGC | 152 |
|  |  |  | R: GCTGCTCTACAATAGGAATGAGG |  |
| SSR034 | Chr. 1:29895383-29895398 | (GA)8 | F: AAAGACCTCCCTGTCAAAACAGT | 140 |
|  |  |  | R: AGCAAGTAATTGCACACCAAAAT |  |
| SSR035 | Chr. 1:647580-647593 | (TA)7 | F: TCGAGTGGTATCACAAACTTGAA | 141 |
|  |  |  | R: GCTATTGCTCATTTTCTCCTGTG |  |
| SSR036 | Chr. 1:32230757-32230781 | (CAACC)5 | F: AGAGCCAAGCTGTAATTGTGGTA | 144 |
|  |  |  | R: TGCTTAAGCGTTACCTAATGGTG |  |
| SSR037 | Chr. 1:32873146-32873173 | (CCTG)7 | F: ACTGTTGTTTAAATTCCTGGGGT | 116 |
|  |  |  | R: GATCAGATGTCACCACCACCTAT |  |
| SSR038 | Chr. 1:16051557-16051570 | (GT)7 | F: AATTCTTGCGTGCATATCGTAAT | 140 |
|  |  |  | R: TGGACGACTTTTGTGTTAAGGTT |  |
| SSR039 | Chr. 6:6864568-6864594 | (CAA)9 | F: CAGCTCAAATGGGATCTACAAGA | 132 |
|  |  |  | R: GGTTTGACATATTGTGGGAAGAA |  |
| SSR040 | Chr. 6:26927906-26927935 | (ACACAA)5 | F: CTCTTGTATGCTCACTTGTGTCG | 156 |
|  |  |  | R: AGAAATTGTGTATGGCTTTTCCC |  |
| SSR041 | Chr. 6:66100-66143 | (AG)22 | F: GAAAACCCAACTCCTCCTCTTTA | 136 |
|  |  |  | R: TTGACTCGAAACCCATTAACACT |  |
| SSR042 | Chr. 6:13087949-13087962 | (GA)7 | F: TGGCCAGTCTACTCTATAACCCA | 123 |
|  |  |  | R: AAATTCATTTGGCATTTCTTCTG |  |
| SSR043 | Chr. 6:24149922-24149937 | (CT)8 | F: ATCCTTTTCCCACACACTCATAC | 155 |
|  |  |  | R: CAAGGCAGAAGCTAAAGTCAAAG |  |
| SSR044 | Chr. 6:28674831-28674848 | (AT)9 | F: TAGCAGTGAAATAGCAGCAATGA | 135 |
|  |  |  | R: CCTTTAAACACACAGCTGGAAAG |  |
| SSR045 | Chr. 6:20908825-20908840 | (AT)8 | F: CAAGTGTGGGATCGATAGATAGG | 153 |
|  |  |  | R: CATGCTTACCGTTTCAGTACGTT |  |
| SSR046 | Chr. 6:1072811-1072840 | (TCCCTA)5 | F: GATGGTTTGACTGAAAGGATTGA | 158 |
|  |  |  | R: CTATTCTACAGCTGCTTCAAGGC |  |
| SSR047 | Chr. 6:7678381-7678396 | (GA)8 | F: CAATTGCAAAACATCAAATTCAA | 101 |
|  |  |  | R: AGCAATTTCCAGCATCAATTCTA |  |
| SSR048 | Chr. 6:25359239-25359263 | (GGACA)5 | F: TGCACTTGATTTATCCACACAAA | 129 |
|  |  |  | R: GAGGTTTGTATTGTATGTGGCGT |  |
| SSR049 | Chr. 6:24973273-24973297 | (CAACC)5 | F: CACTGTGCTGTTCTGTGTTAAGC | 113 |
|  |  |  | R: TATGAGTAAGAAATCGGTTGGCT |  |
| SSR050 | Chr. 6:22378409-22378422 | (TG)7 | F: TTAACTTTAATCTCGGCATGTGG | 125 |
|  |  |  | R:CCTAAAGAGAGCCACACATCTCA |  |
| SSR051 | Chr. 7:20594752-20594765 | (AT)7 | F: CATTTTACTTTTAGTCATGGCCG | 135 |
|  |  |  | R: AATGTGGACAGGTTAGACTTTGG |  |
| SSR052 | Chr. 7:17689006-17689029 | (CCG)8 | F: CATCACCACAACAACCACTACAG | 149 |
|  |  |  | R: GTTGATGTTGTCCATGTGTCAGA |  |
| SSR053 | Chr. 7:17594873-17594976 | (CGG)7 | F: CTGCTACAAGTGTGGTGAGTTTG | 102 |
|  |  |  | R: CCACAGCTATAACAAGCACCAG |  |
| SSR054 | Chr. 7:19621456-19621526 | (CT)7 | F: ATTTCTTTTCATCCGTTCCTTTC | 135 |
|  |  |  | R: CTATAAAGGATGTGTTGCCTTGG |  |
| SSR055 | Chr. 7:19608613-19608626 | (CT)7 | F: ACACTCAGTGGAAGTCACTCGTC | 147 |
|  |  |  | R: AGGTCTCATCGAAACCCTAAAAC |  |
| SSR056 | Chr. 7:21272455-21272468 | (GT)7 | F: TTGTGCTGCCAGATATATTCAAA | 156 |
|  |  |  | R: ACGATCAGAATCTCTAGCACAGC |  |
| SSR057 | Chr. 7:17827510-17827525 | (CT)8 | F: TGTGATTCTCTACAGCTTTTCCA | 112 |
|  |  |  | R: CAGAAGGTGGGTTATGCTAAACA |  |
| SSR058 | Chr. 7:22488447-22488470 | (CCAT)6 | F: ACAAGTTGAACCAAGCTGTCACT | 155 |
|  |  |  | R: GATTGTTGTAATCCATTTTCGGA |  |
| SSR059 | Chr. 7:15567688-15567701 | (AC)7 | F: CAAAAGCAACAACCCAATAATGT | 143 |
|  |  |  | R: CCTTTGATTGATGTTGATGATGA |  |
| SSR060 | Chr. 7:12649925-12649960 | (TC)18 | F: ATGCCCATAATGTAATCACCAAG | 156 |
|  |  |  | R: AGTTGAAGAAGCACTTTGAGGAA |  |
| SSR062 | Chr. 7:22438561-22438584 | (AAAG)6 | F: AATGAATACAAGCAAGTAGGCCC | 148 |
|  |  |  | R: GCAACTTGAACTCATCAGTTGTG |  |
| SSR063 | Chr. 7:18784845-18784860 | (TC)8 | F: TTTTGGGCTAACAAAGAGTCAGA | 142 |
|  |  |  | R: ATTGATGCATTTGACATGGAACT |  |
| SSR064 | Chr. 4:13234281-13234294 | (CA)7 | F: GCTACAAATTTTGGCAACCATTA | 132 |
|  |  |  | R: GAAGGTCTGCTTTGCACATAATC |  |
| SSR065 | Chr. 4:14821890-14821905 | (GA)8 | F: CTGTTCTTGGTCCTCATTGTTCT | 113 |
|  |  |  | R: CTTCCCTCTCTCCTTCAAACATT |  |
| SSR066 | Chr. 4:17074392-17074487 | (TA)7 | F: ACATTTACAAAATTGCCCATGTC | 82 |
|  |  |  | R: GACTGTTCAGACTGCCTTTTGAG |  |
| SSR067 | Chr. 4:3794824-3794853 | (TTGGGA)5 | F: ATTGGGTTTCATCAAGTCTTCCT | 156 |
|  |  |  | R: GAACCACTGTGCTGAATTAGCTT |  |
| SSR068 | Chr. 4:712810-712827 | (CAA)6 | F: CAAACACCACTACTCCGTTTCAG | 157 |
|  |  |  | R: TGTTGTCCATGAAATAACAGGC |  |
| SSR069 | Chr. 4:29864754-29864774 | (AGT)7 | F: TGCTGCCTTTCACTATCACTATG | 155 |
|  |  |  | R: AAAGTCCCAAAACACTCGAAAA |  |
| SSR070 | Chr. 4:23698592-23698605 | (TC)7 | F: ACACAGAGGAAAGGTCGTTATTG | 106 |
|  |  |  | R: AAGACCAAAGCAGCAGCTAGTTA |  |
| SSR071 | Chr. 4:6389320-6389349 | (GTTGAT)5 | F: CATATTGTAATGGAGCATGCGTA | 138 |
|  |  |  | R: AATACTTTGGAATTGCAACATGC |  |
| SSR072 | Chr. 4:6313549-6313592 | (CT)22 | F: AGTAAGACGACAAAGAGTGCCTG | 116 |
|  |  |  | R: CAGATGGCAGATTACATAGGCTT |  |
| SSR073 | Chr. 4:29833093-29833110 | (CAA)6 | F: TTGCTGCTGAAAAATAATGAACA | 160 |
|  |  |  | R: GGGTGGCCTGTTGAGAATATAA |  |
| SSR074 | Chr. 4:10665921-10665950 | (CCAGA)6 | F: GGTTTTTCAGTTCAACGTACCCT | 125 |
|  |  |  | R: AGCTGCAGAGAAAATGTGAAGAC |  |
| SSR075 | Chr. 4:138833-138862 | (ACTGG)6 | F: AAAATGAACTGGAACACAACACC | 93 |
|  |  |  | R: ATTGATCAGAAGCTGACTTTGCT |  |
| SSR077 | Chr. 4:425529-425554 | (AG)13 | F: ACCTTTAACTAAATTGGGGCGTA | 156 |
|  |  |  | R: TGATCCCTCCAACAAACATATTC |  |
| SSR078 | Chr. 4:11962981-11963008 | (CTGC)7 | F: GCTTTCTGGTCTTCTTCCTCACT | 134 |
|  |  |  | R: ATCTCTGCCCTAATTCCCAAATA |  |
| SSR079 | Chr. 3:16015253-16015276 | (TGACCA)4 | F: AGCTTGAGCAAAAACTATGCTGT | 150 |
|  |  |  | R: TGTTCATTTGGACCACCTATTGT |  |
| SSR080 | Chr. 3:20842580-20842609 | (TCAACT)5 | F: TAATAGTGACCCAACGCAACTTC | 99 |
|  |  |  | R: GTAGAAGGGGTCGTCTAGTGGAG |  |
| SSR082 | Chr. 3:21638293-21638322 | (ACCCTG)5 | F: GCCTAGCAACTCAGAAGCAACTA | 153 |
|  |  |  | R: GGAGTTTTGGATTAAGTTAGGGC |  |
| SSR083 | Chr. 3:5422383-5422396 | (TG)7 | F: TCTTTCTCTTCCATTGCTCTTCTT | 142 |
|  |  |  | R: GACTTTTTATTCCCTACCCAACG |  |
| SSR084 | Chr. 3:10530072-10530086 | (ATC)5 | F: GGATATGCATCCAGTCTCATCAT | 151 |
|  |  |  | R: ATAGTTGCTCTCCATCAACATCC |  |
| SSR085 | Chr. 3:13762829-13762842 | (AT)7 | F: AGGCTTTGTTGTGTTGTCAAGTT | 129 |
|  |  |  | R: TTTGCTAAGTTGGGGATAGTACG |  |
| SSR086 | Chr. 3:17609481-17609498 | (GGA)6 | F: TTCAATTTAGGCAGTTCAGTTGG | 123 |
|  |  |  | R: TGCACTTGATATGTATTTGACGC |  |
| SSR087 | Chr. 3:21497636-21497675 | (GATG)10 | F: GGAAACAACATGAAGAAAAGGTC | 139 |
|  |  |  | R: AAGGGAAGGCTCTACGAGTTCT |  |
| SSR088 | Chr. 3:20464537-20464550 | (CT)7 | F: GCTTCTCATAGCCACTGATTTTC | 102 |
|  |  |  | R: GGTCATGATCCCAAAATAAACAA |  |
| SSR089 | Chr. 3:15301794-15301807 | (TG)7 | F:ACCATGATTAGACAACATGGGTT | 159 |
|  |  |  | R: AGAGATTCTTGGGAATTAGGCAG |  |
| SSR090 | Chr. 3:2182428-2182473 | (AG)23 | F: TTCCATTGCTAATGTTTTTCCAC | 149 |
|  |  |  | R: CAGGAAGGCTTCTTATCTACCGT |  |
| SSR091 | Chr. 8:20478179-20478192 | (TG)7 | F: AGATTCACCAGAGAAACCCAACT | 157 |
|  |  |  | R: CCATTCAAGAACCAGAAGAAGAA |  |
| SSR092 | Chr. 8:18252699-18252712 | (AT)7 | F: CATGTTGGAGTGGGCTTAGATAG | 157 |
|  |  |  | R: AGCATTTTGATACGTTGATCGTC |  |
| SSR093 | Chr. 8:16601301-16601397 | (AG)10 | F: AACTGCCTTAGCTTAGACTGGCT | 160 |
|  |  |  | R: AAGACGAGAAACCACCTTGAATC |  |
| SSR094 | Chr. 8:13881722-13881743 | (GA)11 | F: ATACATTTCTCAACCACGAGACG | 131 |
|  |  |  | R: AGTTGGAACCCTAAATTCCTTCA |  |
| SSR095 | Chr. 8:13674394-13674453 | (TC)30 | F: AAAATTGAACTCGCATCAAATGT | 134 |
|  |  |  | R: AAACAGATGCACCAGGCTTAAT |  |
| SSR096 | Chr. 2:25847748-25847813 | (AG)33 | F: AACCTCAATCATTCTTTACACAAGC | 146 |
|  |  |  | R: CTGCTTAAGGAGGAACCTCAAAT |  |
| SSR097 | Chr. 2:20412279-20412296 | (GA)9 | F: GTTGATGCTATTGCAGCTTCTTC | 97 |
|  |  |  | R: GAAGCTCACCAATTGTTCTTCAG |  |
| SSR098 | Chr. 2:24454293-24454316 | (CAACTG)4 | F: GTGGCCATTGTAACATCAACTTC | 136 |
|  |  |  | R: CGGGATCCCACCTCTATATTTAC |  |
| SSR099 | Chr. 2:16756765-16756780 | (TC)8 | F: GTTTCAAGAGTTTGGTGAAAACG | 140 |
|  |  |  | R: ACCCTGTATCCAATGTCCTTCTC |  |
| SSR100 | Chr. 2:16796519-16796534 | (AG)8 | F: AGAATTTTATCGGGAAACAAACA | 140 |
|  |  |  | R: TCAGTCACTCTGCACACAAATCT |  |
| SSR101 | Chr. 2:16105382-16105399 | (GA)9 | F: AGGGCTTAAGAAGATATTGGTGG | 143 |
|  |  |  | R: ATTTTTCTTTGTGCTCTCATTGG |  |
| SSR102 | Chr. 2:7226201-7226238 | (TC)19 | F: AATGGTGAATGCTAGGTATGTGG | 128 |
|  |  |  | R: AAGAAATCCTAATTCTCAAATGAA |  |
| SSR105 | Chr. 2:26552979-26552992 | (GT)7 | F: CTACTGCAGTTGTTGATGCTCTG | 158 |
|  |  |  | R: TGATCTGAAAAAGTGAAAAAGAAAAA |  |
| SSR106 | Chr. 2:23933211-23933240 | (GGGAA)6 | F: TGGAGTGTAAATTTGTGGATGC | 160 |
|  |  |  | R: AGCAGTTAGGTGTTGGTTCACAT |  |
| SSR107 | Chr. 2:18644503-18644518 | (GA)8 | F: TGCAGACTAGGGTTTTACAGACAA | 155 |
|  |  |  | R: GATCTCCAAGTCATCTCCATCTG |  |
| SSR108 | Chr. 2:9511511-9511524 | (GA)7 | F: ACTAAGGGGAAGAAAAGCAACAG | 145 |
|  |  |  | R: AACTTATCGTCCATTTCCATTCA |  |
| SSR109 | Chr. 2:26576205-26576228 | (TCTG)6 | F: TTCAATTTTCATTTTCCTTTTGC | 99 |
|  |  |  | R: AACCGAACCCACCATATAACCTA |  |
| SSR110 | Chr. 2:281271-281427 | (AG)33 | F: ATATGGGTTTCTATGGAGTGACG | 130 |
|  |  |  | R: CTGCTAACCCATTCTCTCCCT |  |
| SSR111 | Chr. 1:567913-567931 | (ATT)6 | F: TCATCATTTCTTCATCTTGATGCT | 107 |
|  |  |  | R: CTATACATTTGGGCATTGGCTT |  |
| SSR112 | Chr. 6:23930205-23930219 | (TA)7 | F: ACGATGACATGGTTGTATCCTCT | 125 |
|  |  |  | R: AACCAGCAGTTAGGTTCATTTCC |  |
| SSR113 | Chr. 4:11537152-11537226 | (CT)37 | F: AATACCTCAAAATCCCCTCTCC | 160 |
|  |  |  | R: GGAATTTTCCCCATAATATTCGT |  |
| SSR115 | Chr. 5:18182884-18182996 | (CT)28 | F: GCCGTTTCATTTCAATTTTCTAA | 131 |
|  |  |  | R: AAAGCAAAAACAGAAACCCCTAT |  |
| SSR116 | Chr. 5:10118815-10118834 | (AG)10 | F: TACACGTTTTGAAAAACCCTAGC | 89 |
|  |  |  | R: GGAGAACACTGAGGATCCAATG |  |
| SSR118 | Chr. 1:46623238-46623273 | (CCCTAA)6 | F: TAGACCTATTGGCCATTTAACCA | 135 |
|  |  |  | R: TATCCAAGACTCACATTCCACCT |  |
| SSR119 | Chr. 1:37886776-37886880 | (GA)11 | F: GGCTCAGCTTAACTTCCACTTTT | 119 |
|  |  |  | R: CTCCCTCTCTCAATCTTTTCCTT |  |
| SSR120 | Chr. 1:35412728-35412749 | (TG)11 | F: ATTTCTGTGCCTCTTCCATCAC | 149 |
|  |  |  | R: GTCACTTGGTACATTTGAGGCTT |  |
| SSR121 | Chr. 1:8809311-8809352 | (AGCCCC)7 | F: AACCCACATCTTGTTTTCTCTGA | 145 |
|  |  |  | R: ACAAGAGATGAATTGGGACTCG |  |
| SSR122 | Chr. 1:39500301-39500333 | (CGG)11 | F: GACGAGCTTATCTCTCACAGGAG | 155 |
|  |  |  | R: CAGCTAAACGCAGCAATCTCT |  |
| SSR123 | Chr. 6:2324686-2324733 | (GA)24 | F: TGCACCAAACTGAGATATTAGGA | 153 |
|  |  |  | R: TGCTGGGAAGAACCTGTAACTAA |  |
| SSR125 | Chr. 6:22369258-22369335 | (GA)39 | F: TAGCGCCATTGTTCACACAC | 156 |
|  |  |  | R: GCTGGGAGAGAAAGATGACTGT |  |
| SSR127 | Chr. 7:2034707-2034756 | (GA)25 | F: TCTTTTACAAATGTGGGGAGAGA | 157 |
|  |  |  | R: CCTATTTCACACATGTTATGCCA |  |
| SSR128 | Chr. 7:19615161-19615202 | (CT)21 | F: GTACGTAAAACGTCAAGTCCCAC | 160 |
|  |  |  | R: CTCTTAAGCGTTCCCCTGTCT |  |
| SSR130 | Chr. 4:15159645-15159698 | (AG)27 | F: ACCACATATGAGTATATTAAAGCAAA | 155 |
|  |  |  | R: CAACTGCCGGTCTTCATCTT |  |
| SSR131 | Chr. 4:12095224-12095241 | (TC)9 | F: TGCTGGTTTAACAAAGTCATGG | 152 |
|  |  |  | R: TTGCAAATTAGGATCTCAGGTTC |  |
| SSR132 | Chr. 4:3949640-3949675 | (CTGC)9 | F: GCATGCAGCTTTTGTTAATTTTT | 151 |
|  |  |  | R: GTAAATCTGCAAAGAAACGGACA |  |
| SSR133 | Chr. 4:13182865-13182920 | (GA)28 | F: ATATACCCGGATGGATAGCAGAT | 160 |
|  |  |  | R: TATGAGTGCACCTCCAGTAACAA |  |
| SSR134 | Chr. 3:21933965-21934016 | (GA)26 | F: AAGACAACGACAATTAACTTGGG | 153 |
|  |  |  | R: GTCCTTTGGTTTTAATTCTTTCG |  |
| SSR135 | Chr. 3:14172367-14172478 | (AG)56 | F: AAAAGGAATTGAAGAGAAAGATACT | 234 |
|  |  |  | R: TATCTATGCTCGTGGGTATGTTC |  |
| SSR136 | Chr. 8:16514725-16514740 | (AG)8 | F: CTTCAACCCCACAAGTTTCATAC | 84 |
|  |  |  | R: ATCCAACTAGACCACGACCTACA |  |
| SSR137 | Chr. 8:2591629-2591668 | (AG)20 | F: GGGCTCCTTGAAAATTAATATGTG | 150 |
|  |  |  | R: CCAACCCAATTGATTCTTCTCT |  |
| SSR138 | Chr. 8:5377109-5377164 | (GA)28 | F: TCCATGCTTCTTTGTAATGACAGT | 157 |
|  |  |  | R: CCAGGTAAATGTGAATCGAGACT |  |
| SSR139 | Chr. 8:13404378-13404439 | (AG)31 | F: ACAAACTACACACAAACACATCCA | 120 |
|  |  |  | R: CTTTGTGTTCTTTAAGGCCTGTG |  |
| SSR143 | Chr. 2:18442542-18442561 | (TC)10 | F: GAGTTCAATATACAGACAGCACCG | 133 |
|  |  |  | R: GCCATGTTGAAGTGAAAAGGATA |  |
| SSR144 | Chr. 2:16920533-16920552 | (TC)10 | F: AACCCAGGCTTTATTTGTTCTCT | 151 |
|  |  |  | R: GCAATGTTAATTGTACTTTGTCTCCA |  |
| SSR145 | Chr. 2:12523293-12523322 | (AT)15 | F: TAAATGTGGTCATTGCATGCTT | 122 |
|  |  |  | R: ACAAGTGGTGTCCCTCAAATAAG |  |
| SSR146 | Chr. 2:26236767-26236784 | (CA)9 | F: AGTCTGCATGGAGATCACAGATT | 121 |
|  |  |  | R: TTGATTCCACTTCCTTAATTTGG |  |
| SSR147 | Chr. 2:20518138-20518177 | (CATC)10 | F: TTACAGTTGAGCTTGAGGGAGTC | 96 |
|  |  |  | R: GAGGCTGCCACTTTTATCTGAC |  |
| SSR148 | Chr. 1:7059108-7059122 | （AG)7 | F: GGCAAAGAAGAAGTCACAGAGAA | 132 |
|  |  |  | R: AACCCTAGCACATCTCTTCCTTT |  |
| SSR149 | Chr. 1:40845640-40845684 | （GA）22 | F: CTTTATGGTATTCCAAAGCAAGG | 101 |
|  |  |  | R: CATCACTTTGTTCATGTTTGACA |  |
| SSR150 | Chr. 1:35515894-35515928 | (GA)17 | F: CCGCTCAGTAATCTATCCGA | 284 |
|  |  |  | R: ACTTAATCCGATTCGAGAACG |  |
| SSR151 | Chr. 1:29349630-29349696 | (GT)33 | F: AGGATTTTGCTGTGCTTGTCT | 351 |
|  |  |  | R: AACCCATATCAGTTCTCACTTTCT |  |
| SSR152 | Chr. 1:1400594-1400656 | (TC)31 | F: GTTCTCGACTCCCATATCCAA | 250 |
|  |  |  | R: CTCCAAAGTACAGAGCCTATCG |  |
| SSR153 | Chr. 6:21578981-21578995 | (CT)7 | F: ATTCGGCTCTCGGGTCAG | 300 |
|  |  |  | R: ATCAGGCAGAGAACATCATCAA |  |
| SSR154 | Chr. 7:1792352-1792402 | (TC)25 | F: GTGAAAGAGAGTGGCACAGAGT | 226 |
|  |  |  | R: CGTCCGTGCGGTAACAAT |  |
| SSR155 | Chr. 7:20664270-20664328 | (CT)29 | F: ATCAATCAGCACCGTCCACT | 254 |
|  |  |  | R: CTGCCTCTTCACCCACTTCA |  |
| SSR156 | Chr. 7:7176865-7176883 | (GA)9 | F: AGGTCTGCTATGTCAGGGAAG | 172 |
|  |  |  | R: TTATGAAATGCCGCAGGAT |  |
| SSR157 | Chr. 7:17753511-17753521 | (AG)5 | F: GACAAACAACACCGAAGCA | 122 |
|  |  |  | R: GTCTCTGATGAAAACTCCCACTC |  |
| SSR158 | Chr. 4:18554422-18554436 | (CT)7 | F: ATTACAATTTGTGTCGGCTTCT | 252 |
|  |  |  | R: GCTTGGAGGTCCTTGATTTC |  |
| SSR159 | Chr. 3:19647288-19647304 | (AC)8 | F: TCTCATCTCATGGATATCATCACT | 270 |
|  |  |  | R: AGGTTCTACAAGAATGTCCTTCAC |  |
| SSR160 | Chr. 8:20181238-20181258 | (GA)10 | F: TAGAAGACCAAATGGAGGAGTG | 281 |
|  |  |  | R: TTACCCCAAAATCCAAGGC |  |
| SSR161 | Chr. 1:26942753-26942807 | (TG)27 | F: GAAGTTTGTAGAACTCTGGAGGAT | 194 |
|  |  |  | R: ATTGACCCTTGAGCCTGTTAG |  |
| SSR162 | Chr. 1:19603403-19603517 | (GA)57 | F: GTCTGCAAACGAAAAGACCG | 318 |
|  |  |  | R: TCTGGATAGAATTACCACGACG |  |
| SSR163 | Chr. 7:19613713-19613731 | (TG)9 | F: GCTGGACCAAATTATCTCAACT | 167 |
|  |  |  | R: AAAACCATTATGCTTTGCTTCT |  |
| SSR169 | Chr. 7:9912425-9912467 | (CT)21 | F: TGCCTACGTGGCACTATAGGT | 267 |
|  |  |  | R: TCCAACCAAGCATCACCATC |  |
| SSR170 | Chr. 7:16025056-16025086 | (TC)30 | F: TTCTCGTAACCAAACACAACC | 337 |
|  |  |  | R: ATGTGACAAATTTTGGGTTCC |  |
| SSR171 | Chr. 7:9493191-9493207 | (AG)8 | F: CTACAAAATTCATCCCAAAGCT | 228 |
|  |  |  | R: TTCTCATGCCTATCCTGACCTA |  |
| SSR172 | Chr. 4:712810-712828 | (CAA)18 | F: CAACCGCAATAGCAGCAC | 154 |
|  |  |  | R: TTGACAGAAGAAGAGGAAGAGC |  |
| SSR173 | Chr. 4:23667101-23667115 | (TA)7 | F: TGGAAACAAGACAAATGAAGAGA | 144 |
|  |  |  | R: CGGTTTGCAATATTCAAAATCA |  |
| SSR174 | Chr. 8:18696336-18696368 | (TC)16 | F: TTCTCTCCTCACACACACCCTT | 340 |
|  |  |  | R: TGAAACACATGAACCATGCTCT |  |
| SSR176 | Chr. 2:25906737-25906775 | (AG)19 | F: TCCTCATCATCGCGGAAGA | 254 |
|  |  |  | R: CGTCGCACTGTAGACGTTAGTG |  |
| SSR177 | Chr. 2:9310634-9310666 | (CT)16 | F: TTGATGAAACTCCTCCCACC | 159 |
|  |  |  | R: ATGGAAAAATTAAGCATCAGAGA |  |
| SSR178 | Chr. 2:1647323-1647339 | (AG)8 | F: ATCACGTCGGAAAGTTCCTAGA | 222 |
|  |  |  | R: CGCCCTCCTCCCTCAGTA |  |
| SSR179 | Chr. 1:33872028-33872044 | (AG)8 | F: AACAGTGGCCTTCCCTTGA | 220 |
|  |  |  | R: CAATTGTGTTGTTGGGTTATGA |  |
| SSR180 | Chr. 6:4799398-4799470 | (CT)36 | F: AGAATGCAGGCCTTCCTTCT | 224 |
|  |  |  | R: GCACCTTGCTTATCATCCGA |  |
| SSR181 | Chr. 7:18472710-18472725 | (GTA)5 | F: TCAATCTGATGAGATGAGCCAT | 188 |
|  |  |  | R: GTCAAAGATTACAACAGCCAGC |  |
| SSR182 | Chr. 4:26799178-26799226 | (CT)24 | F: TGAAATCTTGATGTGCCTTGTC | 221 |
|  |  |  | R: ACCACTCGTCAACGCCAAC |  |
| SSR183 | Chr. 3:3349593-3349659 | (GA)33 | F: TGAATGTTCTTCCTGCTCCTG | 290 |
|  |  |  | R: ATGAACATGAACCAGTCAAGGA |  |
| SSR184 | Chr. 3:3145374-3145392 | (TA)9 | F: TTGTGAGGAATATTATGCCTGC | 278 |
|  |  |  | R: GATGGTTTGGATCATTGGGA |  |
| SSR186 | Chr. 8:12058716-12058754 | (TC)19 | F: TCGACAGTTTGGGGTCTCAC | 325 |
|  |  |  | R: ACCTTAGTCCACGCCATTTG |  |

**Note:** ‘F’ indicates the forward primer and ‘R’ indicates the reverse.

**S4 Tab. Polymorphism and allele number estimation of PCR product amplifying using 164 SSRs among 21 peach accessions.**

| SSRs | Accessions | | | | | | | | | | | | | | | | |
| --- | --- | --- | --- | --- | --- | --- | --- | --- | --- | --- | --- | --- | --- | --- | --- | --- | --- |
|  | 105 | 110 | 114 | 118 | 151 | 157 | 160 | 164 | 169 | 194 | 221 | 37 | 70 | 78 | 84 | 85 | 99 |
| SSR001 | 160/160 | 160/160 | 160/160 | 154/160 | 160/160 | 160/160 | 160/160 | 160/160 | 160/160 | 160/160 | 160/160 | 160/160 | 160/160 | 160/160 | 160/160 | 160/160 | 160/160 |
| SSR002 | 123/123 | 123/123 | 123/123 | 123/123 | 123/123 | 123/123 | 123/123 | 123/123 | 123/123 | 123/123 | 123/123 | 123/123 | 123/123 | 123/123 | 123/123 | 123/123 | 123/123 |
| SSR004 | 136/136 | 136/136 | 136/136 | 136/136 | 136/136 | 136/136 | 136/136 | 136/136 | 136/136 | 136/136 | 136/136 | 136/136 | 136/136 | 136/136 | 136/136 | 136/136 | 136/136 |
| SSR005 | 178/178 | 178/178 | 226/226 | 226/226 | 178/226 | 178/226 | 178/226 | 178/226 | 178/226 | 226/226 | 178/178 | 178/178 | 178/226 | 226/226 | 178/178 | 178/178 | 178/178 |
| SSR006 | 137/137 | 133/133 | 137/137 | 137/137 | 133/137 | 133/137 | 133/137 | 133/133 | 133/133 | 137/137 | 137/137 | 133/133 | 133/137 | 133/133 | 137/137 | 137/137 | 133/137 |
| SSR007 | 133/133 | 133/133 | 133/133 | 133/133 | 133/133 | 133/133 | 133/133 | 133/133 | 133/133 | 133/133 | 133/133 | 133/133 | 133/133 | 133/133 | 133/133 | 133/133 | 133/133 |
| SSR008 | 156/156 | 156/156 | 153/153 | 153/156 | 156/156 | 156/156 | 156/156 | 156/156 | 156/156 | 156/156 | 156/156 | 153/153 | 156/156 | 153/156 | 156/156 | 153/156 | 156/156 |
| SSR009 | 170/170 | 170/170 | 170/174 | 170/170 | 170/174 | 170/174 | 170/174 | 170/174 | 170/174 | 170/170 | 174/174 | 174/174 | 174/174 | 174/174 | 170/174 | 170/170 | 174/174 |
| SSR010 | 172/172 | 172/172 | 162/172 | 162/176 | 172/172 | 172/172 | 172/172 | 172/172 | 172/172 | 162/176 | 162/162 | 162/162 | 162/162 | 162/162 | 162/162 | 162/162 | 162/162 |
| SSR011 | 170/170 | 170/170 | 170/173 | 170/173 | 170/170 | 170/170 | 170/170 | 170/170 | 170/170 | 170/173 | 170/170 | 170/170 | 170/170 | 170/170 | 170/170 | 170/170 | 170/170 |
| SSR012 | 163/163 | 163/163 | 163/163 | 163/163 | 157/163 | 163/163 | 157/163 | 163/163 | 163/163 | 163/163 | 163/163 | 163/163 | 157/163 | 157/163 | 157/163 | 163/163 | 163/163 |
| SSR013 | 146/146 | 148/148 | 148/160 | 148/158 | 144/148 | 144/146 | 144/148 | 144/148 | 144/148 | 146/146 | 144/146 | 146/146 | 146/146 | 156/156 | 146/148 | 148/148 | 146/146 |
| SSR014 | 113/115 | 113/113 | 113/115 | 113/115 | 113/115 | 113/115 | 113/115 | 113/113 | 113/113 | 113/113 | 113/115 | 115/115 | 113/115 | 113/115 | 113/113 | 115/115 | 115/115 |
| SSR015 | 172/172 | 168/172 | 168/172 | 168/172 | 168/168 | 168/172 | 168/168 | 168/168 | 168/172 | 172/172 | 172/172 | 172/172 | 172/172 | 168/168 | 172/172 | 172/172 | 172/172 |
| SSR016 | 183/183 | 183/183 | 183/185 | 171/171 | 179/183 | 179/183 | 179/183 | 149/179 | 179/183 | 171/189 | 149/179 | 149/149 | 149/149 | 180/180 | 149/183 | 149/149 | 149/149 |
| SSR017 | 145/145 | 145/145 | 145/145 | 142/142 | 145/145 | 145/145 | 145/145 | 145/145 | 145/145 | 145/145 | 142/145 | 145/145 | 145/145 | 142/142 | 145/145 | 145/145 | 145/145 |
| SSR018 | 165/165 | 214/214 | 214/214 | 192/214 | 214/214 | 165/214 | 214/214 | 214/214 | 214/214 | 165/165 | 165/214 | 165/165 | 165/165 | 214/214 | 165/214 | 214/214 | 165/165 |
| SSR019 | 148/148 | 148/148 | 148/148 | 145/148 | 148/148 | 148/148 | 148/148 | 148/148 | 148/148 | 145/148 | 145/148 | 148/148 | 148/148 | 148/148 | 145/148 | 148/148 | 148/148 |
| SSR020 | 132/132 | 130/132 | 130/132 | 130/132 | 130/132 | 130/132 | 130/132 | 130/130 | 130/132 | 132/132 | 130/130 | 130/130 | 130/130 | 132/132 | 130/130 | 130/130 | 130/130 |
| SSR021 | 167/167 | 167/167 | 167/167 | 162/167 | 158/167 | 158/167 | 158/167 | 158/167 | 158/167 | 167/167 | 162/167 | 162/162 | 162/167 | 167/167 | 162/167 | 162/162 | 162/167 |
| SSR022 | 104/104 | 104/104 | 104/104 | 104/104 | 102/104 | 102/104 | 102/104 | 102/104 | 102/104 | 104/104 | 102/104 | 104/104 | 104/104 | 104/104 | 104/104 | 104/104 | 104/104 |
| SSR023 | 171/171 | 171/177 | 171/171 | 171/177 | 171/171 | 171/171 | 171/171 | 171/171 | 171/177 | 171/177 | 171/171 | 171/171 | 171/171 | 171/171 | 171/171 | 171/171 | 171/171 |
| SSR024 | 145/145 | 145/145 | 145/145 | 145/145 | 145/145 | 145/145 | 145/145 | 145/145 | 145/145 | 143/145 | 145/145 | 145/145 | 145/145 | 145/145 | 145/145 | 145/145 | 145/145 |
| SSR025 | 137/137 | 137/143 | 143/143 | 137/143 | 137/137 | 137/137 | 137/137 | 137/143 | 137/137 | 137/137 | 137/137 | 143/143 | 137/137 | 131/143 | 137/137 | 143/143 | 137/137 |
| SSR026 | 167/167 | 167/167 | 186/197 | 167/184 | 184/184 | 167/184 | 184/184 | 167/184 | 167/184 | 167/167 | 167/167 | 167/167 | 167/167 | 184/184 | 167/167 | 167/167 | 167/167 |
| SSR027 | 161/161 | 161/161 | 161/161 | 167/167 | 145/161 | 145/161 | 145/161 | 145/169 | 145/161 | 167/171 | 145/169 | 169/169 | 169/169 | 161/161 | 161/169 | 169/169 | 169/169 |
| SSR028 | 117/117 | 113/117 | 113/117 | 113/117 | 113/113 | 113/117 | 113/113 | 113/113 | 113/117 | 113/117 | 117/117 | 117/117 | 117/117 | 113/113 | 117/117 | 117/117 | 117/117 |
| SSR029 | 138/138 | 138/138 | 138/138 | 138/140 | 138/138 | 138/138 | 138/138 | 138/138 | 138/138 | 138/140 | 140/140 | 138/138 | 138/140 | 138/138 | 140/140 | 138/138 | 138/140 |
| SSR030 | 173/173 | 173/173 | 170/173 | 173/173 | 170/173 | 170/173 | 170/173 | 170/173 | 170/173 | 173/173 | 170/173 | 173/173 | 173/173 | 170/170 | 173/173 | 173/173 | 173/173 |
| SSR032 | 133/133 | 133/133 | 133/160 | 133/150 | 133/133 | 133/133 | 133/133 | 133/133 | 133/133 | 147/166 | 133/166 | 133/133 | 133/133 | 160/160 | 133/133 | 133/133 | 133/133 |
| SSR033 | 168/168 | 168/168 | 168/184 | 168/184 | 168/184 | 168/168 | 168/184 | 168/168 | 168/184 | 168/184 | 168/168 | 184/184 | 168/184 | 168/200 | 168/184 | 168/184 | 168/168 |
| SSR034 | 167/167 | 155/167 | 155/173 | 155/173 | 155/167 | 155/167 | 155/167 | 155/155 | 155/167 | 155/155 | 155/155 | 155/155 | 155/155 | 155/155 | 155/155 | 155/155 | 155/155 |
| SSR035 | 155/157 | 155/155 | 155/161 | 157/157 | 155/157 | 155/157 | 155/157 | 155/157 | 155/155 | 157/157 | 155/157 | 157/157 | 155/157 | 163/163 | 157/157 | 157/157 | 155/157 |
| SSR036 | 161/161 | 161/161 | 161/161 | 156/161 | 156/161 | 156/161 | 156/161 | 156/161 | 156/161 | 156/161 | 156/161 | 161/161 | 161/161 | 161/161 | 156/161 | 161/161 | 161/161 |
| SSR037 | 131/131 | 131/131 | 131/131 | 131/131 | 131/131 | 131/131 | 131/131 | 131/131 | 131/131 | 131/131 | 131/131 | 131/131 | 131/131 | 131/131 | 131/131 | 131/131 | 131/131 |
| SSR038 | 150/150 | 154/154 | 154/154 | 152/152 | 150/154 | 150/150 | 150/154 | 154/154 | 154/154 | 152/152 | 154/154 | 154/154 | 154/154 | 152/152 | 154/154 | 154/154 | 154/154 |
| SSR039 | 145/145 | 145/145 | 145/145 | 142/142 | 145/145 | 145/145 | 145/145 | 145/145 | 145/145 | 142/142 | 145/145 | 145/145 | 145/145 | 145/145 | 145/145 | 145/145 | 145/145 |
| SSR040 | 167/167 | 162/167 | 162/162 | 162/162 | 162/167 | 167/167 | 162/167 | 162/167 | 162/167 | 162/162 | 162/167 | 167/167 | 167/167 | 162/167 | 167/167 | 167/167 | 162/167 |
| SSR041 | 121/151 | 121/151 | 155/155 | 121/151 | 151/151 | 121/121 | 151/151 | 121/147 | 121/151 | 145/145 | 121/147 | 151/151 | 151/151 | 121/121 | 151/151 | 151/151 | 147/151 |
| SSR042 | 157/157 | 155/161 | 137/137 | 157/157 | 155/161 | 157/157 | 155/161 | 157/157 | 157/161 | 137/137 | 157/157 | 157/159 | 155/157 | 137/157 | 155/155 | 157/157 | 157/157 |
| SSR043 | 205/220 | 181/205 | 199/205 | 222/228 | 181/205 | 205/205 | 181/205 | 181/205 | 181/205 | 209/217 | 170/205 | 170/170 | 170/201 | 195/201 | 170/170 | 170/170 | 170/201 |
| SSR044 | 105/150 | 105/150 | 105/150 | 105/150 | 105/150 | 105/150 | 105/150 | 105/150 | 105/150 | 105/148 | 105/150 | 105/148 | 105/150 | 105/150 | 105/150 | 105/148 | 105/150 |
| SSR045 | 166/168 | 166/166 | 166/166 | 168/168 | 166/168 | 166/166 | 166/168 | 166/168 | 166/166 | 166/166 | 168/168 | 168/168 | 168/168 | 168/168 | 168/168 | 168/168 | 168/168 |
| SSR046 | 174/174 | 174/174 | 174/174 | 168/168 | 174/174 | 174/174 | 174/174 | 174/174 | 174/174 | 168/168 | 168/174 | 174/174 | 174/174 | 168/168 | 174/174 | 174/174 | 174/174 |
| SSR047 | 114/114 | 114/114 | 114/114 | 114/114 | 116/116 | 114/114 | 116/116 | 116/116 | 114/114 | 114/114 | 116/116 | 116/116 | 116/116 | 114/114 | 116/116 | 116/116 | 116/116 |
| SSR048 | 143/143 | 143/143 | 143/143 | 143/143 | 143/143 | 143/143 | 143/143 | 143/143 | 143/143 | 143/143 | 143/143 | 143/143 | 143/143 | 143/143 | 143/143 | 143/143 | 143/143 |
| SSR049 | 122/122 | 122/126 | 126/126 | 122/122 | 122/126 | 122/122 | 122/126 | 122/126 | 122/126 | 122/122 | 122/122 | 126/126 | 126/126 | 126/126 | 126/126 | 126/126 | 126/126 |
| SSR050 | 136/136 | 136/136 | 136/138 | 136/138 | 136/138 | 136/136 | 136/138 | 136/138 | 136/136 | 136/136 | 136/138 | 136/138 | 136/136 | 136/136 | 136/136 | 136/136 | 136/138 |
| SSR051 | 148/148 | 148/148 | 148/150 | 148/150 | 148/148 | 148/148 | 148/148 | 148/148 | 148/148 | 148/148 | 150/150 | 148/148 | 148/150 | 148/148 | 150/150 | 148/150 | 150/150 |
| SSR052 | 155/155 | 155/161 | 155/155 | 155/161 | 155/155 | 155/155 | 155/155 | 155/155 | 155/155 | 155/155 | 161/161 | 161/161 | 155/161 | 155/155 | 155/161 | 155/161 | 161/161 |
| SSR053 | 111/111 | 111/111 | 111/111 | 111/111 | 111/111 | 111/111 | 111/111 | 111/111 | 111/111 | 111/111 | 114/114 | 114/114 | 111/114 | 111/111 | 111/114 | 111/114 | 114/114 |
| SSR054 | 148/148 | 148/150 | 148/150 | 148/150 | 148/150 | 148/150 | 148/150 | 150/150 | 148/150 | 150/150 | 148/150 | 150/150 | 150/150 | 150/150 | 150/150 | 150/150 | 148/150 |
| SSR055 | 184/184 | 182/184 | 164/184 | 164/184 | 182/184 | 182/184 | 182/184 | 182/182 | 182/184 | 182/182 | 164/184 | 164/164 | 164/182 | 186/186 | 164/164 | 164/182 | 164/184 |
| SSR056 | 169/169 | 169/169 | 167/169 | 167/169 | 169/169 | 169/169 | 169/169 | 169/169 | 169/169 | 167/169 | 169/169 | 169/169 | 169/169 | 169/169 | 169/169 | 169/169 | 169/169 |
| SSR057 | 127/127 | 127/127 | 127/127 | 127/127 | 127/127 | 127/127 | 127/127 | 127/127 | 127/127 | 127/127 | 127/127 | 127/127 | 127/127 | 127/127 | 127/127 | 127/127 | 127/127 |
| SSR058 | 165/165 | 165/169 | 165/173 | 165/165 | 165/165 | 165/165 | 165/165 | 165/169 | 165/165 | 169/169 | 169/169 | 169/169 | 165/169 | 165/169 | 169/169 | 169/169 | 169/169 |
| SSR059 | 157/159 | 157/157 | 157/157 | 157/157 | 157/157 | 157/157 | 157/157 | 157/157 | 157/157 | 157/159 | 159/159 | 157/157 | 157/159 | 157/157 | 159/159 | 157/159 | 159/159 |
| SSR060 | 175/175 | 175/175 | 177/177 | 173/173 | 175/175 | 175/175 | 175/175 | 171/175 | 175/175 | 177/177 | 171/171 | 175/175 | 171/175 | 179/179 | 171/175 | 175/175 | 171/171 |
| SSR062 | 166/166 | 166/170 | 166/170 | 170/170 | 166/166 | 166/166 | 166/166 | 166/170 | 166/166 | 166/166 | 166/166 | 166/166 | 166/166 | 166/170 | 166/166 | 166/166 | 166/166 |
| SSR063 | 153/153 | 153/155 | 153/157 | 157/157 | 153/153 | 153/153 | 153/153 | 153/153 | 153/153 | 153/155 | 157/157 | 157/157 | 153/157 | 157/157 | 155/157 | 153/157 | 157/157 |
| SSR064 | 150/150 | 150/150 | 150/150 | 150/150 | 150/150 | 150/150 | 150/150 | 150/150 | 150/150 | 150/150 | 150/150 | 150/150 | 150/150 | 150/150 | 150/150 | 150/150 | 150/150 |
| SSR065 | 125/129 | 125/125 | 129/129 | 129/129 | 125/125 | 125/129 | 125/125 | 125/125 | 125/125 | 125/125 | 125/129 | 129/129 | 125/125 | 129/129 | 125/125 | 129/129 | 125/129 |
| SSR066 | 96/96 | 96/96 | 94/94 | 96/96 | 96/96 | 96/96 | 96/96 | 96/96 | 96/96 | 94/94 | 96/96 | 96/96 | 96/96 | 96/96 | 96/96 | 96/96 | 96/96 |
| SSR067 | 167/173 | 167/167 | 167/167 | 167/173 | 173/173 | 173/173 | 173/173 | 173/173 | 167/173 | 167/167 | 173/173 | 173/173 | 173/173 | 173/173 | 173/173 | 167/167 | 173/173 |
| SSR068 | 165/168 | 165/165 | 165/165 | 168/168 | 165/168 | 165/168 | 165/168 | 165/168 | 165/165 | 165/165 | 165/168 | 165/165 | 165/165 | 165/165 | 168/168 | 165/165 | 165/168 |
| SSR069 | 172/172 | 172/172 | 172/172 | 172/172 | 172/172 | 172/172 | 172/172 | 172/172 | 172/172 | 169/172 | 172/172 | 172/172 | 172/172 | 172/172 | 172/172 | 172/172 | 172/172 |
| SSR070 | 120/129 | 120/129 | 120/120 | 131/131 | 129/129 | 120/129 | 129/129 | 120/129 | 129/129 | 120/120 | 120/120 | 120/120 | 120/120 | 131/131 | 120/120 | 120/120 | 120/131 |
| SSR071 | 149/155 | 149/149 | 155/155 | 155/155 | 149/155 | 149/155 | 149/155 | 149/155 | 149/149 | 155/155 | 155/155 | 155/155 | 149/155 | 149/149 | 155/155 | 149/149 | 155/161 |
| SSR072 | 123/129 | 119/123 | 129/129 | 129/131 | 123/129 | 127/129 | 123/129 | 127/129 | 123/127 | 131/131 | 129/129 | 129/129 | 127/129 | 119/119 | 131/131 | 119/119 | 131/131 |
| SSR073 | 176/215 | 176/215 | 189/189 | 205/205 | 215/215 | 176/215 | 215/215 | 176/215 | 215/215 | 176/189 | 176/176 | 176/176 | 176/205 | 196/196 | 176/176 | 176/176 | 205/208 |
| SSR074 | 142/142 | 142/142 | 142/142 | 142/142 | 142/142 | 142/142 | 142/142 | 142/142 | 142/142 | 147/147 | 142/142 | 142/142 | 142/142 | 142/142 | 142/142 | 142/142 | 142/142 |
| SSR075 | 109/109 | 104/109 | 109/109 | 109/109 | 109/109 | 109/109 | 109/109 | 109/109 | 109/109 | 104/109 | 104/109 | 104/114 | 104/109 | 114/114 | 104/104 | 104/114 | 104/109 |
| SSR077 | 161/173 | 161/161 | 161/161 | 173/173 | 161/173 | 161/173 | 161/173 | 161/173 | 161/161 | 173.173 | 161/173 | 161/173 | 161/161 | 173/173 | 173/173 | 161/173 | 161/173 |
| SSR078 | 144/152 | 144/144 | 144/144 | 152/152 | 144/152 | 144/152 | 144/152 | 144/152 | 144/144 | 152/152 | 152/152 | 152/152 | 152/152 | 144/152 | 152/152 | 152/152 | 144/152 |
| SSR079 | 172/172 | 172/172 | 172/172 | 166/166 | 166/172 | 172/172 | 166/172 | 166/172 | 172/172 | 166/166 | 166/172 | 172/172 | 166/172 | 166/172 | 166/166 | 172/172 | 166/166 |
| SSR080 | 113/113 | 113/113 | 113/113 | 113/113 | 113/113 | 113/113 | 113/113 | 113/113 | 113/113 | 113/113 | 113/113 | 113/113 | 113/113 | 113/113 | 113/113 | 113/113 | 113/113 |
| SSR082 | 167/167 | 161/167 | 167/167 | 161/167 | 161/167 | 167/167 | 161/167 | 161/167 | 167/167 | 161/167 | 161/167 | 161/167 | 161/167 | 167/167 | 167/167 | 167/167 | 161/167 |
| SSR083 | 155/155 | 155/158 | 155/155 | 155/155 | 155/155 | 155/155 | 155/158 | 155/155 | 155/155 | 155/155 | 155/155 | 155/158 | 155/158 | 155/158 | 155/158 | 155/155 | 155/155 |
| SSR084 | 182/182 | 182/182 | 182/182 | 179/182 | 167/182 | 182/182 | 167/182 | 182/182 | 182/182 | 179/179 | 182/182 | 182/182 | 167/182 | 179/182 | 167/167 | 182/182 | 167/167 |
| SSR085 | 142/142 | 142/142 | 144/144 | 142/142 | 142/144 | 142/142 | 142/144 | 142/142 | 142/142 | 144/144 | 142/142 | 142/142 | 142/142 | 142/142 | 144/144 | 142/142 | 142/144 |
| SSR086 | 137/137 | 137/137 | 137/137 | 137/137 | 137/137 | 137/137 | 137/137 | 137/137 | 137/137 | 137/137 | 137/137 | 137/137 | 137/137 | 137/137 | 137/137 | 137/137 | 137/137 |
| SSR087 | 154/154 | No | 146/154 | 146/154 | 146/154 | 154/154 | 146/154 | 146/154 | 154/154 | 146/146 | 154/154 | 146/154 | 146/154 | 146/146 | 154/154 | 154/154 | 146/154 |
| SSR088 | 118/118 | 118/118 | 118/118 | 116/118 | 118/118 | 118/118 | 118/118 | 118/118 | 118/118 | 116/118 | 116/118 | 118/118 | 118/118 | 118/118 | 118/118 | 118/118 | 118/118 |
| SSR089 | 175/175 | 175/175 | 175/175 | 175/175 | 175/175 | 175/175 | 175/175 | 173/175 | 175/175 | 175/175 | 173/175 | 175/175 | 175/175 | 173/175 | 175/175 | 175/175 | 173/175 |
| SSR090 | 140/162 | 140/170 | 140/140 | 140/170 | 140/162 | 140/170 | 140/162 | 140/170 | 140/170 | 140/140 | 140/162 | 140/140 | 140/162 | 164/186 | 162/162 | 162/176 | 140/140 |
| SSR091 | 174/174 | 172/172 | 174/174 | 172/172 | 172/172 | 172/174 | 172/172 | 172/172 | 172/172 | 172/172 | 172/172 | 172/172 | 172/174 | 172/174 | 174/174 | 174/174 | 172/174 |
| SSR092 | 173/173 | 171/171 | 173/173 | 173/173 | 171/171 | 171/171 | 171/171 | 171/171 | 171/171 | 173/173 | 171/173 | 173/173 | 171/171 | 173/173 | 171/171 | 173/173 | 171/173 |
| SSR093 | 188/200 | 176/188 | 178/178 | 188/194 | 176/200 | 176/200 | 176/200 | 176/176 | 176/200 | 176/190 | 176/188 | 188/188 | 188/188 | 194/194 | 188/188 | 188/188 | 186/188 |
| SSR094 | 146/146 | 146/146 | 152/152 | 136/136 | 146/146 | 146/146 | 146/146 | 146/146 | 146/146 | 152/152 | 146/148 | 146/146 | 146/146 | 150/150 | 146/146 | 146/146 | 146/146 |
| SSR095 | None | 134/136 | 136/142 | 136/138 | 136/144 | None | 136/144 | 136/144 | 136/136 | 136/144 | 148/152 | 136/144 | 136/148 | 129/134 | 150/150 | 144/144 | 146/148 |
| SSR096 | 161/161 | 163/163 | 153/153 | 127/143 | 159/161 | 161/163 | 159/161 | 161/163 | 161/163 | 143/165 | 143/163 | 163/163 | 143/163 | 151/151 | 163/163 | 163/163 | 143/163 |
| SSR097 | 113/113 | 113/128 | 113/113 | 113/130 | 113/113 | 113/113 | 113/113 | 113/113 | 113/128 | 130/130 | 113/130 | 113/113 | 113/130 | 113/113 | 113/113 | 113/113 | 113/130 |
| SSR098 | 157/157 | 157/157 | 157/157 | 151/151 | 157/157 | 151/157 | 157/157 | 151/157 | 157/157 | 157/157 | 157/157 | 157/157 | 151/157 | 157/157 | 151/151 | 151/151 | 151/157 |
| SSR099 | 155/155 | 155/155 | 155/155 | 155/172 | 155/155 | 155/155 | 155/155 | 155/155 | 155/155 | 155/180 | 155/155 | 155/155 | 155/155 | 170/170 | 155/155 | 155/155 | 155/155 |
| SSR100 | 155/155 | 155/155 | 155/155 | 155/171 | 155/155 | 155/155 | 155/155 | 155/155 | 155/155 | 155/171 | 155/155 | 155/155 | 155/155 | 181/181 | 155/155 | 155/155 | 155/155 |
| SSR101 | 159/159 | 159/159 | 159/159 | 153/159 | 159/159 | 159/159 | 159/159 | 159/159 | 159/159 | 159/159 | 159/159 | 159/159 | 159/159 | 159/159 | 159/159 | 159/159 | 159/159 |
| SSR102 | 145/145 | 179/216 | 177/177 | 149/216 | 216/216 | 145/220 | 216/216 | 216/222 | None | 149/227 | 145/145 | 145/145 | 145/145 | 177/177 | 145/145 | 145/145 | 145/171 |
| SSR105 | 175/175 | 177/177 | 175/175 | 175/177 | 175/175 | 175/177 | 175/175 | 175/177 | 175/177 | 175/175 | 175/175 | 175/177 | 175/175 | 175/175 | 175/177 | 175/177 | 175/175 |
| SSR106 | 174/174 | 174/174 | 169/174 | 174/174 | 174/174 | 174/174 | 174/174 | 174/174 | 174/174 | 174/174 | 174/174 | 174/174 | 174/174 | 169/169 | 174/174 | 174/174 | 174/174 |
| SSR107 | 171/171 | 205/217 | 171/171 | 173/215 | 171/215 | 171/171 | 171/215 | 171/215 | 215/220 | 171/211 | 171/171 | 171/171 | 171/171 | 197/197 | 171/171 | 171/171 | 171/171 |
| SSR108 | 161/161 | 161/161 | 161/161 | 161/167 | 161/167 | 161/161 | 161/167 | 161/161 | 161/167 | 161/161 | 161/161 | 161/161 | 161/161 | 161/161 | 161/161 | 161/161 | 161/161 |
| SSR109 | 107/107 | 111/111 | 107/107 | 107/111 | 107/111 | 107/111 | 107/111 | 107/111 | 107/111 | 107/107 | 107/111 | 111/111 | 107/111 | 107/107 | 111/111 | 111/111 | 107/111 |
| SSR110 | 146/146 | 148/156 | 144/146 | 105/105 | 105/148 | 146/148 | 105/148 | 148/148 | 105/148 | 140/148 | 146/150 | None | None | 142/146 | 146/148 | 146/146 | 105/150 |
| SSR111 | 118/121 | 118/118 | 118/118 | 118/121 | 118/121 | 118/121 | 118/121 | 118/121 | 118/118 | 118/121 | 118/118 | 121/121 | 118/121 | 118/118 | 121/121 | 121/121 | 118/121 |
| SSR112 | 139/139 | 139/139 | 139/139 | 139/139 | 139/139 | 139/139 | 139/139 | 139/139 | 139/139 | 139/139 | 139/139 | 139/139 | 139/139 | 139/139 | 139/139 | 139/139 | 139/139 |
| SSR113 | 166/172 | 166/166 | 125/125 | 174/174 | 166/172 | 166/172 | 166/172 | 166/172 | 166/166 | 176/176 | 172/176 | 172/172 | 172/172 | 148/164 | 172/172 | 172/172 | 125/172 |
| SSR115 | 147/147 | 121/147 | 147/147 | 143/147 | 147/147 | 147/147 | 147/149 | 147/147 | 121/147 | 149/149 | 147/149 | 147/147 | 147/149 | 141/147 | 147/147 | 147/147 | 149/149 |
| SSR116 | 104/104 | 110/110 | 110/110 | 106/120 | 104/110 | 104/110 | 104/110 | 110/110 | 110/110 | 106/110 | 104/104 | 110/110 | 104/110 | 116/116 | 104/104 | 104/104 | 104/110 |
| SSR118 | 150/150 | 150/150 | 144/150 | 150/150 | 150/150 | 150/150 | 150/150 | 150/150 | 150/150 | 150/150 | 150/150 | 150/150 | 150/150 | 144/150 | 150/150 | 144/150 | 150/150 |
| SSR119 | 137/137 | 135/137 | 133/135 | 133/133 | 133/137 | 137/137 | 133/137 | 135/137 | 137/137 | 135/137 | 133/137 | 133/133 | 133/137 | 133/137 | 137/137 | 133/133 | 133/137 |
| SSR120 | 187/187 | 183/187 | 183/191 | 189/191 | 187/187 | 187/187 | 187/187 | 183/187 | 187/187 | 164/193 | 164/187 | 191/191 | 187/187 | 193/203 | 187/187 | 191/191 | 164/187 |
| SSR121 | 162/162 | 150/150 | 150/150 | 150/150 | 150/150 | 150/150 | 150/150 | 150/150 | 150/150 | 162/162 | 150/150 | 162/162 | None | 150/150 | 150/150 | 150/150 | 162/162 |
| SSR122 | 170/170 | 170/170 | 164/170 | 170/170 | 170/170 | 170/170 | No | 170/170 | 170/170 | 161/161 | 170/170 | 170/170 | 170/170 | 170/170 | 170/170 | 170/170 | 170/170 |
| SSR123 | 167/167 | 163/163 | 167/167 | 161/167 | 163/167 | 167/167 | 163/167 | 161/167 | 163/167 | 161/167 | 161/167 | 167/167 | 167/167 | 171/171 | 167/169 | 167/167 | 167/167 |
| SSR125 | 153/167 | 146/167 | 148/177 | 165/171 | 167/189 | 167/167 | 167/189 | 167/179 | 167/167 | 160/167 | 167/171 | 153/189 | 153/163 | 155/163 | 153/153 | 153/153 | 163/171 |
| SSR127 | 155/168 | 155/155 | 144/144 | 166/166 | 155/155 | None | 155/155 | 155/155 | No | 153/153 | 168/168 | 155/155 | 170/170 | 172/172 | 155/168 | 170/170 | 168/170 |
| SSR128 | 176/176 | 176/178 | 176/178 | 176/178 | 176/176 | 176/177 | 176/178 | 176/179 | 176/180 | 176/181 | 176/182 | 178/178 | 176/178 | 176/176 | 178/178 | 176/178 | 178/178 |
| SSR130 | 133/133 | 133/133 | 133/133 | 133/133 | 133/173 | 133/133 | 133/173 | 133/133 | 133/133 | 133/133 | 133/171 | 173/173 | 133/173 | 133/177 | 171/171 | 133/171 | 171/173 |
| SSR131 | 168/190 | 190/190 | 174/174 | 168/168 | 168/190 | 168/190 | 168/190 | 168/190 | 190/190 | 168/168 | 168/168 | 168/168 | 168/174 | 174/174 | 168/168 | 168/168 | 174/174 |
| SSR132 | 167/175 | 163/167 | 163/163 | 167/175 | 167/175 | 167/175 | 167/175 | 175/175 | 167/175 | 159/167 | 167/167 | 167/167 | 167/175 | 167/167 | 167/167 | 163/163 | 167/175 |
| SSR133 | 137/170 | 137/137 | 164/164 | 135/135 | 137/174 | 137/170 | 137/174 | 137/170 | 137/137 | 137/159 | 135/170 | 170/170 | 168/170 | 178/178 | 170/170 | 170/170 | 168/168 |
| SSR134 | 159/159 | 137/159 | 137/159 | 137/159 | 159/167 | 137/159 | 159/167 | 137/167 | 137/159 | 159/167 | 159/159 | 159/159 | 137/159 | 137/159 | 137/137 | 159/159 | 159/159 |
| SSR135 | 173/173 | 173/173 | 177/177 | 173/173 | 173/177 | 173/173 | 173/177 | 173/173 | 173/173 | 177/177 | 173/242 | 173/173 | 173/177 | None | 177/177 | 173/175 | None |
| SSR136 | 99/101 | 99/101 | 101/101 | 99/101 | 101/101 | 101/101 | 101/101 | 101/101 | 101/101 | 101/101 | 99/101 | 99/99 | 99/99 | 101/101 | 99/99 | 99/99 | 99/101 |
| SSR137 | 168/168 | 154/168 | 166/166 | 168/180 | 166/168 | 168/168 | 166/168 | 166/168 | 168/168 | 168/178 | 178/178 | 166/166 | 166/168 | 160/180 | 168/168 | 166/166 | 166/166 |
| SSR138 | 138/138 | 138/138 | 138/138 | 138/158 | 138/172 | 138/138 | 138/172 | 138/172 | 138/138 | 138/138 | 197/197 | 138/138 | 138/172 | 138/138 | 138/138 | 138/138 | 172/172 |
| SSR139 | 124/138 | 104/124 | 124/130 | 124/124 | None | 124/124 | 124/124 | 124/138 | 124/124 | 124/130 | 136/140 | 124/128 | 124/138 | 148/156 | 138/138 | 128/128 | 138/138 |
| SSR143 | 147/147 | 147/163 | 147/147 | 163/190 | 147/163 | 147/147 | 147/163 | 147/163 | 163/163 | 147/147 | 147/163 | 147/147 | 147/163 | 149/149 | 147/147 | 147/147 | 147/163 |
| SSR144 | 168/168 | 166/166 | 166/166 | 166/191 | 166/166 | 166/168 | 166/166 | 166/166 | 166/166 | 168/195 | 166/168 | 166/166 | 166/193 | 189/189 | 166/166 | 166/166 | 166/193 |
| SSR145 | 249/249 | 249/249 | 249/249 | 249/249 | 249/249 | 249/249 | 249/249 | 249/249 | 249/249 | 249/249 | 249/249 | 249/249 | 249/249 | 249/249 | 249/249 | 249/249 | 249/249 |
| SSR146 | 149/149 | 134/134 | 151/151 | 151/151 | 149/151 | 134/149 | 149/151 | 149/151 | 134/149 | 134/151 | 134/151 | 134/134 | 134/151 | 151/151 | 134/134 | 134/134 | 134/151 |
| SSR147 | 112/112 | 96/108 | 112/122 | 108/108 | 112/112 | 112/112 | 112/112 | 112/112 | 96/112 | 108/108 | 104/112 | 112/112 | 104/112 | 96/96 | 112/112 | 112/112 | 104/112 |
| SSR148 | 149/149 | 149/149 | 149/153 | 153/153 | 149/153 | 149/153 | 149/153 | 149/153 | 149/153 | 153/153 | 149/153 | 149/149 | 149/149 | 153/153 | 149/149 | 149/149 | 149/149 |
| SSR149 | 108/116 | 108/108 | 112/116 | 108/108 | 108/116 | 108/116 | 108/116 | 108/108 | 108/108 | 116/116 | 108/116 | 108/116 | 108/116 | 110/112 | 108/108 | 108/116 | 108/116 |
| SSR150 | 256/256 | 256/256 | 256/256 | 256/256 | 256/258 | 256/256 | 256/258 | 256/256 | 256/256 | 301/301 | 256/301 | 256/256 | 256/256 | 256/256 | 256/256 | 256/256 | 256/301 |
| SSR151 | 342/342 | 309/342 | 309/315 | 315/364 | 309/342 | 309/342 | 309/342 | 309/309 | 309/342 | 313/315 | 309/309 | 308/308 | 308/308 | 313/313 | 308/308 | 308/308 | 308/308 |
| SSR152 | 256/264 | 256/256 | 256/297 | 254/256 | 266/270 | 264/266 | 266/270 | 266/266 | 256/266 | 302/308 | 254/266 | 262/262 | 256/264 | 231/231 | 266/266 | 270/270 | 256/266 |
| SSR153 | 317/319 | 317/319 | 317/319 | 317/319 | 317/319 | 317/317 | 317/319 | 317/319 | 317/317 | 317/319 | 319/319 | 319/319 | 319/319 | 319/319 | 319/319 | 319/319 | 319/319 |
| SSR154 | 242/255 | 255/255 | 240/240 | 245/269 | 255/255 | 242/255 | 255/255 | 234/255 | 255/255 | 244/244 | 242/255 | 255/255 | 242/255 | 255/255 | 242/255 | 234/255 | 242/242 |
| SSR155 | 269/269 | 265/269 | 271/285 | 269/271 | No | No | No | No | 269/269 | 269/269 | 273/275 | No | 269/269 | 269/271 | 267/269 | 269/271 | 269/273 |
| SSR156 | 186/186 | 180/180 | 180/180 | 180/180 | 180/180 | 186/186 | 180/180 | 180/180 | 180/180 | 180/180 | 186/186 | 180/180 | 186/186 | 186/186 | 186/186 | 180/180 | 186/186 |
| SSR157 | 137/137 | 137/137 | 137/137 | 137/137 | 137/137 | 137/137 | 137/137 | 137/137 | 137/137 | 137/137 | 137/137 | 137/137 | 137/137 | 137/137 | 137/137 | 137/137 | 137/137 |
| SSR158 | 266/266 | 266/266 | 154/264 | 266/266 | 266/266 | 266/266 | 266/266 | 266/266 | 266/266 | 266/266 | 266/266 | 266/266 | 266/266 | None | 266/266 | 266/266 | 266/266 |
| SSR159 | 332/332 | 332/332 | 290/290 | 287/290 | 290/290 | 332/332 | 290/290 | 290/332 | 290/332 | 290/290 | 290/332 | 290/332 | 290/332 | 287/287 | 332/332 | 290/290 | 290/290 |
| SSR160 | 216/216 | 171/192 | 171/208 | 171/197 | 171/216 | 171/216 | 171/216 | 171/171 | 171/192 | 171/216 | 171/171 | 171/171 | 171/171 | 171/171 | 171/171 | 171/171 | 171/171 |
| SSR161 | 297/297 | 295/295 | 297/297 | 295/297 | 295/295 | 295/297 | 295/295 | 295/295 | 295/295 | 297/299 | 297/297 | 297/297 | 297/297 | 297/297 | 297/297 | 297/297 | 297/297 |
| SSR162 | 318/318 | 262/318 | 272/339 | 272/274 | 276/318 | 318/318 | 276/318 | 318/318 | 262/316 | 264/280 | 332/332 | 262/262 | None | 276/276 | 332/332 | 262/262 | 332/332 |
| SSR163 | 235/235 | 229/235 | 183/235 | 183/235 | 229/235 | 229/235 | 229/235 | 229/229 | 229/235 | 185/185 | 183/233 | 183/183 | 183/229 | 235/235 | 183/183 | 183/185 | 183/233 |
| SSR169 | 250/250 | 250/288 | 250/284 | 270/282 | 250/288 | 250/288 | 250/288 | 250/288 | 250/288 | 250/286 | 250/250 | 250/250 | 250/250 | 280/280 | 250/250 | 250/250 | 250/250 |
| SSR170 | 203/282 | 203/285 | 285/287 | 278/285 | 203/203 | 203/203 | 203/203 | 203/285 | 203/203 | 200/200 | 203/280 | 203/285 | 203/278 | 200/200 | 203/203 | 285/285 | 280/280 |
| SSR171 | 348/357 | 357/357 | 342/357 | 338/359 | 357/357 | 357/357 | 357/357 | 357/357 | 357/357 | 338/338 | 348/357 | 357/357 | 357/357 | 359/359 | 357/357 | 338/357 | 348/357 |
| SSR172 | 244/244 | 244/244 | 244/244 | 256/256 | 244/244 | 244/244 | 244/244 | 244/244 | 244/244 | 256/258 | 244/244 | 244/256 | 244/244 | 256/258 | 244/244 | 244/256 | 244/244 |
| SSR173 | 163/166 | 163/163 | 163/163 | 166/166 | 163/166 | 163/166 | 163/166 | 163/166 | 163/163 | 163/163 | 163/166 | 163/163 | 163/163 | 163/163 | 166/166 | 163/163 | 163/166 |
| SSR174 | 157/157 | 157/157 | 157/157 | 161/161 | 161/161 | 157/157 | 125/161 | 157/157 | None | 157/157 | 157/157 | 157/157 | 157/157 | None | 157/157 | 157/157 | 157/157 |
| SSR176 | 312/312 | 312/314 | 320/320 | 312/312 | 312/312 | 312/312 | 312/312 | 312/312 | 312/312 | 314/320 | 312/312 | 308/308 | 312/312 | 302/308 | 312/312 | 308/308 | 312/312 |
| SSR177 | 274/274 | 268/268 | 268/268 | 256/266 | 272/274 | 268/274 | 272/274 | 268/274 | 268/274 | 266/268 | 256/268 | 268/268 | 256/268 | 268/268 | 268/268 | 268/268 | 256/268 |
| SSR178 | 175/175 | 122/122 | 191/191 | 155/155 | 122/175 | 122/122 | 122/175 | 122/122 | 122/122 | 189/189 | 175/189 | 122/189 | 189/189 | 189/191 | 175/189 | 189/189 | 189/197 |
| SSR179 | 266/266 | 264/273 | 266/266 | 268/275 | 264/270 | 264/266 | 264/270 | 264/264 | 264/270 | 266/266 | 238/266 | 264/266 | 238/264 | 266/270 | 238/266 | 266/266 | 238/270 |
| SSR180 | 247/247 | 233/247 | 249/249 | 244/249 | 247/247 | 247/247 | 247/247 | 233/247 | 247/247 | 233/249 | 233/233 | 249/249 | 233/247 | 233/244 | 233/233 | 249/249 | 233/247 |
| SSR181 | 246/250 | 204/204 | 255/288 | 204/257 | 204/240 | 255/255 | 204/240 | 238/255 | 204/255 | 208/250 | 206/206 | 240/240 | 238/238 | 206/206 | 204/238 | 228/240 | 238/238 |
| SSR182 | 330/330 | 318/330 | 318/330 | 207/207 | 330/330 | 330/330 | 330/330 | 330/330 | 330/330 | 207/207 | 207/207 | 207/207 | 207/207 | 207/207 | 207/207 | 207/207 | 207/207 |
| SSR183 | 188/235 | 186/188 | 192/192 | 186/194 | 188/188 | 188/235 | 188/188 | 188/235 | 188/188 | 186/186 | 186/235 | 186/186 | 186/233 | 192/192 | 186/186 | 235/235 | 202/233 |
| SSR184 | 251/307 | 251/279 | 253/253 | 253/255 | 251/303 | 251/279 | 251/303 | 251/279 | 251/251 | 253/253 | 279/279 | 279/279 | 279/305 | 253/255 | 305/305 | 303/303 | 255/279 |
| SSR186 | 152/152 | 152/156 | 152/152 | 152/156 | 152/156 | 152/152 | 152/156 | 152/156 | 152/152 | 152/156 | 152/156 | 150/152 | 152/156 | 148/150 | 152/152 | 150/150 | 156/156 |

**S5 Tab. Polymorphism of 15 SSR marker amplifying using 221 peach accessions.**

| Accession | SSR43 | SSR73 | SSR93 | SSR96 | SSR152 | SSR107 | SSR113 | SSR179 | SSR120 | SSR125 | SSR133 | SSR169 | SSR181 | SSR183 | SSR184 |
| --- | --- | --- | --- | --- | --- | --- | --- | --- | --- | --- | --- | --- | --- | --- | --- |
| **1** | 152/202 | 158/158 | 158/170 | 145/147 | 248/252 | 156/156 | 148/154 | 219/219 | 147/169 | 136/152 | 119/153 | 232/232 | 222/224 | 171/219 | 261/261 |
| **2** | 202/202 | 158/170 | 170/176 | 125/145 | 238/238 | 156/156 | 108/154 | 219/249 | 169/185 | 136/172 | 0/0 | 0/0 | 0/0 | 217/219 | 285/285 |
| **3** | 164/188 | 158/158 | 170/170 | 145/145 | 238/252 | 190/202 | 148/154 | 247/249 | 169/169 | 128/150 | 119/153 | 268/268 | 188/224 | 171/171 | 261/261 |
| **4** | 152/164 | 158/188 | 170/172 | 145/149 | 244/248 | 156/202 | 156/158 | 247/251 | 169/171 | 150/158 | 153/157 | 232/268 | 190/224 | 171/171 | 237/287 |
| **5** | 152/188 | 158/158 | 158/170 | 143/145 | 248/248 | 156/156 | 154/154 | 249/249 | 169/169 | 136/150 | 153/153 | 232/270 | 188/238 | 171/219 | 233/287 |
| **6** | 184/204 | 158/158 | 172/172 | 147/147 | 236/238 | 156/156 | 154/156 | 249/249 | 171/171 | 144/162 | 117/155 | 254/268 | 190/190 | 171/171 | 237/285 |
| **7** | 200/200 | 197/197 | 158/158 | 147/147 | 250/250 | 156/156 | 140/156 | 257/259 | 147/169 | 164/164 | 141/141 | 232/232 | 192/192 | 173/173 | 285/285 |
| **8** | 202/212 | 158/188 | 170/170 | 145/145 | 248/248 | 156/156 | 156/156 | 219/219 | 171/171 | 140/144 | 117/141 | 254/254 | 190/236 | 171/171 | 237/237 |
| **9** | 202/202 | 197/197 | 172/172 | 145/145 | 250/250 | 156/156 | 140/140 | 259/259 | 171/171 | 140/140 | 141/141 | 266/266 | 188/188 | 173/173 | 285/285 |
| **10** | 152/152 | 170/170 | 158/158 | 145/145 | 248/248 | 156/156 | 140/140 | 257/257 | 171/171 | 166/166 | 141/141 | 252/252 | 188/188 | 173/173 | 285/285 |
| **11** | 152/152 | 197/197 | 172/172 | 145/145 | 226/226 | 156/156 | 140/140 | 257/257 | 147/147 | 150/150 | 141/141 | 254/254 | 188/188 | 173/173 | 285/285 |
| **12** | 210/210 | 188/188 | 170/170 | 125/145 | 248/292 | 200/200 | 156/156 | 219/219 | 171/171 | 140/140 | 117/117 | 254/268 | 190/190 | 171/171 | 237/237 |
| **13** | 152/188 | 173/197 | 158/158 | 141/143 | 238/256 | 170/202 | 148/156 | 247/247 | 169/173 | 128/158 | 119/153 | 232/270 | 188/188 | 173/173 | 233/235 |
| **14** | 188/188 | 158/197 | 158/158 | 143/143 | 238/254 | 170/200 | 154/156 | 247/249 | 147/173 | 128/150 | 153/153 | 268/270 | 188/222 | 173/219 | 235/261 |
| **15** | 152/188 | 173/197 | 158/158 | 141/141 | 238/256 | 170/202 | 148/156 | 247/247 | 169/173 | 128/156 | 119/153 | 232/270 | 188/188 | 173/173 | 233/235 |
| **16** | 152/188 | 173/197 | 158/158 | 141/143 | 238/256 | 170/224 | 148/156 | 247/247 | 169/173 | 128/156 | 119/153 | 232/270 | 188/188 | 171/173 | 233/235 |
| **17** | 152/152 | 188/188 | 172/172 | 133/143 | 226/248 | 198/200 | 130/130 | 247/247 | 147/171 | 162/162 | 153/153 | 232/268 | 192/224 | 171/179 | 285/285 |
| **18** | 188/202 | 188/197 | 170/182 | 145/147 | 248/252 | 156/200 | 148/154 | 219/253 | 147/169 | 136/150 | 119/153 | 268/270 | 188/224 | 173/219 | 235/285 |
| **19** | 152/152 | 158/158 | 170/170 | 145/145 | 248/252 | 156/156 | 154/154 | 249/249 | 169/173 | 136/136 | 153/153 | 232/232 | 188/222 | 171/219 | 285/287 |
| **20** | 152/152 | 158/158 | 158/170 | 145/145 | 248/252 | 156/156 | 154/154 | 249/249 | 169/173 | 136/150 | 153/153 | 232/270 | 212/238 | 171/221 | 233/285 |
| **21** | 164/192 | 158/170 | 158/172 | 145/147 | 248/290 | 156/202 | 154/158 | 247/249 | 165/175 | 150/150 | 119/141 | 232/268 | 224/234 | 171/171 | 235/285 |
| **22** | 152/202 | 158/158 | 158/170 | 145/147 | 248/252 | 156/156 | 154/154 | 249/249 | 147/171 | 136/152 | 153/153 | 232/254 | 224/224 | 217/219 | 261/287 |
| **23** | 152/202 | 158/158 | 170/170 | 145/145 | 244/248 | 156/156 | 148/154 | 249/249 | 147/169 | 136/154 | 119/153 | 232/254 | 222/224 | 217/221 | 261/287 |
| **24** | 152/152 | 197/197 | 158/158 | 131/131 | 246/246 | 156/156 | 140/140 | 259/259 | 147/147 | 162/162 | 141/141 | 252/252 | 188/188 | 173/173 | 285/285 |
| **25** | 186/210 | 158/188 | 172/172 | 125/163 | 226/248 | 156/204 | 150/156 | 219/249 | 147/171 | 152/176 | 117/117 | 252/264 | 188/236 | 171/217 | 235/237 |
| **26** | 164/202 | 158/188 | 170/170 | 145/145 | 238/248 | 156/156 | 154/158 | 219/249 | 147/169 | 136/156 | 153/157 | 232/232 | 190/224 | 171/219 | 261/261 |
| **27** | 164/188 | 158/158 | 170/170 | 145/145 | 238/252 | 202/204 | 154/154 | 247/249 | 169/169 | 130/150 | 119/153 | 232/268 | 188/224 | 171/219 | 261/287 |
| **28** | 164/188 | 158/158 | 170/170 | 145/145 | 238/252 | 202/202 | 154/154 | 247/247 | 165/169 | 128/150 | 153/153 | 232/268 | 222/224 | 171/219 | 287/287 |
| **29** | 188/202 | 158/197 | 158/158 | 143/143 | 248/248 | 156/200 | 154/154 | 249/249 | 165/165 | 128/162 | 153/153 | 232/232 | 188/222 | 173/219 | 261/261 |
| **30** | 188/188 | 197/197 | 158/170 | 143/145 | 238/248 | 190/202 | 148/148 | 247/253 | 165/169 | 130/130 | 119/119 | 232/232 | 188/188 | 173/173 | 233/261 |
| **31** | 202/202 | 158/158 | 170/170 | 141/145 | 252/252 | 156/156 | 154/154 | 219/249 | 147/147 | 136/136 | 153/153 | 266/266 | 190/190 | 219/219 | 283/283 |
| **32** | 152/152 | 158/197 | 170/170 | 145/145 | 248/248 | 156/156 | 148/158 | 219/255 | 169/173 | 154/154 | 119/157 | 232/232 | 222/222 | 173/217 | 261/285 |
| **33** | 152/152 | 152/158 | 170/170 | 145/145 | 244/252 | 156/156 | 148/158 | 219/255 | 169/173 | 154/154 | 119/157 | 232/232 | 190/222 | 171/219 | 261/261 |
| **34** | 152/164 | 158/197 | 170/170 | 145/145 | 238/248 | 156/190 | 148/158 | 219/255 | 169/173 | 150/154 | 119/157 | 232/232 | 188/222 | 173/217 | 261/285 |
| **35** | 188/188 | 197/197 | 170/182 | 145/147 | 248/248 | 190/204 | 148/156 | 247/247 | 165/169 | 128/150 | 117/119 | 232/270 | 188/238 | 173/217 | 233/261 |
| **36** | 0/0 | 170/170 | 176/176 | 125/125 | 244/244 | 0/0 | 160/160 | 257/257 | 0/0 | 136/136 | 117/117 | 254/254 | 188/190 | 219/219 | 235/263 |
| **37** | 152/202 | 158/158 | 170/170 | 145/145 | 244/244 | 156/156 | 154/154 | 247/249 | 173/173 | 136/172 | 153/153 | 232/232 | 224/224 | 171/171 | 261/261 |
| **38** | 152/164 | 158/188 | 158/170 | 143/145 | 238/248 | 202/202 | 154/154 | 219/249 | 169/173 | 136/150 | 117/153 | 268/268 | 188/224 | 171/171 | 261/285 |
| **39** | 164/184 | 158/158 | 170/172 | 145/147 | 238/238 | 156/202 | 154/156 | 247/249 | 169/171 | 150/162 | 117/153 | 268/268 | 188/190 | 171/171 | 285/287 |
| **40** | 164/188 | 158/158 | 170/170 | 145/145 | 238/252 | 202/202 | 148/154 | 247/249 | 165/169 | 128/150 | 119/153 | 232/268 | 188/224 | 171/171 | 261/287 |
| **41** | 152/164 | 158/158 | 158/170 | 145/145 | 238/248 | 190/202 | 154/156 | 219/247 | 169/171 | 136/150 | 153/155 | 232/268 | 222/224 | 171/171 | 261/261 |
| **42** | 152/202 | 158/188 | 158/172 | 145/149 | 244/248 | 156/156 | 148/156 | 249/251 | 147/171 | 136/160 | 119/153 | 232/268 | 190/224 | 171/171 | 237/261 |
| **43** | 152/202 | 158/158 | 158/170 | 145/145 | 238/256 | 156/156 | 154/154 | 219/219 | 147/169 | 136/154 | 153/155 | 232/268 | 188/222 | 171/221 | 261/285 |
| **44** | 152/152 | 158/158 | 170/170 | 131/131 | 248/248 | 156/156 | 154/154 | 219/219 | 147/147 | 136/136 | 155/155 | 268/268 | 222/224 | 171/171 | 285/285 |
| **45** | 152/152 | 158/188 | 170/172 | 145/149 | 244/248 | 156/156 | 154/156 | 249/251 | 169/171 | 154/158 | 153/153 | 268/268 | 188/190 | 171/171 | 237/261 |
| **46** | 152/152 | 158/158 | 158/158 | 145/147 | 244/248 | 156/156 | 152/154 | 219/249 | 147/171 | 154/164 | 117/153 | 232/268 | 222/224 | 219/221 | 287/287 |
| **47** | 152/202 | 158/188 | 170/172 | 145/145 | 238/244 | 156/156 | 154/156 | 249/249 | 147/169 | 136/154 | 153/153 | 254/268 | 222/226 | 171/171 | 233/237 |
| **48** | 152/152 | 188/188 | 158/170 | 145/145 | 248/248 | 156/202 | 154/154 | 219/253 | 173/173 | 136/154 | 117/155 | 232/268 | 222/224 | 171/171 | 261/285 |
| **49** | 152/164 | 0/0 | 0/0 | 145/145 | 248/248 | 156/156 | 152/154 | 219/219 | 171/173 | 150/154 | 117/153 | 232/268 | 222/224 | 0/0 | 261/285 |
| **50** | 152/164 | 158/188 | 158/170 | 145/145 | 238/248 | 156/202 | 152/154 | 219/247 | 165/171 | 150/154 | 117/119 | 232/268 | 222/224 | 171/171 | 261/285 |
| **51** | 152/152 | 188/188 | 158/170 | 145/145 | 248/248 | 156/156 | 152/152 | 219/219 | 173/173 | 152/152 | 117/117 | 232/232 | 190/224 | 171/171 | 289/289 |
| **52** | 200/204 | 170/188 | 172/176 | 127/127 | 226/226 | 156/156 | 108/108 | 219/251 | 147/147 | 140/154 | 153/153 | 252/252 | 188/188 | 179/179 | 237/237 |
| **53** | 200/200 | 197/197 | 158/158 | 141/141 | 226/226 | 156/200 | 156/156 | 259/259 | 147/147 | 146/146 | 141/141 | 232/232 | 188/188 | 171/173 | 285/285 |
| **54** | 152/152 | 158/188 | 170/172 | 149/149 | 248/248 | 156/156 | 154/156 | 219/219 | 171/171 | 146/146 | 117/117 | 254/254 | 222/224 | 171/171 | 237/237 |
| **55** | 206/206 | 197/197 | 170/170 | 145/145 | 248/248 | 156/156 | 156/156 | 219/219 | 171/171 | 142/142 | 155/155 | 254/254 | 190/190 | 179/179 | 237/237 |
| **56** | 188/210 | 158/158 | 170/170 | 145/145 | 226/248 | 156/156 | 108/108 | 219/219 | 171/171 | 140/140 | 117/141 | 254/254 | 234/248 | 171/171 | 235/235 |
| **57** | 152/188 | 188/197 | 158/168 | 143/145 | 238/248 | 156/202 | 154/156 | 247/253 | 147/173 | 128/154 | 119/151 | 232/232 | 188/222 | 173/217 | 233/237 |
| **58** | 188/206 | 158/197 | 158/174 | 145/161 | 248/256 | 156/204 | 108/148 | 219/247 | 147/169 | 150/168 | 119/147 | 232/270 | 188/232 | 171/173 | 233/235 |
| **59** | 188/202 | 158/197 | 158/170 | 141/145 | 252/256 | 156/170 | 154/156 | 247/249 | 147/173 | 128/136 | 153/153 | 232/268 | 188/224 | 173/219 | 235/285 |
| **60** | 186/186 | 197/197 | 158/182 | 143/145 | 238/248 | 200/204 | 148/156 | 247/255 | 169/169 | 128/150 | 119/153 | 232/270 | 188/238 | 173/173 | 233/235 |
| **61** | 152/194 | 158/188 | 170/170 | 145/145 | 238/248 | 156/156 | 148/154 | 249/251 | 171/171 | 150/154 | 119/153 | 232/232 | 190/222 | 217/219 | 261/285 |
| **62** | 152/194 | 158/188 | 170/170 | 145/145 | 238/248 | 156/156 | 148/154 | 249/251 | 171/171 | 150/154 | 119/153 | 232/232 | 190/222 | 217/219 | 261/285 |
| **63** | 188/202 | 188/197 | 170/182 | 145/147 | 248/252 | 156/200 | 148/154 | 219/253 | 147/169 | 136/150 | 119/153 | 268/270 | 188/224 | 173/219 | 235/285 |
| **64** | 164/188 | 158/197 | 158/158 | 145/145 | 238/248 | 190/202 | 148/156 | 247/255 | 165/169 | 150/150 | 119/153 | 232/270 | 188/238 | 173/173 | 233/233 |
| **65** | 152/188 | 158/158 | 170/170 | 145/147 | 248/248 | 156/156 | 154/154 | 247/247 | 147/169 | 128/136 | 153/157 | 232/232 | 190/238 | 171/171 | 261/287 |
| **66** | 152/152 | 158/191 | 158/168 | 125/153 | 236/238 | 156/190 | 108/158 | 219/249 | 147/173 | 138/154 | 117/151 | 232/266 | 190/222 | 171/187 | 237/261 |
| **67** | 184/188 | 158/188 | 170/170 | 125/145 | 238/248 | 156/156 | 154/156 | 247/249 | 147/169 | 128/146 | 153/157 | 232/232 | 188/226 | 171/217 | 285/287 |
| **68** | 164/188 | 170/197 | 160/176 | 147/169 | 236/256 | 156/196 | 118/156 | 251/257 | 171/173 | 150/168 | 117/147 | 232/252 | 188/236 | 173/217 | 235/285 |
| **69** | 152/206 | 188/197 | 158/176 | 125/167 | 236/290 | 156/204 | 154/156 | 249/253 | 147/171 | 138/138 | 117/153 | 252/266 | 190/234 | 171/217 | 235/235 |
| **70** | 152/184 | 158/188 | 170/170 | 125/145 | 238/246 | 156/156 | 154/154 | 219/247 | 169/169 | 136/146 | 151/153 | 232/232 | 222/222 | 171/217 | 261/287 |
| **71** | 152/152 | 158/158 | 170/170 | 145/145 | 248/250 | 156/156 | 154/154 | 219/249 | 169/173 | 136/136 | 153/153 | 232/270 | 188/224 | 219/219 | 233/285 |
| **72** | 152/190 | 188/197 | 170/170 | 125/145 | 238/252 | 156/156 | 154/154 | 219/247 | 169/173 | 136/172 | 151/153 | 232/232 | 222/224 | 173/217 | 285/287 |
| **73** | 152/182 | 158/191 | 168/170 | 145/145 | 238/248 | 156/156 | 154/154 | 219/253 | 169/169 | 136/146 | 151/153 | 232/232 | 188/222 | 187/219 | 261/287 |
| **74** | 152/188 | 158/158 | 158/170 | 143/145 | 248/248 | 156/156 | 154/154 | 249/249 | 169/169 | 136/150 | 153/153 | 232/270 | 188/238 | 171/219 | 233/287 |
| **75** | 152/188 | 158/197 | 170/182 | 143/145 | 248/252 | 156/156 | 148/154 | 247/249 | 169/173 | 136/150 | 119/153 | 232/270 | 224/238 | 173/219 | 261/285 |
| **76** | 152/206 | 158/158 | 170/170 | 143/145 | 248/248 | 156/156 | 154/154 | 249/255 | 147/169 | 136/146 | 153/153 | 232/232 | 188/222 | 171/171 | 285/287 |
| **77** | 152/188 | 158/158 | 158/170 | 143/145 | 246/248 | 156/156 | 154/154 | 249/249 | 169/169 | 136/150 | 153/153 | 232/270 | 188/238 | 171/219 | 233/287 |
| **78** | 176/182 | 176/176 | 176/176 | 131/133 | 214/214 | 182/182 | 130/146 | 249/253 | 175/185 | 138/146 | 161/161 | 262/262 | 190/190 | 177/177 | 235/237 |
| **79** | 188/188 | 158/197 | 182/182 | 143/145 | 246/248 | 156/156 | 148/154 | 249/249 | 169/169 | 150/150 | 119/153 | 232/270 | 224/238 | 173/219 | 233/289 |
| **80** | 188/204 | 158/158 | 170/182 | 145/145 | 248/248 | 156/156 | 154/154 | 249/249 | 147/169 | 136/150 | 153/153 | 232/270 | 224/238 | 217/219 | 233/287 |
| **81** | 164/184 | 158/188 | 170/170 | 143/145 | 244/248 | 156/156 | 154/154 | 219/247 | 169/173 | 146/150 | 151/153 | 232/232 | 222/222 | 217/219 | 261/285 |
| **82** | 152/190 | 158/197 | 170/182 | 143/145 | 248/248 | 156/156 | 148/154 | 219/247 | 169/169 | 136/136 | 119/153 | 232/270 | 188/238 | 171/173 | 233/287 |
| **83** | 152/152 | 158/158 | 170/170 | 143/145 | 246/252 | 156/156 | 148/154 | 247/249 | 169/173 | 136/136 | 153/153 | 232/232 | 212/238 | 171/219 | 233/285 |
| **84** | 152/152 | 158/158 | 170/170 | 145/145 | 248/248 | 156/156 | 154/154 | 219/249 | 169/169 | 136/136 | 153/153 | 232/232 | 188/222 | 171/171 | 287/287 |
| **85** | 152/152 | 158/158 | 170/170 | 145/145 | 252/252 | 156/156 | 154/154 | 249/249 | 173/173 | 136/136 | 153/153 | 232/232 | 212/224 | 219/219 | 285/285 |
| **86** | 152/164 | 158/158 | 170/170 | 145/145 | 238/248 | 156/190 | 154/154 | 219/249 | 147/169 | 136/136 | 157/157 | 232/232 | 190/190 | 171/219 | 285/285 |
| **87** | 152/202 | 158/158 | 170/170 | 145/145 | 238/238 | 156/156 | 154/154 | 219/249 | 169/169 | 136/162 | 153/153 | 232/268 | 222/224 | 217/219 | 261/287 |
| **88** | 152/152 | 158/158 | 170/170 | 145/145 | 248/248 | 156/156 | 154/154 | 219/249 | 169/169 | 136/136 | 153/153 | 232/232 | 188/222 | 171/171 | 287/287 |
| **89** | 152/152 | 158/158 | 170/170 | 145/145 | 238/246 | 156/156 | 154/154 | 219/255 | 147/173 | 136/144 | 153/157 | 232/232 | 222/224 | 219/219 | 261/285 |
| **90** | 152/164 | 158/158 | 170/170 | 145/145 | 248/248 | 156/156 | 154/154 | 219/255 | 147/173 | 150/154 | 153/153 | 232/232 | 222/222 | 171/219 | 261/285 |
| **91** | 152/152 | 158/188 | 170/170 | 143/145 | 244/248 | 156/156 | 154/154 | 219/249 | 147/171 | 136/136 | 153/157 | 232/254 | 188/224 | 171/219 | 261/285 |
| **92** | 152/152 | 158/197 | 170/170 | 143/145 | 246/252 | 156/156 | 148/154 | 249/249 | 169/173 | 136/136 | 119/153 | 232/232 | 212/222 | 173/219 | 233/285 |
| **93** | 152/206 | 158/197 | 170/170 | 145/145 | 238/246 | 156/156 | 148/154 | 255/255 | 147/169 | 146/154 | 119/153 | 232/232 | 222/222 | 173/219 | 261/285 |
| **94** | 152/152 | 158/188 | 170/170 | 145/147 | 238/248 | 156/156 | 154/154 | 219/219 | 147/173 | 136/154 | 153/157 | 232/232 | 188/188 | 217/219 | 285/285 |
| **95** | 152/164 | 158/158 | 170/170 | 145/145 | 248/248 | 156/156 | 154/154 | 219/255 | 147/173 | 150/154 | 153/153 | 232/232 | 222/222 | 171/219 | 261/285 |
| **96** | 152/164 | 158/158 | 170/170 | 145/145 | 248/250 | 156/156 | 154/154 | 219/255 | 147/173 | 150/154 | 153/153 | 232/232 | 222/222 | 171/219 | 261/285 |
| **97** | 152/152 | 158/158 | 170/170 | 145/145 | 246/246 | 156/156 | 148/154 | 219/219 | 169/169 | 146/146 | 119/153 | 232/232 | 222/224 | 171/219 | 237/261 |
| **98** | 152/152 | 158/158 | 170/170 | 145/145 | 250/252 | 156/156 | 154/154 | 219/249 | 173/173 | 136/136 | 153/153 | 232/232 | 222/224 | 219/219 | 285/285 |
| **99** | 152/184 | 188/191 | 168/170 | 125/145 | 238/248 | 156/156 | 108/154 | 219/253 | 147/169 | 146/154 | 151/151 | 232/232 | 222/222 | 187/219 | 237/261 |
| **100** | 152/206 | 158/197 | 170/170 | 145/145 | 238/248 | 156/156 | 148/154 | 255/255 | 147/169 | 144/156 | 119/153 | 232/232 | 222/224 | 173/219 | 261/285 |
| **101** | 152/164 | 158/188 | 158/170 | 145/145 | 238/248 | 156/156 | 154/154 | 219/219 | 147/169 | 136/150 | 153/157 | 232/232 | 224/224 | 217/219 | 261/285 |
| **102** | 152/164 | 158/158 | 170/170 | 145/147 | 246/252 | 156/156 | 148/154 | 219/249 | 147/163 | 146/146 | 119/153 | 232/232 | 224/224 | 171/219 | 285/285 |
| **103** | 152/206 | 158/197 | 170/170 | 145/145 | 238/246 | 156/156 | 148/154 | 255/255 | 147/169 | 146/154 | 119/153 | 232/232 | 222/222 | 173/219 | 261/285 |
| **104** | 152/164 | 158/158 | 170/170 | 145/145 | 248/248 | 156/156 | 154/154 | 219/255 | 147/173 | 150/154 | 153/153 | 232/232 | 222/222 | 171/219 | 261/285 |
| **105** | 188/202 | 158/197 | 170/182 | 143/143 | 238/246 | 156/156 | 148/154 | 249/249 | 169/169 | 136/150 | 119/153 | 232/232 | 224/238 | 173/219 | 233/289 |
| **106** | 152/152 | 158/197 | 170/170 | 143/145 | 246/252 | 156/156 | 148/154 | 249/249 | 169/173 | 136/136 | 119/153 | 232/232 | 212/222 | 173/219 | 233/285 |
| **107** | 188/202 | 158/158 | 170/182 | 143/143 | 238/246 | 156/156 | 154/156 | 249/249 | 169/169 | 150/150 | 153/153 | 232/232 | 224/238 | 219/219 | 233/289 |
| **108** | 164/164 | 158/197 | 158/158 | 145/145 | 248/248 | 156/200 | 148/154 | 247/247 | 169/169 | 150/162 | 119/153 | 232/270 | 238/238 | 173/219 | 261/261 |
| **109** | 152/164 | 158/158 | 170/170 | 145/145 | 238/252 | 156/202 | 154/154 | 249/255 | 147/165 | 150/172 | 119/153 | 232/268 | 188/224 | 171/171 | 233/285 |
| **110** | 164/188 | 158/197 | 158/170 | 145/145 | 238/238 | 190/202 | 148/148 | 247/255 | 165/169 | 128/150 | 119/119 | 232/270 | 188/188 | 171/173 | 233/261 |
| **111** | 188/202 | 197/197 | 170/182 | 143/145 | 246/248 | 156/202 | 148/148 | 247/249 | 169/169 | 136/150 | 119/119 | 232/270 | 238/238 | 173/173 | 233/289 |
| **112** | 186/206 | 158/197 | 158/172 | 125/149 | 236/292 | 194/206 | 156/158 | 249/249 | 147/147 | 138/144 | 143/147 | 232/252 | 188/194 | 171/179 | 235/235 |
| **113** | 176/176 | 197/197 | 160/160 | 127/127 | 276/276 | 194/194 | 150/150 | 249/249 | 173/173 | 138/138 | 151/151 | 232/232 | 188/188 | 171/171 | 237/237 |
| **114** | 180/188 | 170/170 | 160/160 | 135/135 | 238/278 | 156/156 | 108/108 | 249/249 | 165/173 | 130/160 | 147/147 | 232/266 | 0/0 | 177/177 | 235/235 |
| **115** | 204/210 | 188/188 | 170/176 | 109/125 | 236/238 | 156/198 | 156/156 | 251/257 | 171/173 | 148/154 | 117/117 | 252/264 | 188/240 | 171/179 | 235/237 |
| **116** | 206/206 | 158/158 | 158/172 | 163/169 | 236/236 | 156/194 | 148/158 | 249/249 | 147/147 | 138/138 | 141/141 | 252/264 | 194/242 | 171/171 | 235/237 |
| **117** | 152/194 | 170/197 | 170/176 | 123/147 | 236/290 | 156/198 | 154/156 | 249/253 | 173/173 | 146/168 | 117/153 | 252/252 | 188/222 | 217/217 | 235/237 |
| **118** | 204/210 | 188/188 | 170/176 | 109/125 | 236/238 | 158/200 | 156/156 | 251/257 | 171/173 | 148/154 | 117/117 | 252/264 | 188/240 | 171/179 | 235/237 |
| **119** | 214/224 | 188/188 | 172/172 | 125/125 | 266/266 | 156/156 | 150/150 | 251/251 | 171/171 | 166/170 | 153/153 | 252/252 | 192/236 | 219/219 | 237/239 |
| **120** | 152/186 | 188/197 | 172/172 | 143/145 | 226/246 | 156/156 | 108/130 | 247/247 | 147/171 | 164/164 | 141/153 | 268/268 | 190/192 | 171/171 | 285/285 |
| **121** | 200/206 | 158/158 | 158/158 | 161/165 | 290/290 | 156/196 | 154/154 | 249/249 | 175/175 | 138/138 | 153/153 | 232/232 | 188/234 | 171/171 | 235/235 |
| **122** | 164/188 | 158/158 | 158/158 | 145/145 | 238/238 | 190/202 | 112/140 | 247/247 | 169/171 | 150/150 | 119/119 | 232/232 | 188/194 | 171/171 | 233/261 |
| **123** | 152/152 | 158/158 | 176/176 | 141/141 | 244/244 | 194/196 | 146/148 | 257/257 | 173/173 | 156/160 | 117/117 | 254/254 | 190/190 | 171/171 | 237/237 |
| **124** | 152/152 | 158/158 | 176/176 | 141/141 | 244/246 | 194/196 | 146/148 | 257/257 | 171/173 | 156/160 | 117/155 | 254/254 | 188/190 | 171/217 | 237/237 |
| **125** | 186/188 | 158/197 | 170/170 | 145/145 | 236/248 | 156/156 | 108/156 | 219/219 | 171/171 | 160/164 | 153/153 | 254/254 | 0/0 | 171/179 | 237/237 |
| **126** | 204/204 | 197/197 | 172/172 | 135/143 | 226/226 | 194/200 | 108/156 | 247/247 | 147/147 | 162/166 | 155/155 | 268/268 | 192/192 | 171/171 | 285/287 |
| **127** | 186/186 | 158/188 | 172/172 | 127/127 | 292/292 | 156/156 | 108/108 | 219/219 | 147/147 | 168/168 | 117/117 | 232/252 | 224/224 | 179/179 | 237/237 |
| **128** | 152/204 | 158/197 | 170/172 | 143/143 | 226/236 | 156/156 | 108/108 | 219/219 | 147/171 | 166/166 | 153/155 | 252/252 | 192/224 | 171/171 | 237/237 |
| **129** | 152/152 | 197/197 | 172/172 | 135/143 | 226/226 | 0/0 | 108/108 | 247/247 | 0/0 | 142/164 | 155/155 | 268/268 | 192/226 | 179/179 | 285/285 |
| **130** | 164/164 | 176/176 | 160/160 | 135/135 | 282/282 | 156/156 | 146/146 | 249/249 | 165/165 | 152/152 | 123/123 | 232/232 | 188/188 | 171/171 | 237/237 |
| **131** | 152/152 | 197/197 | 172/172 | 131/131 | 248/248 | 156/156 | 140/140 | 259/259 | 147/147 | 164/164 | 141/141 | 252/252 | 188/188 | 173/173 | 285/285 |
| **132** | 0/0 | 170/170 | 170/170 | 125/127 | 248/248 | 0/0 | 108/128 | 251/251 | 0/0 | 150/150 | 117/117 | 232/232 | 188/188 | 179/181 | 235/235 |
| **133** | 188/194 | 170/197 | 170/176 | 139/147 | 226/236 | 194/198 | 156/156 | 249/257 | 171/173 | 148/166 | 117/117 | 0/0 | 0/0 | 173/217 | 235/235 |
| **134** | 164/202 | 158/158 | 158/170 | 143/143 | 248/252 | 190/200 | 154/154 | 249/255 | 165/169 | 136/150 | 153/153 | 268/270 | 188/224 | 219/219 | 261/285 |
| **135** | 202/202 | 158/197 | 158/170 | 145/145 | 248/252 | 156/204 | 154/154 | 247/249 | 165/169 | 136/136 | 153/153 | 270/270 | 224/224 | 219/219 | 233/261 |
| **136** | 202/202 | 158/197 | 170/182 | 143/143 | 238/248 | 200/202 | 154/154 | 249/255 | 165/165 | 136/150 | 153/153 | 232/268 | 188/224 | 219/219 | 233/261 |
| **137** | 188/202 | 158/197 | 158/170 | 143/145 | 238/252 | 156/200 | 154/154 | 247/255 | 169/169 | 136/150 | 153/157 | 232/270 | 188/238 | 173/219 | 233/261 |
| **138** | 152/164 | 197/197 | 158/170 | 143/143 | 248/248 | 156/200 | 154/154 | 249/255 | 147/169 | 150/154 | 119/153 | 232/232 | 188/224 | 173/173 | 261/261 |
| **139** | 164/202 | 197/197 | 158/158 | 143/145 | 248/252 | 190/204 | 148/154 | 247/249 | 165/169 | 136/150 | 119/153 | 270/270 | 188/224 | 173/219 | 233/285 |
| **140** | 164/164 | 158/197 | 170/182 | 143/145 | 248/254 | 156/206 | 148/154 | 247/247 | 169/169 | 150/150 | 119/153 | 232/270 | 188/224 | 173/219 | 233/285 |
| **141** | 164/202 | 158/197 | 170/182 | 143/145 | 248/252 | 200/202 | 148/154 | 247/249 | 169/169 | 136/150 | 119/153 | 232/270 | 188/188 | 173/219 | 233/285 |
| **142** | 152/152 | 158/158 | 172/172 | 123/123 | 226/226 | 200/200 | 130/130 | 247/247 | 147/147 | 164/164 | 153/153 | 252/252 | 224/224 | 171/171 | 237/237 |
| **143** | 188/202 | 197/197 | 170/182 | 143/145 | 238/252 | 200/200 | 148/154 | 247/253 | 169/169 | 136/150 | 119/153 | 232/232 | 224/238 | 173/173 | 233/261 |
| **144** | 152/202 | 188/188 | 170/170 | 145/145 | 252/252 | 156/156 | 154/154 | 249/249 | 147/147 | 136/174 | 157/157 | 268/268 | 224/224 | 219/219 | 285/285 |
| **145** | 164/202 | 197/197 | 170/182 | 143/143 | 248/252 | 190/198 | 148/154 | 247/251 | 165/173 | 136/150 | 119/153 | 232/268 | 188/224 | 173/173 | 233/285 |
| **146** | 152/152 | 158/197 | 170/170 | 143/145 | 246/252 | 156/156 | 148/154 | 249/251 | 169/173 | 136/136 | 119/153 | 232/232 | 212/222 | 173/219 | 233/285 |
| **147** | 164/202 | 158/197 | 158/170 | 143/145 | 248/252 | 156/200 | 148/154 | 247/249 | 147/165 | 136/162 | 119/153 | 268/270 | 222/224 | 173/219 | 233/285 |
| **148** | 164/202 | 197/197 | 170/182 | 143/143 | 248/252 | 190/200 | 148/154 | 247/251 | 165/173 | 136/150 | 119/153 | 232/268 | 188/224 | 173/173 | 233/285 |
| **149** | 152/152 | 158/197 | 170/170 | 143/145 | 246/252 | 156/156 | 148/154 | 249/249 | 169/173 | 136/136 | 119/153 | 232/232 | 212/222 | 173/219 | 233/285 |
| **150** | 164/202 | 197/197 | 170/182 | 143/143 | 248/252 | 190/200 | 148/154 | 247/251 | 165/173 | 136/150 | 119/153 | 232/268 | 188/224 | 173/173 | 233/285 |
| **151** | 164/188 | 197/197 | 158/182 | 141/143 | 248/252 | 156/200 | 148/154 | 247/253 | 169/169 | 150/172 | 119/157 | 232/270 | 188/224 | 173/173 | 233/285 |
| **152** | 164/202 | 158/197 | 170/170 | 145/145 | 238/254 | 156/202 | 148/154 | 247/249 | 147/169 | 136/150 | 119/153 | 232/268 | 188/224 | 173/219 | 261/285 |
| **153** | 152/188 | 197/197 | 158/158 | 143/145 | 248/252 | 156/200 | 148/154 | 247/253 | 169/169 | 150/172 | 119/157 | 232/270 | 188/188 | 173/173 | 233/261 |
| **154** | 152/164 | 197/197 | 158/158 | 143/145 | 248/252 | 200/204 | 154/154 | 247/253 | 169/169 | 150/172 | 153/157 | 232/270 | 188/224 | 173/219 | 233/233 |
| **155** | 164/188 | 158/197 | 158/158 | 143/143 | 248/248 | 200/200 | 138/148 | 247/247 | 169/169 | 150/150 | 119/153 | 232/232 | 238/238 | 173/219 | 233/261 |
| **156** | 164/188 | 158/197 | 170/170 | 143/145 | 238/238 | 156/202 | 148/154 | 249/253 | 165/169 | 128/150 | 119/119 | 232/268 | 188/224 | 171/173 | 261/285 |
| **157** | 188/188 | 158/197 | 158/182 | 143/145 | 246/248 | 156/156 | 148/154 | 247/249 | 169/169 | 150/150 | 119/153 | 232/270 | 238/238 | 173/219 | 233/261 |
| **158** | 164/188 | 197/197 | 170/170 | 145/145 | 248/248 | 190/190 | 154/154 | 247/247 | 165/171 | 128/150 | 153/153 | 268/270 | 188/188 | 173/173 | 261/261 |
| **159** | 188/194 | 197/197 | 182/182 | 143/145 | 238/248 | 156/190 | 148/148 | 247/255 | 165/165 | 128/152 | 119/119 | 232/270 | 188/238 | 173/173 | 235/235 |
| **160** | 164/188 | 197/197 | 158/182 | 141/143 | 248/252 | 156/200 | 148/154 | 247/253 | 169/169 | 150/172 | 119/157 | 232/270 | 188/224 | 173/173 | 233/285 |
| **161** | 152/188 | 197/197 | 158/170 | 141/145 | 248/252 | 200/200 | 148/154 | 247/253 | 169/169 | 150/170 | 119/159 | 232/232 | 188/238 | 173/173 | 233/261 |
| **162** | 188/188 | 197/197 | 158/158 | 141/143 | 248/248 | 156/200 | 148/154 | 247/253 | 169/169 | 150/172 | 119/157 | 232/270 | 188/238 | 173/173 | 233/285 |
| **163** | 152/152 | 158/197 | 170/170 | 143/145 | 246/252 | 156/156 | 148/154 | 249/249 | 169/173 | 136/136 | 119/153 | 232/232 | 212/222 | 173/219 | 233/285 |
| **164** | 164/188 | 158/197 | 158/158 | 143/145 | 248/248 | 156/200 | 148/154 | 247/247 | 165/169 | 150/162 | 119/153 | 232/270 | 222/238 | 173/219 | 233/261 |
| **165** | 188/202 | 158/197 | 158/170 | 145/145 | 238/248 | 200/200 | 150/154 | 247/253 | 165/169 | 150/162 | 119/119 | 270/270 | 188/238 | 171/173 | 233/287 |
| **166** | 188/202 | 158/197 | 158/170 | 143/145 | 238/252 | 156/190 | 148/154 | 249/255 | 147/165 | 128/136 | 119/153 | 268/270 | 188/224 | 173/219 | 261/285 |
| **167** | 188/194 | 197/197 | 158/182 | 143/145 | 238/248 | 156/190 | 148/148 | 247/255 | 165/165 | 128/152 | 119/119 | 232/270 | 188/238 | 173/173 | 233/235 |
| **168** | 188/188 | 197/197 | 158/170 | 141/143 | 238/238 | 156/200 | 148/148 | 249/253 | 169/169 | 128/148 | 119/119 | 232/232 | 188/188 | 173/173 | 233/285 |
| **169** | 164/188 | 197/197 | 158/182 | 143/145 | 238/248 | 200/204 | 148/148 | 247/253 | 169/169 | 150/150 | 119/119 | 232/270 | 188/238 | 173/173 | 233/233 |
| **170** | 152/188 | 197/197 | 170/182 | 145/145 | 238/248 | 156/190 | 148/148 | 219/255 | 165/169 | 128/156 | 119/119 | 232/270 | 188/222 | 173/173 | 235/285 |
| **171** | 188/188 | 197/197 | 158/182 | 143/145 | 248/248 | 190/200 | 148/154 | 247/249 | 165/165 | 150/150 | 119/153 | 232/270 | 222/238 | 173/173 | 261/287 |
| **172** | 164/200 | 197/197 | 158/158 | 127/145 | 236/238 | 156/190 | 148/156 | 247/251 | 165/175 | 142/150 | 117/119 | 252/270 | 188/188 | 173/217 | 235/261 |
| **173** | 164/188 | 197/197 | 158/158 | 143/145 | 248/248 | 156/156 | 148/148 | 247/247 | 165/169 | 150/164 | 119/119 | 270/270 | 222/222 | 173/173 | 261/261 |
| **174** | 188/206 | 158/197 | 158/174 | 145/161 | 248/256 | 156/204 | 108/148 | 219/247 | 147/169 | 150/168 | 119/147 | 232/270 | 188/232 | 171/173 | 233/235 |
| **175** | 188/188 | 197/197 | 158/158 | 143/145 | 248/248 | 156/202 | 148/154 | 247/247 | 165/169 | 152/152 | 119/119 | 232/232 | 222/238 | 173/173 | 261/261 |
| **176** | 152/188 | 158/197 | 170/170 | 145/145 | 238/248 | 156/200 | 148/148 | 249/253 | 169/169 | 128/172 | 119/119 | 232/270 | 188/188 | 171/173 | 233/285 |
| **177** | 152/152 | 158/197 | 170/170 | 143/145 | 246/252 | 156/156 | 148/154 | 249/249 | 169/173 | 136/136 | 119/153 | 232/232 | 212/222 | 173/219 | 233/285 |
| **178** | 152/202 | 170/188 | 158/176 | 145/145 | 238/238 | 156/156 | 154/154 | 249/259 | 169/173 | 136/136 | 153/153 | 232/232 | 224/224 | 177/217 | 235/235 |
| **179** | 152/188 | 158/197 | 170/172 | 145/147 | 238/248 | 156/202 | 154/154 | 247/249 | 147/175 | 150/172 | 153/155 | 232/268 | 188/238 | 173/173 | 235/285 |
| **180** | 202/202 | 158/158 | 172/172 | 143/143 | 286/286 | 196/196 | 154/154 | 249/249 | 147/147 | 148/148 | 141/141 | 232/232 | 234/234 | 171/171 | 235/235 |
| **181** | 152/194 | 158/191 | 158/170 | 107/125 | 226/238 | 156/156 | 108/154 | 219/249 | 175/185 | 138/154 | 153/165 | 232/232 | 190/190 | 185/219 | 235/235 |
| **182** | 152/202 | 158/170 | 170/176 | 125/145 | 238/248 | 156/156 | 108/154 | 219/247 | 169/169 | 136/172 | 151/151 | 232/270 | 222/222 | 217/219 | 285/287 |
| **183** | 176/182 | 176/176 | 176/176 | 133/133 | 214/214 | 182/182 | 130/146 | 249/253 | 175/185 | 138/146 | 161/161 | 262/262 | 190/190 | 177/177 | 235/237 |
| **184** | 152/194 | 158/191 | 158/170 | 107/125 | 226/238 | 156/156 | 108/154 | 219/249 | 175/185 | 138/154 | 153/165 | 232/232 | 190/190 | 185/219 | 235/235 |
| **185** | 152/200 | 176/197 | 172/176 | 125/145 | 226/236 | 198/200 | 112/154 | 249/251 | 147/173 | 138/174 | 141/153 | 232/252 | 190/190 | 171/217 | 235/237 |
| **186** | 186/206 | 158/197 | 158/172 | 125/149 | 236/292 | 194/204 | 156/158 | 249/249 | 147/147 | 138/144 | 143/147 | 232/252 | 188/194 | 171/179 | 235/235 |
| **187** | 152/152 | 170/170 | 170/176 | 125/125 | 238/238 | 194/200 | 148/150 | 249/249 | 171/175 | 138/162 | 153/153 | 238/266 | 238/242 | 179/219 | 235/287 |
| **188** | 204/210 | 188/188 | 170/188 | 109/125 | 236/238 | 158/198 | 156/156 | 251/257 | 171/173 | 148/154 | 117/117 | 252/264 | 188/240 | 171/179 | 235/237 |
| **189** | 152/202 | 158/170 | 170/176 | 125/145 | 238/248 | 156/156 | 108/154 | 219/247 | 169/169 | 136/172 | 151/151 | 232/270 | 222/222 | 217/219 | 285/287 |
| **190** | 152/186 | 197/197 | 176/176 | 145/145 | 226/250 | 156/198 | 108/108 | 253/257 | 171/171 | 142/154 | 143/143 | 232/232 | 234/234 | 171/171 | 237/239 |
| **191** | 152/164 | 158/197 | 158/170 | 141/145 | 238/252 | 190/200 | 148/154 | 247/255 | 169/169 | 150/172 | 119/157 | 232/270 | 188/188 | 171/173 | 261/261 |
| **192** | 164/206 | 158/197 | 158/158 | 125/143 | 250/290 | 156/156 | 154/154 | 247/253 | 147/169 | 138/164 | 153/153 | 232/232 | 190/222 | 217/219 | 235/261 |
| **193** | 164/194 | 170/197 | 160/170 | 139/169 | 236/246 | 156/198 | 118/156 | 251/257 | 171/173 | 150/150 | 117/147 | 232/252 | 222/238 | 173/217 | 235/285 |
| **194** | 192/200 | 158/170 | 158/172 | 125/147 | 284/290 | 156/196 | 158/158 | 249/249 | 147/175 | 142/150 | 141/141 | 232/268 | 192/234 | 171/171 | 235/235 |
| **195** | 194/204 | 158/158 | 158/158 | 125/125 | 248/248 | 156/156 | 156/156 | 219/249 | 171/173 | 124/146 | 117/117 | 264/264 | 190/190 | 171/171 | 237/237 |
| **196** | 152/152 | 170/170 | 158/158 | 145/145 | 250/250 | 156/156 | 140/140 | 257/257 | 147/147 | 164/164 | 143/143 | 254/254 | 188/188 | 173/173 | 237/237 |
| **197** | 152/180 | 176/197 | 170/172 | 141/145 | 248/252 | 156/202 | 148/154 | 251/253 | 169/175 | 154/172 | 119/119 | 232/232 | 188/188 | 173/219 | 237/285 |
| **198** | 180/188 | 176/197 | 170/172 | 143/145 | 238/252 | 156/204 | 148/154 | 247/251 | 169/175 | 128/154 | 119/119 | 232/232 | 188/188 | 173/219 | 237/285 |
| **199** | 200/206 | 158/197 | 158/158 | 125/125 | 236/290 | 156/156 | 156/158 | 249/251 | 147/175 | 140/144 | 117/141 | 232/252 | 188/192 | 171/217 | 235/235 |
| **200** | 200/206 | 197/197 | 158/158 | 125/125 | 236/290 | 156/156 | 156/156 | 251/251 | 147/175 | 138/142 | 117/117 | 232/252 | 192/192 | 217/217 | 235/235 |
| **201** | 164/186 | 158/197 | 158/176 | 135/145 | 238/248 | 190/202 | 148/148 | 247/251 | 169/173 | 150/150 | 119/155 | 270/270 | 188/194 | 171/171 | 235/261 |
| **202** | 190/190 | 176/176 | 176/176 | 125/125 | 226/226 | 198/198 | 152/152 | 249/249 | 169/169 | 188/188 | 153/153 | 266/266 | 188/188 | 177/177 | 237/237 |
| **203** | 188/188 | 170/170 | 170/170 | 125/125 | 244/244 | 198/198 | 152/152 | 249/249 | 147/147 | 190/190 | 153/153 | 232/266 | 188/188 | 217/217 | 285/285 |
| **204** | 152/152 | 170/170 | 176/176 | 147/147 | 226/244 | 196/196 | 156/160 | 219/219 | 171/171 | 156/156 | 117/155 | 250/250 | 188/188 | 171/171 | 285/285 |
| **205** | 152/180 | 158/158 | 160/176 | 133/145 | 244/244 | 196/196 | 160/160 | 257/257 | 171/171 | 136/146 | 117/117 | 254/254 | 188/190 | 171/171 | 235/235 |
| **206** | 164/188 | 170/197 | 170/182 | 139/145 | 226/248 | 198/200 | 148/156 | 249/253 | 169/173 | 150/166 | 117/119 | 266/270 | 188/188 | 173/217 | 233/235 |
| **207** | 152/184 | 158/197 | 170/182 | 125/143 | 238/252 | 156/200 | 148/154 | 219/247 | 169/169 | 136/146 | 119/153 | 232/232 | 222/224 | 171/173 | 261/285 |
| **208** | 188/206 | 158/197 | 158/158 | 125/141 | 236/252 | 156/200 | 154/158 | 247/253 | 147/169 | 138/172 | 141/157 | 232/232 | 190/224 | 171/173 | 235/285 |
| **209** | 188/188 | 197/197 | 182/182 | 141/143 | 248/248 | 156/200 | 148/154 | 253/253 | 169/169 | 150/172 | 119/157 | 232/232 | 224/238 | 173/173 | 233/285 |
| **210** | 182/200 | 170/170 | 170/172 | 125/125 | 236/244 | 158/198 | 112/140 | 249/249 | 171/173 | 148/148 | 141/153 | 266/266 | 192/240 | 179/179 | 237/285 |
| **211** | 188/206 | 158/158 | 170/172 | 145/147 | 248/286 | 194/200 | 108/148 | 247/249 | 147/169 | 128/168 | 147/153 | 232/270 | 188/234 | 171/171 | 235/261 |
| **212** | 152/202 | 188/197 | 158/176 | 125/155 | 236/236 | 198/206 | 156/156 | 249/249 | 171/173 | 142/166 | 117/117 | 268/268 | 246/250 | 171/217 | 237/285 |
| **213** | 194/210 | 188/197 | 170/170 | 137/147 | 236/238 | 198/198 | 156/158 | 249/271 | 171/171 | 148/156 | 117/153 | 252/266 | 190/222 | 173/179 | 235/237 |
| **214** | 188/188 | 158/158 | 170/170 | 143/145 | 248/248 | 156/156 | 154/154 | 219/219 | 147/147 | 152/152 | 153/153 | 266/266 | 190/190 | 219/219 | 285/285 |
| **215** | 204/204 | 170/170 | 158/158 | 145/145 | 226/226 | 156/156 | 140/140 | 257/259 | 147/171 | 158/158 | 141/141 | 268/268 | 188/192 | 173/173 | 237/289 |
| **216** | 200/200 | 197/197 | 172/172 | 161/161 | 236/236 | 200/200 | 148/148 | 267/267 | 147/147 | 128/128 | 151/151 | 266/266 | 240/240 | 171/171 | 287/287 |
| **217** | 152/188 | 170/197 | 176/176 | 123/147 | 226/290 | 156/198 | 142/154 | 251/257 | 147/171 | 152/168 | 117/153 | 252/266 | 188/222 | 217/217 | 235/235 |
| **218** | 188/194 | 170/197 | 170/176 | 139/147 | 226/236 | 194/198 | 156/156 | 249/257 | 171/173 | 148/166 | 117/117 | 252/266 | 188/222 | 173/217 | 235/235 |
| **219** | 152/182 | 158/188 | 170/170 | 125/145 | 238/246 | 156/156 | 154/154 | 219/247 | 169/169 | 136/146 | 151/153 | 232/232 | 222/222 | 171/217 | 261/287 |
| **220** | 152/152 | 170/170 | 170/170 | 141/147 | 244/244 | 196/196 | 160/160 | 219/257 | 169/169 | 136/136 | 117/117 | 254/254 | 190/190 | 219/219 | 235/285 |
| **221** | 152/188 | 158/158 | 158/170 | 125/145 | 236/248 | 156/156 | 154/158 | 219/249 | 147/169 | 150/154 | 117/153 | 232/232 | 190/190 | 171/219 | 261/261 |

**S6 Tab. Number of alleles per locus and diversity index detected by 15 polymorphic SSRs among 221 peach accessions.**

| SSR | Tm (℃) | No. of specific alleles | No. of alleles | I |
| --- | --- | --- | --- | --- |
| SSR43 | 60 | 3 | 19 | 0.148 |
| SSR73 | 60 | 1 | 8 | 0.249 |
| SSR93 | 60 | 1 | 9 | 0.232 |
| SSR96 | 60 | 5 | 22 | 0.112 |
| SSR107 | 60 | 1 | 19 | 0.168 |
| SSR113 | 60 | 3 | 13 | 0.149 |
| SSR120 | 60 | 1 | 16 | 0.291 |
| SSR125 | 60 | 4 | 10 | 0.124 |
| SSR133 | 60 | 2 | 8 | 0.181 |
| SSR152 | 60 | 5 | 26 | 0.146 |
| SSR169 | 60 | 2 | 13 | 0.21 |
| SSR179 | 60 | 2 | 10 | 0.25 |
| SSR181 | 60 | 3 | 10 | 0.157 |
| SSR183 | 60 | 1 | 10 | 0.225 |
| SSR184 | 60 | 2 | 17 | 0.254 |

**S7 Tab. Polymorphism comparisions among 36 accessions amplified using SSR markers developed in this study and reported previously.**

|  | SSR marker | N_A_ | N_PA_ | I | G_D_ |
| --- | --- | --- | --- | --- | --- |
| SSR markers developed in this study | SSR43 | 15 | 4 | 0.1922 | 0.1003 |
|  | SSR73 | 6 | 1 | 0.3579 | 0.22137 |
|  | SSR93 | 8 | 1 | 0.296 | 0.1727 |
|  | SSR96 | 18 | 12 | 0.1528 | 0.0792 |
|  | SSR107 | 14 | 6 | 0.203 | 0.1099 |
|  | SSR113 | 11 | 4 | 0.2226 | 0.123 |
|  | SSR120 | 13 | 3 | 0.2176 | 0.1173 |
|  | SSR125 | 9 | 2 | 0.2752 | 0.1571 |
|  | SSR133 | 7 | 0 | 0.3189 | 0.2096 |
|  | SSR152 | 22 | 10 | 0.1536 | 0.0747 |
|  | SSR169 | 10 | 2 | 0.2461 | 0.1362 |
|  | SSR179 | 8 | 2 | 0.2814 | 0.1616 |
|  | SSR181 | 13 | 4 | 0.215 | 0.1153 |
|  | SSR183 | 9 | 3 | 0.2866 | 0.1248 |
|  | SSR184 | 8 | 1 | 0.3168 | 0.1846 |
|  | total | 171 | 55 |  |  |
|  | Mean | 11.4 | 3.7 | 0.249 | 0.1391 |
| SSR markers reported previously | BPPCT008 | 15 | 5 | 0.1921 | 0.1005 |
|  | BPPCT009 | 7 | 0 | 0.302 | 0.1784 |
|  | BPPCT015 | 20 | 14 | 0.1354 | 0.0674 |
|  | BPPCT017 | 12 | 5 | 0.2147 | 0.1177 |
|  | BPPCT020 | 6 | 1 | 0.3378 | 0.2072 |
|  | BPPCT034 | 7 | 2 | 0.3044 | 0.1833 |
|  | CPPCT003 | 9 | 4 | 0.2364 | 0.1364 |
|  | CPPCT005 | 14 | 3 | 0.2131 | 0.1171 |
|  | CPPCT013 | 4 | 0 | 0.3971 | 0.2478 |
|  | CPPCT022 | 15 | 8 | 0.1667 | 0.0845 |
|  | CPPCT031 | 10 | 5 | 0.2246 | 0.1287 |
|  | UDP001 | 9 | 4 | 0.2732 | 0.1612 |
|  | UDP008 | 6 | 3 | 0.2519 | 0.1475 |
|  | UDP407 | 9 | 3 | 0.3127 | 0.1463 |
|  | UDP409 | 10 | 5 | 0.2185 | 0.123 |
|  | total | 153 | 62 |  |  |
|  | Mean | 10.2 | 4.1 | 0.252 | 0.1431 |

N_A,_ the number of alleles per locus; N_PA,_ the number of private alleles; I, Shannon’s information index; G_D_, gene diversity.
